# Sustainability in Health care by Allocating Resources Effectively (SHARE) 8: developing, implementing and evaluating an evidence dissemination service in a local healthcare setting

**DOI:** 10.1186/s12913-018-2932-1

**Published:** 2018-03-02

**Authors:** Claire Harris, Marie Garrubba, Angela Melder, Catherine Voutier, Cara Waller, Richard King, Wayne Ramsey

**Affiliations:** 1 School of Public Health and Preventive Medicine, Monash University, Melbourne, VIC Australia; 2 Centre for Clinical Effectiveness, Monash Health, Melbourne, VIC Australia; 3 Health Sciences Library, Melbourne Health, Melbourne, VIC Australia; 4 Medicine Program, Monash Health, Melbourne, VIC Australia; 5 Medical Services and Quality, Monash Health, Melbourne, VIC Australia

**Keywords:** Evidence-based practice, Evidence-informed decision-making, Evidence products and services, Evidence dissemination, Knowledge broker, Current awareness services, Current awareness alerts, Needs assessment, Needs analysis, Information needs

## Abstract

**Background:**

This is the eighth in a series of papers reporting Sustainability in Health care by Allocating Resources Effectively (SHARE) in a local healthcare setting. The SHARE Program was a systematic, integrated, evidence-based program for disinvestment within a large Australian health service. One of the aims was to explore methods to deliver existing high quality synthesised evidence directly to decision-makers to drive decision-making proactively. An Evidence Dissemination Service (EDS) was proposed. While this was conceived as a method to identify disinvestment opportunities, it became clear that it could also be a way to review all practices for consistency with current evidence. This paper reports the development, implementation and evaluation of two models of an in-house EDS.

**Methods:**

Frameworks for development of complex interventions, implementation of evidence-based change, and evaluation and explication of processes and outcomes were adapted and/or applied. Mixed methods including a literature review, surveys, interviews, workshops, audits, document analysis and action research were used to capture barriers, enablers and local needs; identify effective strategies; develop and refine proposals; ascertain feedback and measure outcomes.

**Results:**

Methods to identify, capture, classify, store, repackage, disseminate and facilitate use of synthesised research evidence were investigated. In Model 1, emails containing links to multiple publications were sent to all self-selected participants who were asked to determine whether they were the relevant decision-maker for any of the topics presented, whether change was required, and to take the relevant action. This voluntary framework did not achieve the aim of ensuring practice was consistent with current evidence. In Model 2, the need for change was established prior to dissemination, then a summary of the evidence was sent to the decision-maker responsible for practice in the relevant area who was required to take appropriate action and report the outcome. This mandatory governance framework was successful. The factors influencing decisions, processes and outcomes were identified.

**Conclusion:**

An in-house EDS holds promise as a method of identifying disinvestment opportunities and/or reviewing local practice for consistency with current evidence. The resource-intensive nature of delivery of the EDS is a potential barrier. The findings from this study will inform further exploration.

**Electronic supplementary material:**

The online version of this article (10.1186/s12913-018-2932-1) contains supplementary material, which is available to authorized users.

## About share

*This is the eighth in a series of papers reporting Sustainability in Health care by Allocating Resources Effectively (SHARE). The SHARE program is an investigation of concepts, opportunities, methods and implications for evidence-based investment and disinvestment in health technologies and clinical practices in a local healthcare setting. The papers in this series are targeted at clinicians, managers, policy makers, health service researchers and implementation scientists working in this context. This paper reports the development, implementation and evaluation of two models of an Evidence Dissemination Service in a local healthcare setting and discusses the factors that influenced decisions, processes and outcomes.*

## Background

Monash Health, a large academic health service network in Melbourne Australia, established the ‘Sustainability in Health care by Allocating Resources Effectively’ (SHARE) Program to investigate an organisation-wide, systematic, integrated, evidence-based approach to disinvestment. The SHARE Program was undertaken by the Centre for Clinical Effectiveness (CCE), an in-house resource to facilitate Evidence Based Practice (EBP). The focus of the program was on how a health service guides, directs and makes decisions at organisational level, in contrast to the decisions made by individual health practitioners in clinical practice.

Although there is no clear single definition, disinvestment is generally understood to be removal or restriction of health technologies and clinical practices (TCPs) that are unsafe or of little benefit [1]. In most published examples, disinvestment has been undertaken as an independent activity. However, following review of the literature and consultation with local stakeholders, Monash Health decision-makers felt that undertaking disinvestment in isolation from other decision-making processes was artificial and possibly counterproductive [2]. The scope was revised to consider disinvestment within the spectrum of all resource allocation decisions covering investment in new, continuation of existing, and disinvestment from current activities [2]. These decisions were focused in two areas: 1) allocation of funding, such as purchasing of drugs and clinical consumables and capital expenditure on building and equipment, and 2) allocation of non-monetary resources through guidelines and protocols which stipulate use of drugs or equipment, recommend diagnostic tests, prioritise staff time, specify referral mechanisms and allocate capacity in clinics, operating rooms and other facilities.

**Fig. 1:**
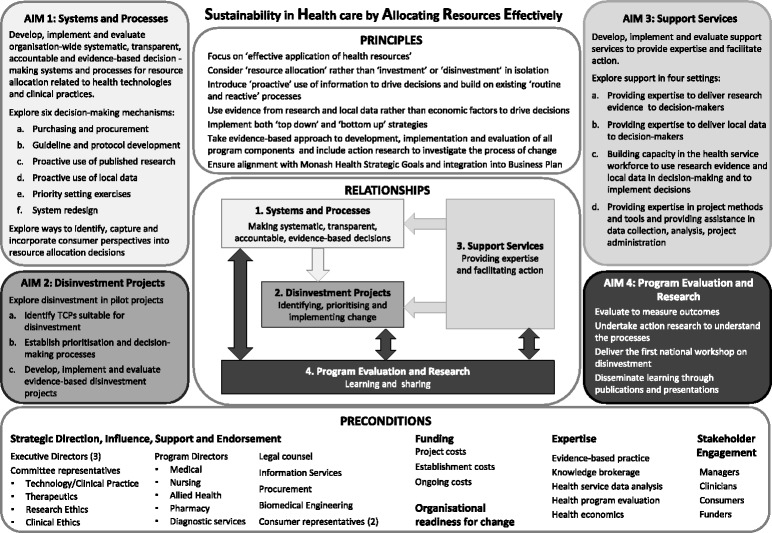
Overview of Phase Two of the SHARE Program (reproduced with permission from Harris et al. [2])

The SHARE Program was undertaken in two phases. Phase One explored concepts and practices related to disinvestment to understand the implications for a local health service [3–5] and, based on this information, identified potential settings and methods for decision-making [2]. Phase Two developed, implemented and evaluated the proposed methods to determine which were sustainable, effective and appropriate at Monash Health [6, 7]. The four aims of Phase Two are outlined in Fig. 1.

The first aim was to explore systems and processes for decision-making relating to TCPs. Objectives under this aim included investigation of methods for proactive access and utilisation of existing high quality research and health service data to initiate change [3]. Local research at Monash Health confirmed the findings of other studies that health service staff report lack of time, knowledge, skills and resources as barriers to searching for information, accessing it and appraising it for quality and relevance; and that evidence was not used systematically or proactively to drive decisions [4, 7–18]. The second aim was to pilot disinvestment projects [6] and Monash Health staff reported lack of skills and confidence in implementing and evaluating change. Local responses were also consistent with studies that identified a need for dedicated resources and in-house *“resource centres”* to address these barriers in the context of resource allocation [19–23]. Four support services were proposed to facilitate the SHARE aims: an Evidence Service, Data Service, Capacity Building Service and Project Support Service. Piloting of these services became Aim 3. Details of establishment of the Data, Capacity Building and Project Support Services are reported in Paper 7 in this series [7].

Research evidence underpinned two fundamental elements of the SHARE Program. The first was evidence-based decision-making (EBDM), one of the foundation principles of the program. The second was proactive use of the increasing body of literature about practices that have been demonstrated to be harmful, of little or no benefit, or where a more effective or cost-effective alternative is available to identify opportunities and initiate evidence-based decisions for disinvestment, one of the objectives to be explored within Aim 1 (Fig. 1) [3].

CCE already provided an evidence service which facilitated EBDM ‘reactively’, in response to requests from decision-makers, by undertaking systematic reviews to inform organisational decisions and delivering a range of training programs [24]. Hence the new SHARE Evidence Service was conceptualised as an Evidence Dissemination Service (EDS) to ‘proactively’ identify, capture and deliver existing research evidence directly to decision-makers to instigate disinvestment decisions by identifying opportunities for change that they were previously unaware of.

This proactive approach of *“pushing”* research out to potential users has been advocated as a tool to increase evidence uptake [14, 25–30] and an enabler to effective resource allocation [21, 31, 32]. Research into methods to routinely and systematically capture, adapt and reframe information, then circulate it internally within a health service has been proposed [33]; as has targeted dissemination of synthesised evidence directly to decision-makers [34].

In their review of diffusion of innovations in health services, Greenhalgh and colleagues ask *“How can we improve the absorptive capacity of service organizations for new knowledge? In particular, what is the detailed process by which ideas are captured from outside, circulated internally, adapted, reframed, implemented, and routinized in a service organization, and how might this process be systematically enhanced?”* [33]. This case study presents two models of capturing, disseminating and utilising new knowledge through a systematic approach in a local health service.

While the EDS was conceived as a method of identifying disinvestment opportunities, it quickly became clear that this could be a way to confirm that practices at Monash Health were consistent with current evidence through investment, disinvestment or modification.

Monash Health is a public network of six acute hospitals, subacute and rehabilitation services, mental health and community health services, and residential aged care [35]. Australian public hospitals operate under a state-allocated activity-based fixed-budget model of financing [36]. Staff are salaried and services are provided free of charge. An overview of the SHARE Program, a guide to the SHARE publications and further details about Monash Health (previously Southern Health) and CCE are provided in the first paper in this series [24] and a summary of the findings are in the final paper [37].

### Aims

The aim of the EDS was to deliver research evidence directly to clinicians, managers and policy makers for use in decision-making to ensure that allocation of resources at Monash Health was consistent with current evidence.

The aims of this paper are to report the development, implementation and evaluation of two models of an EDS in a local healthcare setting and discuss the factors that influenced decisions, processes and outcomes.

### Research questions

#### Theoretical phase

What are the potential features of an EDS in a local healthcare setting?

#### Modelling phase

How can high quality synthesised evidence be identified, captured, classified, stored, repackaged and disseminated?

How can disseminated evidence be used to enhance current practice and how can use of evidence be reported?

#### Exploratory phase

What were the processes and outcomes of disseminating evidence to self-selected and targeted participants in a voluntary framework (Model 1)?

What were the processes and outcomes of disseminating evidence to designated decision-makers in a mandatory governance framework (Model 2)?

#### Explication

What factors influenced decisions, processes and outcomes?

## Methods

Several of the activities reported in this paper were to develop methods that would be undertaken in subsequent activities. The methods reported in this section are those determined a priori. Methods developed during the course of the investigation to inform future activities are reported in the Results section.

### Framework for design and evaluation of complex interventions

A three-phased approach was used in the development of the EDS. This approach is consistent with the UK Medical Research Council (MRC) framework for design and evaluation of complex interventions [38]. The EDS meets the MRC definition of a complex intervention: it is composed of multiple components which act both independently and inter-dependently. The components include behaviours, parameters of behaviours and methods of organising and delivering those behaviours [38]. The objectives of each phase are:

Theoretical: To establish the theoretical basis that suggests the intervention will have the expected outcomes.

Modelling: To delineate and explore the intervention’s components, how they inter-relate and how they influence outcomes; may include preliminary testing if appropriate.

Exploratory: To implement the intervention, potentially experiment by varying components, and identify constant and variable components to enable replication and further testing.

### Model for evidence-based change

The EDS was developed using the SEAchange model for Sustainable, Effective and Appropriate change in health services developed by CCE and modified for use in this context [39]. The model involves four steps: identify the need for change, develop an intervention to meet the need, implement the intervention and evaluate the change. Each step is underpinned by the principles of evidence-based practice to ensure that the best available evidence from research and local data, the experience and expertise of health service staff and the values and perspectives of consumers are taken into account.

#### Step 1. Identify need for change

A literature review, surveys, interviews and a workshop were undertaken to elicit the information needs of decision-makers, identify barriers and enablers to using research evidence in decision-making in local healthcare services, and gather baseline data for evaluation. A wide range of senior decision-makers representing all health professional groups, clinical programs, campuses and relevant committees were invited to participate. Details of data collection methods and sources are provided in Additional file 1: Section 1.

Final interview and workshop notes were analysed thematically in MS Word, Excel and/or Nvivo [40] by either identification of emergent themes or categorisation according to the aims outlined in the individual project protocols (Additional file 1: Section 1). Survey totals and percentages were calculated.

#### Step 2. Develop intervention

Using the principles of evidence-based change [39], the SHARE team worked with stakeholders to synthesise the findings from the literature and local research and develop draft proposals.

Feedback on draft proposals was sought from senior clinical decision-makers (Nursing Executive Team, all Medical Program Directors and the General Manager of Allied Health) via structured individual and group discussions, and other health service staff via invitations to provide input distributed through the ‘All staff’ email list and informal discussions with staff interacting with the project team (Additional file 1: Section 2).

Proposals are more likely to be successful if they have certain characteristics [33, 41, 42] and new initiatives are more likely to be sustainable if there is appropriate and adequate provision of critical factors to achieve and maintain the proposed components and activities [43]. These characteristics, assessed using a checklist for success and sustainability (Additional file 1: Section 2), and opportunities to avoid duplication and integrate new systems and processes into existing infrastructure were considered in development of the two models of the EDS.

Program logic including consideration of assumptions, inputs, activities, outputs and outcomes required to achieve objectives was used in development of the intervention, implementation and evaluation plans.

Structured workshops with senior managers, clinicians and consumers were held for discussion, refinement and decision-making related to draft proposals (Additional file 1: Section 2). Strategic direction, governance, executive sponsorship and senior management support, clinical perspectives and technical advice were provided initially by an EDS Advisory Group and later by the SHARE Steering Committee (Additional file 1: Section 2).

Decisions regarding methods for development and delivery of the new evidence products were made by the CCE team with expertise in evidence synthesis, knowledge brokerage and EBP.

The overall project and both proposed models were endorsed by the Executive Management Team and Monash Health Board.

#### Step 3. Implement intervention

Planned implementation activities included engaging all stakeholders, identifying what is already known about practice change in the topic area from the literature and local knowledge, undertaking an analysis of local barriers and enablers, developing an implementation plan using strategies to minimise barriers and build on enablers, piloting and revising as required, and implementing in full [39].

Barriers and enablers to use of research evidence in decisions at Monash Health were ascertained in the surveys and interviews noted above. Barriers and enablers to delivery and use of the EDS were determined from the evaluation and action research methods noted below.

Two variations of the intervention were implemented; modifications were based on findings from evaluation and ongoing action research activities.

#### Step 4. Evaluate change

An evaluation framework and plan, including evaluation of the EDS, was developed for the overall SHARE Program and included evaluation domains, audience, scope, evaluation questions, outcomes hierarchy, sources of data, methods of collection and analysis, reporting and timelines [44]. More detailed evaluation plans for the EDS were subsequently developed based on the ‘Guide to Monitoring and Evaluating Health Information Products and Services’ [45]. Planned methods included stakeholder surveys, interviews and consultation, feedback sections on Evidence Bulletins, audit of website statistics and document analysis (Additional file 1: Section 3). Details of which methods were used in each of four evaluations reported (two pilot studies, two full implementation studies) are summarised in the relevant sections below.

### Action research

Action research was undertaken to refine the intervention, enable continuous improvement in implementation and evaluation, and collect data for evaluation and explication. The approach taken was based on the “*researcher as facilitator for change*” defined by Meyer: researchers working explicitly with and for people rather than undertaking research on them [46, 47]. In this capacity, CCE staff were both the SHARE project team and the action researchers. An agenda item for ‘Learnings’ was scheduled at the beginning of every team meeting. Participants were invited to consider anything that had affected the project since the last meeting using the framework ‘what worked, what didn’t, why and how it could be improved’. Each issue, its effect on the project, and potential changes that would build on positive outcomes or remove or minimise future problems were discussed. The learnings and actions were documented; actions were assigned, given timeframes and followed up to ensure completion. Project team observations and reflections were used for ongoing improvements to the program components, implementation and evaluation processes, and explication of the influencing factors.

### Explication

Factors influencing decisions, processes and outcomes were identified and analysed to understand their effect and the resulting implications.

Factors that influenced initial decisions in development of the intervention were mapped to the components of the EDS in a synthesis matrix adapted from Wallace et al. [48].

**Fig. 2:**
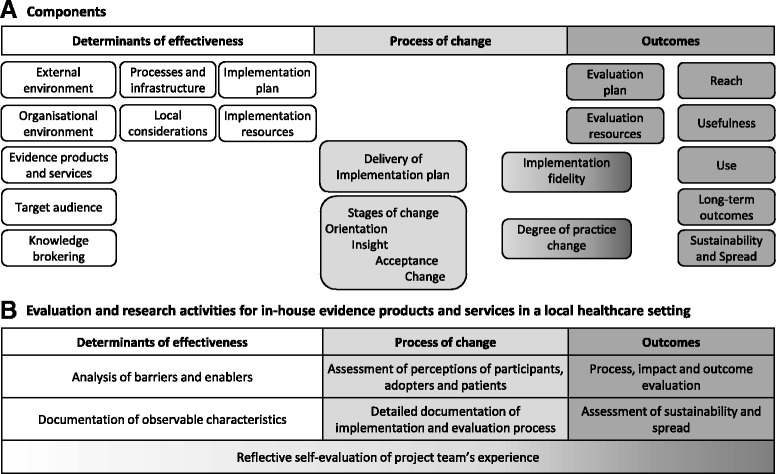
Framework for evaluation and explication of implementation of evidence-based health information products and services (adapted with permission from Harris et al. [49])

**Fig. 3:**
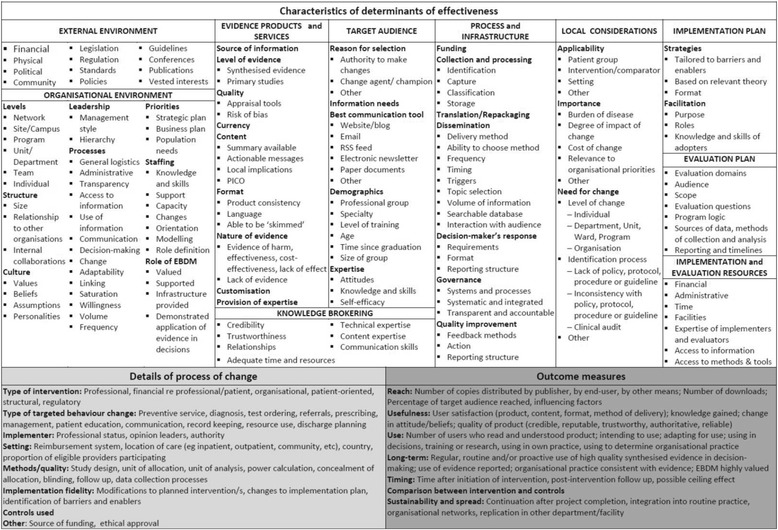
Taxonomy for evaluation and explication of implementation of evidence-based health information products and services (adapted with permission from Harris et al. [49])

Factors that influenced processes and outcomes of implementation and subsequent decisions in revision of the EDS were identified and reported using an existing framework and taxonomy for evaluation and explication of evidence-based innovations [49] which was adapted to investigate delivery of an in-house EDS in the context of a local health service (Figs. 2a and 3). Adaptation of the determinants of effectiveness was based on a framework for knowledge transfer [50] and the process of change and outcome measures were modified using the guide to evaluation of health information products and services [45]. Some details within the taxonomy were also drawn from the work of others [51–55]. The additional domain of ‘Local considerations’ was derived from experiences in development of the EDS discussed below. Details of barriers and enablers, observable characteristics of the determinants of effectiveness, perceptions of participants and adopters, the process of change, and findings from the action research process were documented in minutes, reports, spreadsheets and templates for this purpose (Fig. 2b).

### Alignment of methods

**Fig. 4:**
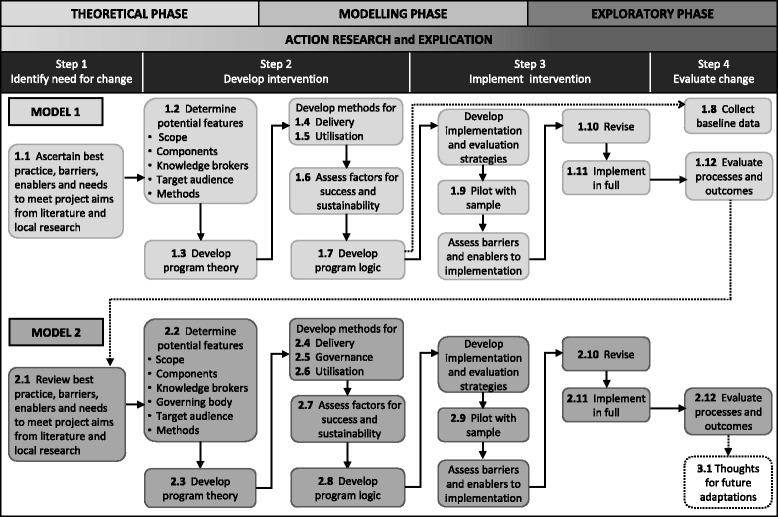
Development, implementation and evaluation of an in-house Evidence Dissemination Service

Figure 4 illustrates how the three phases of the UK MRC framework, the four steps of the SEAchange model and the action research and explication processes align with the activities undertaken in development, implementation and evaluation of the two models.

Some of the planned activities were not completed due to reduction of funding in the final year of the SHARE Program resulting in shortened timelines; details and impact are discussed below.

## Results

Full details of the results of the literature search and response rates and representativeness of participants in the surveys, interviews and workshop are reported in Additional file 1: Section 1.

A systematic search of the literature was undertaken however broad searches resulted in unmanageable numbers of returned articles and narrowing the search returned none. Since the purpose of the review was to inform in-house decision-making for development of the EDS, a decision was made to take a pragmatic, iterative approach by accessing relevant publications already known to the project team and following up with simpler searches and pursuing articles from reference lists.

Data were collected from 164 survey respondents representing all campuses, clinical programs and professional disciplines in appropriate proportions; 27 interviewees including representatives of organisation-wide decision-making bodies (e.g.committee chairs), individuals with responsibility for resource allocation decisions as part of their role (e.g. department or unit heads), and members of project teams who had undertaken disinvestment activities; and 18 senior clinicians from a large multi-campus department who participated in a workshop. Draft proposals were refined based on feedback from individual and group interviews, email correspondence and informal discussions with 36 senior decision-makers and other staff representing all campuses, clinical programs and professional disciplines (Additional file 1: Section 2).

Data collected from these activities informed a range of research questions. Findings related to this paper are provided in Additional file 1: Sections 4–16, synthesised to address the research questions and reported below. Findings related to topics not addressed here are reported in other SHARE publications [2, 4, 6, 7].

Following implementation and evaluation, the initial design of the EDS was revised considerably prior to re-implementation and evaluation. Based on the definition of a model as a representation of the relationships between concepts to provide a frame of reference, where the concepts are well defined and the relationships between them are specific so that the model is a representation of the real thing [56], the two designs are reported here as Model 1 and Model 2.

The heading structure reporting the development, implementation and evaluation of the two models corresponds to the numbering of activities in Fig. 4.

### Model 1

In this model, participants enrolled voluntarily to receive Evidence Alerts containing links to multiple publications.

### 1.1 Factors influencing decisions in development of Model 1

Initial decisions regarding scope, components, knowledge brokers, target audience and methods were based on:

- meeting the aims of the SHARE Program
- overcoming or minimising barriers and building on the enablers identified from the literature and local research
- addressing specific requests for content and format from the needs analysis
- available resources

The findings from local research (Additional file 1: Sections 4–7) were consistent with the literature. As expected, the main barriers were lack of time, skills, confidence, resources, support, awareness of and availability of research. Dissemination of evidence to decision-makers, relevance and reliability of research, and organisational support and infrastructure for using evidence in decisions were reported as enablers. Specific needs included provision of expertise, new processes to use evidence proactively, and support that was tailored to the needs of individual units and professional groups.

The barriers, enablers and needs are mapped to the relevant components of the EDS in a synthesis matrix provided in detail in Additional file 1: Section 7a. Each component was based on a solid foundation of research evidence and local data.

### 1.2 Potential features of an EDS in a local healthcare setting

#### Scope

The scope of the EDS was determined by the following decisions.

To avoid wasting time and resources considering information that may not be valid or may not represent a comprehensive view of all the available evidence, only high quality synthesised evidence would be used.

To ensure currency of the information, only recently published evidence would be sourced and disseminated.

To facilitate topic selection by users, and enable dissemination to appropriate target audiences, the selected publications would be classified using multiple categories.

To facilitate utilisation of evidence, publications would be repackaged to reflect the needs of users and active responses from the target audiences would be required.

#### Components

**Fig. 5:**
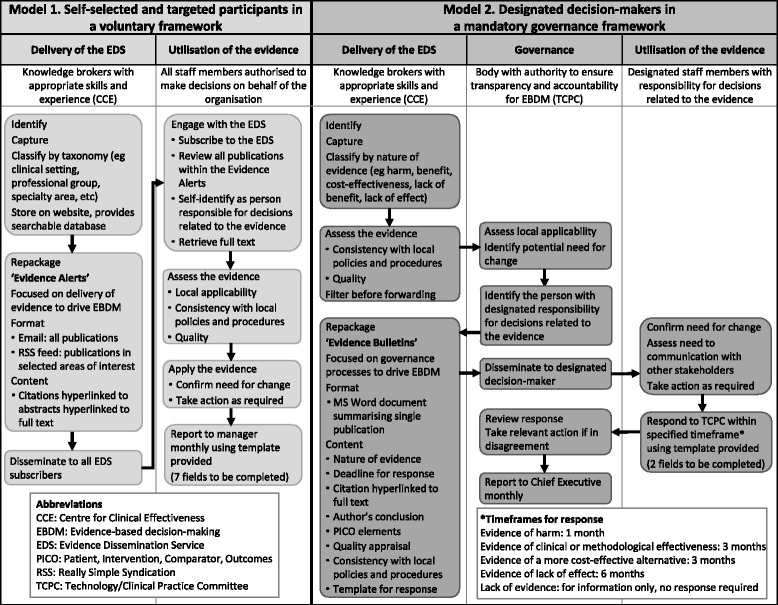
Comparison of stakeholder roles in two models for an in-house Evidence Dissemination Service

Two components of an in-house program to facilitate proactive use of evidence in decision-making were identified: ‘Delivery of the Evidence Dissemination Service’ and ‘Utilisation of the disseminated evidence’ (Fig. 5). The elements in delivery of the evidence were identification, capture, classification and storage of synthesised evidence; translation and repackaging into user-friendly formats; and dissemination to decision-makers. The elements for utilisation of the evidence were engagement with the EDS, and assessment, application and reporting use of the evidence.

#### Knowledge brokers

The EDS team were CCE staff with expertise as systematic reviewers, knowledge brokers, implementers, evaluators and a health librarian. Some had previously been health practitioners, however it was recognised that a practicing clinician should also be involved to ensure correct classification within clinical categories. Based on the SEAchange principle of integrating new initiatives into existing systems and processes [39], the Monash Health Medical Administration Registrar (trainee) was seconded to SHARE. The registrar would benefit from exposure to the processes of EBDM for clinical practice, management and policy-making and the EDS would benefit from their up-to-date clinical knowledge.

#### Target audience

The target audience was defined as individuals and groups authorised to make resource allocation decisions on behalf of the organisation that had been identified in a previous SHARE project [4]. While all Monash Health staff would be invited to subscribe to the EDS broadcasts, relevant department heads and unit managers, plus the 14 committees identified as making resource allocation decisions for TCPs, would be targeted to report on use of evidence from the EDS in their areas of authority.

#### Methods

Determination of the scope and components of an in-house EDS identified that several processing steps were required prior to dissemination. The shortage of published information in most of these areas meant that establishment of an EDS would entail development of methods and tools to identify sources of high quality synthesised evidence, automate the capture process, classify and store materials in useful categories, repackage into suitable formats based on user needs, disseminate to the appropriate target groups, and report use of evidence. An overview of the options considered in development of methods and tools for the individual steps is included in Additional file 1: Section 8.

### 1.3 Program theory

**Fig. 6:**
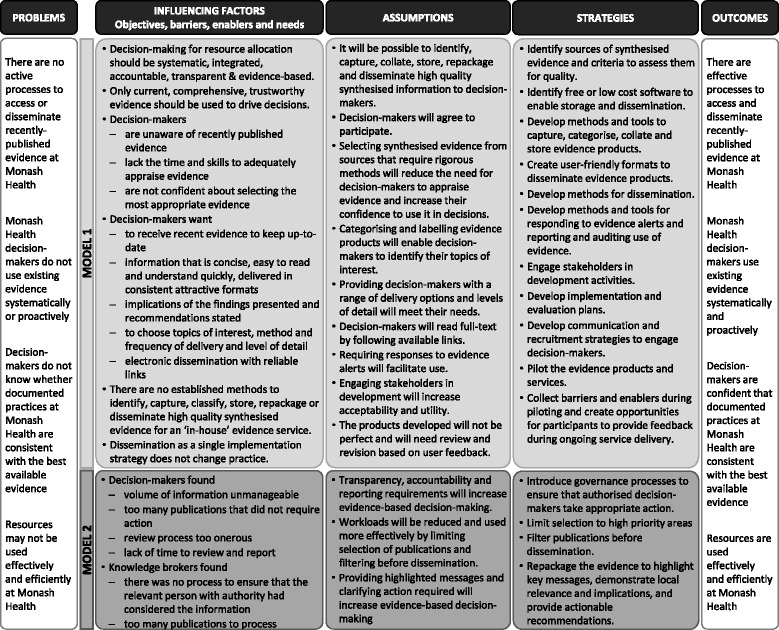
Program theory

Program theory is a way of explaining the anticipated pathway of change by identifying underlying problems, influencing factors, assumptions that underpin the choice of strategies, strategies that will deliver the intended results, and the desired outcomes [57, 58]. To facilitate understanding and replication of the EDS processes and outcomes, the program theory is presented in Fig. 6.

### 1.4 Delivery of the Evidence Dissemination Service

#### Identification

Systematic reviews, health technology assessments (HTAs), evidence-based guidelines, horizon scanning reports, and alerts and recall notices were considered relevant for resource allocation decisions, particularly disinvestment.

It was not possible within the available project resources to identify and capture all synthesised evidence or to critically appraise each individual publication to determine those of high quality. Hence a decision was made to limit the searches to electronic sources of synthesised evidence where the publication process required rigorous methods; in effect critically appraising the methods required by the publisher as a proxy for the methods undertaken by the authors.

Definitions of these evidence products, details of the appraisal criteria used and the sources accessed for the EDS are included in Additional file 1: Sections 9 and 10.

#### Capture

With limited resources it was important to automate the capture process as much as possible. The EDS project officer subscribed to receive information from email alerting services and Really Simple Syndication (RSS) feeds when available and scheduled dates for regular manual capture from the other sites.

#### Classification

Publications were classified using a taxonomy based on existing definitions from recognised health resources [59–62]. New categories, with definitions for each classification, were developed to meet additional Monash Health needs. Definitions adapted or developed for the EDS taxonomy are outlined in Additional file 1: Section 11.

#### Storage

The EDS team investigated a range of storage technologies. As there was no funding for information technology, the final decision was to use free internet software to create a website, blog, email and RSS feeds and pay a small fee to maintain these facilities free of advertisements. Details of the options considered and reasons for the choice of software are provided in Additional file 1: Section 8.

Only citations, abstracts and links to full text on the publisher’s website were stored. The website was searchable using the tags applied in the classification process so users could find publications based on the categories in the taxonomy. Examples of webpages are provided in Additional file 1: Section 12.

#### Repackaging

Findings from the literature regarding desirable characteristics of evidence products and services are summarised in Table 1 [25–28, 50, 63–67].

**Table 1:** Examples of desirable characteristics of evidence products and services

Characteristics	1*	**2**
PRODUCT Source		
▪ Identify ‘credible’ sources to avoid the target audience spending time appraising methodological quality and merit	✓	✓
▪ Identify sources that the target audience consider trustworthy	✓	✓
Level of evidence		
▪ Transfer evidence from a ‘body of knowledge’ such as systematic reviews, health technology assessments and evidence-based guidelines so the reader has all the available evidence on the topic	✓	✓
Quality and currency		
▪ Ensure information is current and provide a publication date	✓	✓
▪ Ensure information is well written, concise, easily understood, well organised, convenient to access, clinically applicable and relevant, and linked to other relevant high-quality documents		✓
Content		
▪ Word the title to engage the target audience (eg as a question, with a solution-orientation)		
▪ Present findings using an ‘inverted pyramid’ (eg bulleted key messages, executive summary, full report)		✓
▪ Highlight ‘take-home’ messages from the review, particularly decision-relevant information (eg benefits, harms)		✓
▪ Present ‘ideas’ rather than ‘data’		✓
▪ List actionable recommendations in order of effectiveness and link to supporting evidence		✓
▪ Articulate the implications of the findings to policy and practice, and potential outcomes of implementation		✓
▪ Frame the findings and implications within the local, state/provincial, or national context		✓
▪ Highlight the characteristics of the participants in the included studies and the contexts in which the studies were conducted that might influence local applicability and/or raise equity considerations		✓
▪ Limit discussion of methods, if required report in an appendix		✓
Format		
▪ Deliver a product/service that looks familiar and works in a consistent manner each time	✓	✓
▪ Use a format that is visually interesting and presented attractively	✓	✓
▪ Use a format that is easy to scan quickly	✓	✓
▪ Link summaries to full text	✓	✓
▪ Use language appropriate to the target audience, jargon-free, with technical language restricted to an appendix		✓
▪ Ensure electronic sources run smoothly and links work as expected	✓	✓
Customisation		
▪ Customise the information to meet the needs of the target audience	✓	✓
PROCESSES AND INFRASTRUCTURE		
▪ Provide the target audience with choice and control over − topic selection (eg their areas of interest, specialty, profession domain or clinical setting)	✓	
− amount of detail (eg abstract, summary, full text)	✓	
− method (eg electronic, hard copy, Internet)	✓	
− frequency of delivery (eg at time of publication, daily, weekly, monthly)	✓	
▪ Provide a searchable database or registry	✓	
▪ Use interactive methods	✓	✓
▪ Engage the target audience in providing online commentaries about specific reviews or review-derived products		
▪ Provide online briefings (eg webinars) about specific reviews or review-derived products		
▪ Provide face-to-face briefings about specific reviews or review-derived products		
▪ Give presentations about specific reviews or review-derived products coupled with stakeholder commentaries		
KNOWLEDGE BROKERING		
▪ Ensure those who transfer information are seen as credible and trustworthy by the target audience	✓	✓
▪ Engage someone with appropriate skills, preferably from within the practice setting, to repackage information, write summaries, etc		✓

*1 = Model 1, 2 = Model 2

Findings from local survey participants about their preferences for dissemination of research to inform resource allocation decisions are provided in Additional file 1: Section 4. Most respondents wanted to receive critical appraisals and full text articles of both primary and secondary research; fewer wanted abstracts only. A range of responses were received regarding the focus of research content. These were, in descending order of preference, condition specific information (e.g. Diabetes), professional group information (e.g. Emergency Department Nursing), program relevant information (e.g. Mental Health), organisation-wide information (e.g. Infection Control) and unit relevant information (e.g. Newborn Services); however more than half of the respondents selected these within their first three preferences so all would be considered of some importance to the target audience. Email broadcasts were clearly preferred over paper-based options for dissemination of research, with short pdf attachments containing titles and hyperlinks preferred over long pdf attachments with titles, abstracts and hyperlinks.

Publications were repackaged into ‘Evidence Alerts’ where the aim was to drive EBDM by delivering evidence directly to decision-makers. The selected software enabled the titles to be contained within the email to save use of attachments. The titles were hyperlinked to the full citation, including abstract, located further down in the body of the email, and the citation was hyperlinked to the full text (Additional file 1: Section 13). This gave readers flexibility to scan the list of titles easily, to find out more information from the abstract without leaving their email, or to go directly to the original document.

The titles were coded so the reader could identify the type of publication; for example, systematic reviews were identified by the prefix SR (Additional file 1: Section 10).

The initial proposal was to include an overall statement about the findings such as ‘evidence of effectiveness’, ‘evidence of harm’ or ‘lack of evidence’ which would be taken directly from the published article. However, it was frequently difficult to find such statements and, unless we critically appraised each individual article, we could not be confident that the findings or recommendations were valid. Hence, a statement regarding the nature of the evidence was not provided by the EDS.

#### Dissemination

Dissemination was by email and RSS feed to Monash Health staff who had subscribed to the EDS.

Evidence Alerts were emailed every two weeks. They contained all the publications captured by the EDS in the interval since the previous broadcast. Broadcasts were limited to a maximum of 30 publications.

Subscribers who wished to limit the information they received to selected topics of interest could establish an RSS feed based on their desired categories.

### 1.5 Utilisation of disseminated evidence

To achieve the SHARE aim of using proactive EBDM to ensure Monash Health practice was consistent with current evidence would require more than just dissemination of recent publications.

#### Engagement with the EDS

Members of the target audience were required to enrol to receive Evidence Alerts as either emails containing all publications or RSS feeds restricted to their areas of interest, to review the publications within each broadcast, and then, if they identified themselves as the person responsible for organisational decisions related to the topic of a publication, to retrieve the article in full text.

#### Assessment of the evidence

From the full text, subscribers could assess whether the topic was applicable to current practice at Monash Health. If it was applicable, local policies and procedures could be reviewed to ascertain whether documented organisational practice was consistent with the recently published evidence. If it was, no further action would be required. However, if there was no local guidance, or the guidance available was inconsistent with the evidence, change may be required. It would not be appropriate to proceed to changing practice without ensuring that the evidence was valid. Although the sources of synthesised evidence had been assessed as likely to produce high quality publications, this was not an absolute guarantee that either the systematic review, or the evidence it contained, was of high quality. Critical appraisal would be required to verify this.

#### Application of the evidence

If the evidence was found to be valid and the need for change confirmed, the decision-maker would be required to take the appropriate action.

#### Reporting use of evidence

Development of methods and tools for reporting use of evidence disseminated by the EDS was based on factors arising from the local environment and knowledge translation theory.

There were three main considerations in the local environment. Monash Health was committed to EBDM and to promoting use of evidence throughout the organisation. The SHARE Program was focused on an organisation-wide approach; i.e. the EDS would be used to ensure organisational practice, as documented in policies and protocols, was consistent with current evidence. And one of the principles underpinning the program was to integrate new initiatives into existing infrastructure.

There were several considerations from the knowledge translation literature. It was well-established that dissemination alone is not an effective knowledge translation strategy [68]. It had been proposed that the impact of HTAs at the policy level could be increased if they were linked with quality systems such as standards and performance indicators [34]. Regulation, by control or obligation through rules and laws, had been described as potentially one of the most powerful methods of influencing behaviour [69] and was thought to be particularly relevant when considering organisational, rather than individual, responsibilities [16, 70]. Managers are influenced by facilitative and regulatory mechanisms, suggesting that behaviour change in this context requires both support and interventions integrated into organisational infrastructure and policies [16, 71, 72]. Although regulation had been demonstrated to be effective in other complex organisations [70], there was no evidence in hospital settings. However mandatory measures have been well accepted in the healthcare context [16, 33], particularly in the area of patient safety [73].

The desired application of evidence from the EDS by authorised decision-makers was to determine whether change was needed and then adapt practice accordingly. To encourage completion of this process, and to facilitate the organisational responsibility of ensuring practice is consistent with the best available evidence, it was proposed that decision-makers in the target groups be required to report on the actions and outcomes following receipt of an EDS broadcast. This is consistent with definitions of regulation or structural intervention in current classification systems of implementation strategies [74, 75].

Based on the early development work categorising evidence by clinical topics, it was anticipated that managers would receive between one and three publications to review per month.

Monash Health managers were required to provide monthly reports on financial and business indicators. It was proposed that, by integrating measures related to use of evidence into these reports, current practice would be reviewed against the best available research and modified accordingly, more senior directors and executives would be informed about changes in practice in their areas of accountability, the importance of EBP would be emphasised throughout the organisation, and the responses could be collated to report on outcomes of the EDS. To reduce the burden on managers as much as possible, a reporting tool was drafted for inclusion in their regular monthly documentation and designed to minimise the effort required for completion (Additional file 1: Section 14).

### 1.6 Factors for success and sustainability

Prior to piloting, the characteristics, scope and components of the EDS were assessed against the criteria for success and sustainability. These were all met. Details are provided in Additional file 1: Section 7b.

### 1.7 Program logic

**Fig. 7:**
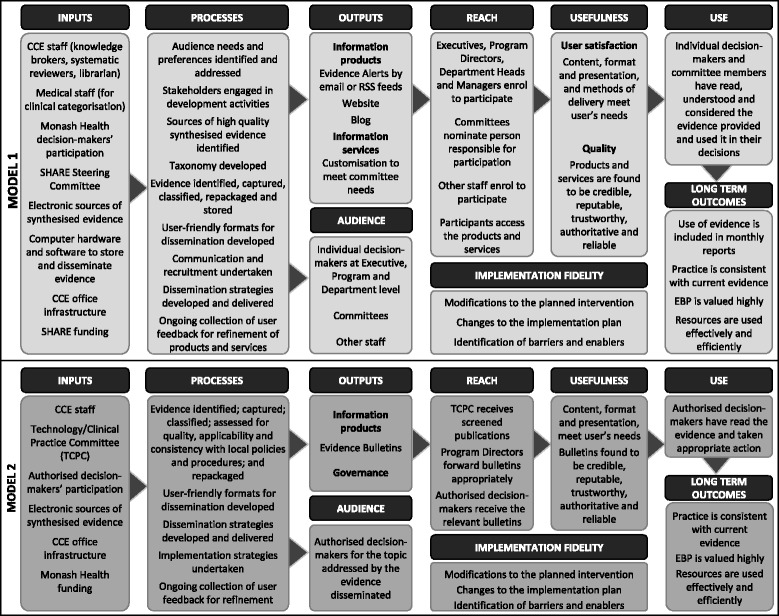
Program logic

Program logic is a systematic visual representation of the relationships between the resources available to operate the program, planned activities, anticipated results and, if a program theory was not developed, the assumptions underpinning the other elements [58]. In this paper, the assumptions are included in the program theory (Fig. 6); the traditional program logic terminology of short and medium term outcomes has been replaced with parameters recommended for evaluation of health information products and services i.e. Reach, Usefulness and Use [45]; and Implementation fidelity has been added (Fig. 7).

### 1.8 Baseline survey

All individual subscribers were invited to complete a baseline survey regarding their use of evidence when they registered with the EDS. The evaluation plan included re-administration of this survey at the end of the SHARE Program, however this was not undertaken due to the shortened timelines. The survey and results of the 46 subscribers who participated are provided in Additional file 1: Section 15.

### 1.9 Pilot

The scope, components and methods described above were piloted with a range of individual decision-makers including executives, clinical program directors and senior managers. Full details are reported in Additional file 1: Section 16.

#### Implementation

EDS staff met with committee and department representatives to seek agreement in principle and then attended meetings to explain the service and obtain agreement from individuals. Personalised emails explaining the project and requirements of participants were sent to those who were not present at the meetings. The project team enrolled each of the designated staff members, but individuals were required to register to establish their account. An email invitation with information about the EDS, an embedded link for registration, and instructions on how to activate the link was sent to each participant.

#### Evaluation

The quality, currency, content, format and methods of delivery of the EDS were all viewed positively, suggesting that methods to address the barriers, enablers and needs identified from the literature and local research were successful.

### 1.10 Revision

The factors that led to change in the processes of delivering an in-house EDS, and the resulting decisions, are reported in Additional file 1: Section 7c.

Most were minor issues in collection and processing of publications. The technical issues were addressed, a new category for ‘Disinvestment’ was introduced and participant’s responses were used to develop a FAQ (frequently asked questions) page on the website.

One noteworthy finding was that executives and senior managers reported that the information in the EDS broadcasts did not influence their decision-making because it was predominantly about clinical practice and their decisions were not. They observed that the different levels of management within the organisation required different types of information and proposed three levels: 1) Department heads and unit managers needed evidence for local policies and protocols related to clinical practice, 2) Program directors required evidence that informed their one to two year planning processes and was relevant to procedural aspects of the health service such as programs and service delivery as well as individual practitioners, 3) Executives and senior managers required information to inform three to five year forward planning that aligned with the organisation’s strategic objectives. This resulted in the addition of a category for ‘Evidence-based policy and management advice’. Potential sources were identified and, as there were no established tools to assess quality in publications of this nature, criteria were developed for this purpose (Additional file 1: Section 9).

### 1.11 Implementation

Implementation was proposed in two stages.

#### Stage 1

The model had already been piloted with individual decision-makers but was still to be tested and revised with decision-making groups such as committees. The aims were

- To implement the revised version to all staff who wished to receive EDS broadcasts
- To test the revised features with pilot committees before extending it to all decision-making groups

The Therapeutics, Medication Safety and Clinical Risk Committees were selected as a pragmatic sample of the target audience based on the potential for disinvestment in their decisions and member’s links to the SHARE Program.

#### Stage 2

The aims were

- To enrol all members of the target audience (ie all identified individuals and groups authorised to make decisions on behalf of the organisation)
- To engage the target audience in assessing current practice against evidence disseminated by the EDS, implementing change as required and reporting on the outcomes

#### Implementation strategies

Three main strategies were implemented to invite all Monash Health staff to participate in the EDS.

Communication: The EDS was launched through the Chief Executive’s newsletter, information was included in other newsletters, and flyers were distributed to physical and electronic noticeboards across the organisation.

Invitation to enrol: Information about the EDS and instructions on how to enrol were sent via the ‘All staff’ email list.

Facilitated access: ‘Hotlinks’ to the EDS were included as icons on the intranet sites of the library, pharmacy, emergency department, and medical and allied health staff portals.

Each of the selected committees nominated a liaison representative. The EDS team worked with the liaison officers to explain the process, identify barriers and enablers to using the EDS, develop methods of communication and potential strategies to use the EDS material in decision-making, and customise RSS feeds to meet their needs.

### 1.12 Evaluation

Full details of the outcomes related to Reach, Usefulness, Use, and Implementation fidelity are reported in Additional file 1: Section 17.

The survey of individual users had a 52% response rate; all health professional groups and all campuses were represented. All three committee liaison representatives and two senior individual decision-makers participated in interviews.

The quality, currency, format and methods of delivery of the EDS were all viewed positively. Most users found the content was ‘current’, ‘trustworthy’ and generally ‘useful’. Those who responded ‘partially’ or ‘no’ to some of the options explained that the information provided was not relevant to their area of clinical practice. The large volume of material disseminated was noted as a barrier to accessing the information contained in each broadcast.

Less than half of the survey respondents had used the disseminated evidence in decision-making but they were optimistic about doing so in the future. The main reasons were lack of time to read full articles and lack of relevance to their clinical setting.

Two senior decision-makers responsible for organisation-wide portfolios were consulted regarding the draft reporting tool prior to implementation in Stage 2. They were in agreement that the volume of work required to access each publication to identify whether it was relevant; then appraise it for quality, local applicability and consistency with existing policies and procedures; take appropriate action and report using the proposed tool was too onerous and it was unlikely that this model would be achievable. As a result, Stage 2 was not undertaken.

### Model 2

In this model, an Evidence Bulletin summarising a single publication was sent to the designated decision-maker authorised to make decisions for the organisation on the topic under consideration.

### 2.1 Factors influencing decisions in development of Model 2

Multiple issues were identified in the evaluation of Model 1. Their effect on the processes, outcomes and decisions related to Model 1 are provided in Additional file 1: Section 7d and summarised below.

The aim of the EDS was to ensure that organisational practice, as documented in policies and procedures, was consistent with current evidence by proactively delivering publications directly to decision-makers; and the focus of the SHARE program was to integrate new initiatives into existing infrastructure. These aims would not be met by Model 1.

While Model 1 was potentially useful for individuals to keep up with evidence in their areas of interest, given the limitation of the RSS feeds within the free software (only able to select one theme per feed), existing services from EBP and publication websites were more likely to achieve this and at no cost to the health service.

The main factors in ‘Delivery’ of the EDS fell into three groups. The first group related to governance, particularly the lack of transparency and accountability. EDS broadcasts were developed and disseminated rigorously and systematically, but were not accessed or used rigorously or systematically. Those responsible for decisions within the organisation were required to self-select and take action, but there was no process to ensure that the appropriate person with authority in the area affected by the evidence had considered the information, made a decision or taken any action. Recipients could choose whether to access, use, or report use of evidence; or not. This meant that CCE time and resources were being wasted.

The second group were methodological issues. Although the content and format of the broadcasts were well-liked by the target audience, they did not contain many of the features known to increase use and application of disseminated evidence, indicating opportunities to improve the evidence product. As noted above, the initial plan to include a statement regarding the nature of the evidence such as ‘evidence of effectiveness’, ‘evidence of harm’ or ‘lack of evidence’ was abandoned because it was frequently difficult to find such statements and, unless each article was critically appraised, we could not be confident that the findings or recommendations were trustworthy. Since the aim of the EDS was to drive decisions with proactive use of evidence, while minimising the workload of busy decision-makers, only articles containing valid evidence should be disseminated. Hence critical appraisal by the EDS team would be required.

The third group were about resources. The EDS team had difficulty processing the large number of eligible publications and proposed that the selection criteria be restricted to reduce the volume.

The main factors related to ‘Utilisation’ of the evidence were the large volume of information, large number of publications that did not require action, and lack of time to consider them. Because all newly published information from the selected sites was disseminated, findings were often irrelevant to recipient’s areas of practice, already known to them, consistent with current practice, not applicable at Monash Health, not important enough to instigate change or they reported lack of evidence. This wasted decision-maker’s time and increased the potential for them to miss relevant and significant findings. In addition, although the reporting tool was designed to minimise the effort required for completion of the tool itself, the activities to assess and apply the evidence prior to completion of the document (Fig. 5) were too onerous.

The SHARE funding was reduced in the final year of program. While this limited activities in some areas of the wider program, Monash Health provided the ongoing funding required for the EDS.

### 2.2 Potential features of an EDS in a local healthcare setting

#### Scope

The scope was revised based on the decisions in Additional file 1: Section 7d. The use of only high quality, recently published, synthesised evidence was retained from Model 1. The other parameters were replaced with the following:

To ensure that the appropriate decision-makers are engaged, that they address the evidence and take action as required, and that the process is documented and reported to ensure transparency and accountability, a governance framework would be introduced.

To reduce the amount of time spent collecting evidence, only sources that provide automated capture by email or RSS feeds would be used.

To reduce the burden on busy clinical managers, publications would be filtered before dissemination to assess lack of or inconsistency with policies and procedures, quality, applicability, and potential need for change.

To facilitate utilisation of evidence, publications would be repackaged to highlight key messages, demonstrate local relevance and implications, and provide actionable recommendations.

#### Components

The changes in scope introduced a third component of ‘Governance’ (Fig. 5). Some of the elements from the components of ‘Delivery’ and ‘Utilisation’ of evidence were re-distributed to the governance component to enable transparency and ensure accountability in organisational decision-making, to assist with filtering the large volume of information regarding local applicability and potential for change, and to identify the relevant organisational decision-maker with authority in the area addressed by the evidence.

In addition to their previous tasks, the EDS team would now also undertake ascertainment of local policies and procedures and quality appraisal of the publications.

As a result of these changes, the workload of decision-makers was significantly reduced.

#### Knowledge brokers

The same CCE expertise was involved in delivering the EDS.

#### Governing body

The Monash Health Technology/Clinical Practice Committee (TCPC) had developed an organisation-wide, transparent, accountable, evidence-based process for introduction of new TCPs [76] and had instigated the SHARE Program to take a similar approach to disinvestment. The TCPC already had the authority to require responses from organisational decision-makers and impose changes in practice related to introduction of new TCPs. Hence, it was deemed an appropriate body to undertake governance of processes to ensure that existing practice at Monash Health was consistent with the most recent evidence. The TCPC had previously included an executive sponsor; representatives with expertise in operations, finance, evidence-based practice, ethical and legal considerations; clinical program directors; health service consumers; and, when appropriate to topics under consideration, directors of pharmacy, pathology and diagnostic imaging. This was expanded for EDS governance to include all medical program directors, and senior nursing and allied health representatives.

#### Target audience

The target audience became defined by the topic of the individual publications to be disseminated: the designated individual or group authorised to make decisions related to organisational practice in the area addressed by the evidence. For example, findings related to medical treatment of diabetes would be directed to the Head of the Endocrinology Department; those related to nursing practice in childbirth would be directed to the Nurse Manager of Maternity Services; and those related to surgical consumables to the Chair of the Operating Suite Product Evaluation Committee.

#### Methods

New methods and tools for screening, appraising and reporting the quality of evidence; communicating the information to decision-makers; and capturing decision-maker’s responses were required. Most of the other methods would remain the same as in Model 1.

### 2.3 Program theory

The new influencing factors identified in evaluation of Model 1, assumptions that underpinned the choice of strategies, and strategies to deliver the intended results from Model 2 are outlined in Fig. 6.

### 2.4 Delivery of the Evidence Dissemination Service

#### Identification and capture

Publications were limited to systematic reviews, HTAs and organisational health policy documents; and sources were limited to those that provided automated capture through email broadcasts or RSS feeds.

#### Classification and storage

Publications would no longer be classified using the taxonomy. They would only be categorised based on the nature of the evidence findings e.g. evidence of harm, benefit, a more cost-effective alternative, lack of effect, and lack of evidence. No storage would be required and the EDS website was decommissioned.

#### Assessment of the evidence

One of the main changes from Model 1 was that the EDS team, rather than the decision-makers, would review local policies and procedures to ascertain whether local guidance on this topic was available and, if so, whether it was consistent with the recently published evidence. If it was, no further action would be required. If there was no local guidance, or the guidance available was inconsistent with the evidence, the publication would be appraised for quality before proceeding. Appraisal criteria and the summary table used in the new Evidence Bulletins are outlined in Additional file 1: Section 18.

#### Filtering

Publications were only considered for dissemination when the evidence was clear, the quality was high, and there was potential for change in practice at Monash Health based on lack of, or inconsistency with, local guidance.

#### Repackaging

After the TCPC determined that the evidence was applicable and there was potential for change at Monash Health (Fig. 5), the information was repackaged as an ‘Evidence Bulletin’. Bulletins were MS Word documents containing the details of a single publication and included, in order of appearance in the document, nature of the evidence (e.g. harm), topic addressed (e.g. laparoscopy for ovarian cyst), deadline for response (e.g. one month if evidence of harm), citation and hyperlink to full text, Author’s conclusions, description of Patient/Intervention/Comparator/Outcome (PICO) elements, summary of quality appraisal (quality and risk of bias of the systematic review, quality and level of evidence contained in the systematic review, and the implications of these findings), consistency with local policies and procedures, and a template for response.

Tick boxes requiring only two responses minimised the effort required of the decision-makers. The Evidence Bulletin template and an example of a completed version are provided in Additional file 1: Sections 19 and 20.

### 2.5 Governance

#### Assessment of applicability and identification of relevant decision-maker

Using their knowledge of Monash Health services, the TCPC assessed local applicability of the evidence, whether change was needed, and if so, identified the authorised organisational decision-maker. To reduce workload of the committee, screening of the publications was undertaken by the Chair prior to meetings and then provided to members at the meetings.

#### Dissemination

Each Evidence Bulletin was sent under the signature of the TCPC Chair to either the relevant Executive or Program Director, who would forward it to the decision-maker within their portfolio, or to the Chair of the relevant committee. The EDS Administrator sent the bulletins and received the responses; all correspondence was by email.

In addition, collations of bulletins that addressed topics related to diagnostic imaging, pathology, pharmacy or procurement were sent to the heads of these departments for their information; no response was required.

#### Reporting requirements

The Chief Executive determined that addressing the evidence and reporting the decisions and actions taken was a mandatory requirement of the relevant authorised decision-maker and requested monthly reports of evidence related to harm and the responses received from the target audience.

### 2.6 Utilisation of the disseminated evidence

#### Application of the evidence

The relevant decision-maker confirmed applicability and whether change was needed. They also determined whether other stakeholders should be consulted in the process, and if so, who they were. They were asked to report on their decision and, if appropriate, any action they had taken.

#### Reporting use of evidence

Responses were required within defined time frames. These were determined to prioritise action to areas of greatest risk to patients, staff or the organisation. When there was evidence of harm, a response was required within 1 month; evidence of clinical effectiveness or a more cost-effective alternative, 3 months; and lack of effect, 6 months. In the case of lack of evidence, the publication was provided for information only, no response was required. If there was evidence in more than one category, responses were requested for the one with the shortest time frame; for example evidence of harm and lack of effect in the same review would be classified primarily as evidence of harm.

Decision-makers were offered four response options, asked to tick the relevant box and then provide a brief explanation (Additional file 1: Section 20). The options were:

- Practice is consistent with the evidence
- Practice is not consistent with the evidence for a good reason
- Practice was not consistent with the evidence, remedial action has been undertaken and completed
- Practice is not consistent with the evidence and remedial action has been commenced/planned

Responses were returned to the EDS administrator.

Each month the TCPC was provided with a summary of all EDS activity and an overview of items with evidence of harm was provided to the Chief Executive. A six-monthly summary was provided to the Executive Management Team (Additional file 1: Section 21).

### 2.7 Factors for success and sustainability

Model 2 was also assessed against the criteria for success and sustainability. These were all met, however the need for adequate resources was highlighted. Details are provided in Additional file 1: Section 7b.

### 2.8 Program logic

A revised program logic for Model 2 is presented in (Fig. 7).

### 2.9 Pilot

The revised scope, components and methods described above were piloted with a pragmatic sample of publications containing evidence of harm. Full details are reported in Additional file 1: Section 22. [6]

#### Implementation

The implementation strategies focused on integrating the new processes into existing Monash Health infrastructure and communicating with stakeholders.

The procedure for the new EDS processes was documented and a routine item for discussion of EDS matters was included in the TCPC agenda.

The Director of CCE/SHARE Director made presentations to the Executive Management Team, Medical and Nursing Executive groups, and met with clinical directors of all medical programs, allied health, pharmacy, pathology, diagnostic imaging and procurement. The Chair of the TCPC delivered a presentation to the Monash Health Board. All senior managers expressed their support for the proposed governance structure. A letter outlining the new process was sent to stakeholders by the Executive Director of Medical Services and Quality and a flyer was circulated to the ‘All Staff’ email list by the Chair of the TCPC (Additional file 1: Section 23).

#### Evaluation

Six bulletins indicating harm were disseminated. They were received and returned by the appropriate decision-makers. Five responses indicated that practice was consistent with the evidence, the sixth reported that the practice was not undertaken at Monash Health. No action was required in these cases. There were no modifications to the planned intervention and it was implemented as planned.

### 2.10 Revision

The factors that led to change, and the resulting decisions, are reported in Additional file 1: Section 7e.

The main enablers were that the new EDS was promoted as an organisation-wide priority, responses were mandatory and would be audited, and all senior managers were supportive.

There were no significant barriers, but minor modifications were made to the content and format of the bulletin.

It was noted that evidence of benefit which would be of use to some decision-makers could not always be classified as clinical or cost effectiveness; for example methods to develop or implement guidelines. A new category of methodological effectiveness was added.

Drop-down boxes were introduced into the template to streamline completion by the EDS Administrator (Additional file 1: Section 19) and the table summarising quality appraisal was removed and replaced with statements regarding the appraisal findings and their implications (Additional file 1: Section 18).

### 2.11 Implementation

The scope, components, methods (with the minor revisions noted) and target audience described above formed the intervention.

No additional implementation activities were undertaken.

### 2.12 Evaluation

The EDS was discontinued prior to completion of the planned evaluation activities, however data were collected for the first seven-month period and audited to meet reporting requirements. Full details of of the outcomes related to Reach, Usefulness, Use and Implementation fidelity are reported in Additional file 1: Section 24.

During this period, 175 publications were collected and all categories of evidence were represented. Fifty-five bulletins required a response, the remainder were disseminated for information only. Forty-three responses were received at the conclusion of data collection, three had not reached their due date and nine were overdue.

Respondents reported that local practice was consistent with the evidence (*n* = 32, 74%), the evidence was not applicable at Monash Health (*n* = 6), local practice was not consistent with the evidence for a good reason (*n* = 3), and changes to make practice consistent with the evidence had been commenced or was planned (*n* = 2).

Five respondents offered positive comments, welcoming future bulletins; others suggested it was not useful to consider evidence that they were already aware of, that was consistent with current practice, or that addressed drugs that were not locally available.

One of the two departments that noted local practice was not consistent with the evidence had already *“initiated changes to current practice to conform to the recommendations”,* and the other had tasked their guideline development group to address the inconsistency.

Bulletins could also be used to confirm that current practice does not need to be changed, but the usefulness, cost-effectiveness and impact of resource use in achieving this was questioned in respondent’s feedback and project team and committee reflections.

### 3.1 Factors influencing processes and outcomes

An overview of influencing factors is presented using the framework for evaluation and explication of evidence products and services (Figs 2 and 3). Details are provided in Additional file 1: Section 7 and several factors are discussed in more detail as implications for policy, practice and research below.

The ‘External environment’ provided a wealth of high quality synthesised evidence to drive decision-making and research findings that identified desirable characteristics for evidence products and services.

The ‘Organisational environment’ was positive, the culture was supportive of change, leadership and commitment to the EDS was evident at the highest levels, the role of EBDM was valued, and proactive use of evidence to improve patient care was made an organisational priority.

There were problems with relevance of content to individuals in Model 1, but the other elements of ‘Evidence products and services’ were all highly regarded by participants in both models.

We could not establish whether the ‘Target audience’ was reached in Model 1 but the design of Model 2 enabled accurate targeting of the relevant authorised decision-maker for each publication. Decision-makers’ lack of time to deal with the multiple requirements of the EDS process led to the failure of Model 1 but this was successfully addressed in Model 2. The volume of information to each decision-maker was reduced to only a few bulletins in the seven month period, most were provided for information only, just one or two required a response. All the bulletins they received were relevant to their clinical area. This is in contrast to Model 1 where they received up to 30 per week from all clinical areas. Decision-makers’ workloads were reduced to confirming whether change was needed, taking action if required, and reporting the outcomes; which they did.

As ‘Knowledge brokers’, the CCE team had appropriate skills, relationships and credibility. The most significant barrier was resource requirements. Discontinuing categorisation by the taxonomy reduced the workload in Model 2, but expanding the activities to include assessment of consistency with local guidance and quality appraisal eliminated this benefit. Three months after implementation of Model 2, the scope was revised to focus on evidence in areas of high priority to the organisation. Publications to be appraised and disseminated with a requirement for decision-makers to respond were limited to three evidence categories: evidence of harm, which was essential for patient safety, and evidence of cost-effectiveness or lack of effect, which would complement existing Monash Health initiatives addressing organisational waste. Evidence of clinical effectiveness, methodological effectiveness and lack of evidence were provided for information only. Three months later, the EDS was suspended as CCE had insufficient resources to continue this while meeting other commitments (Additional file 1: Section 7f).

‘Processes and infrastructure’ had both strengths and weaknesses. The technical issues were minor and fixed readily. The shortcomings of the repackaging process in Model 1 were addressed in Model 2 so that only valid evidence was disseminated in bulletins that highlighted key messages, demonstrated potential inconsistency with local practice, and clearly stated required actions (Table 1). The governance elements, absent in Model 1, enabled transparency and accountability of the processes and the appropriate decision-makers received the information and responded accordingly in Model 2.

Model 2 was designed to ensure that ‘Local considerations’ were addressed.

The ‘Implementation and evaluation plans’ were achieved successfully due to provision of adequate ‘Implementation and evaluation resources’, with the exception of the final evaluation which was not undertaken due to loss of funding for the SHARE Program.

## Discussion

### Implications for policy and practice

This study provides insight into the many factors influencing the success, or otherwise, in establishing an EDS in one local health service. Issues across most of the domains of the determinants of effectiveness (Fig. 2) were addressed by the changes made in Model 2. However there are remaining issues in two domains that require consideration for future implementation of an in-house EDS.

#### Process and infrastructure

**Fig. 8:**
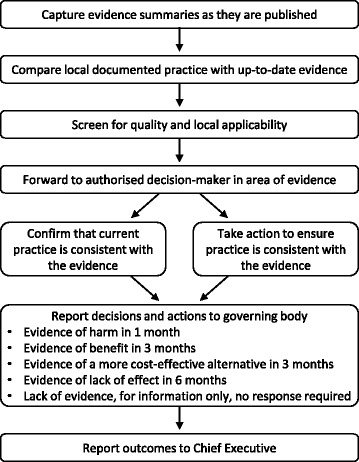
Flow chart of EDS Model 2 process

Several respondents appeared to be unclear about the purpose of the EDS, in particular it was perceived that CCE had undertaken the reviews, rather than capturing synthesised evidence as it was published by others. This understandably led to questions about why some topics had been selected, particularly if they were not locally applicable. The process had been explained in correspondence during the implementation phase (Additional file 1: Section 23), but if decision-makers had not read or remembered this information, there was nothing in the Evidence Bulletin to explain the process. A flowchart (Fig. 8) or text summary of the process within each bulletin may address this.

Monash Health is an academic health network providing a range of services from primary to quaternary programs. Several respondents pointed out that they had been involved in undertaking systematic reviews and participating in national and international guideline development in their areas of expertise and were therefore aware of the current evidence and responding to the bulletin was wasting their time. This is a valid criticism that identifies potential differences in need between highly-specialised academic facilities and more general health services, or between individual units within a single facility. However, while individuals may be aware of current evidence in areas they have reviewed, they may not be familiar with the most recent evidence in other areas of their speciality. The experience of the CCE team, who delivered regular workshops on finding the best available evidence, was that very knowledgeable clinicians thought that they were abreast of up-to-date information based on reading the main journals in their clinical areas. However many publications of synthesised evidence are distributed through different channels and, when new information was identified in the CCE workshops, it frequently contradicted clinicians’ previous understanding of the current evidence. A systematic approach to dissemination of evidence is unlikely to be able to identify when a decision-maker is aware of current information and when they are not. This is a barrier which may result in loss of support from stakeholders who are unhappy to have their practice questioned or to spend time addressing something that they know is not a problem. Clarifying the process within each bulletin may also help to alleviate this.

Even with several filtering steps, topics that were not applicable in the local setting were still disseminated. Some bulletins contained information about drugs that are not available in Australia; identifying and removing these would be straightforward, but would require additional resources for the EDS team. Identifying and removing all practices that are not undertaken locally may be less straightforward since the topics found not to be applicable had been vetted by senior staff and directors of the relevant clinical programs; it may not be possible for them to be familiar with every practice in their portfolios.

#### Knowledge brokering

The characteristics of the studies included in the publications such as setting, population/patients, intervention, control/comparator, outcomes and selection criteria, were extracted and summarised in the bulletin. Some respondents noted that they needed additional information, such as more details of the intervention and statistical and clinical significance of the results, in order to make a decision. This would require involvement of clinicians and/or more senior evidence consultants than the EDS model trialled, and would transfer the clinical assessment from the designated decision-maker, who was likely to be the most senior practitioner in the relevant specialty, to someone less qualified and experienced. If the information is available in the publication it could be incorporated into the evidence classification, for example *“Evidence of effectiveness but of uncertain clinical significance”.*

There may be better ways of dealing with some complex issues than dissemination of individual bulletins. Three reviews of wound dressings were captured in one month, and a different decision-maker was initially allocated to each one. Shortly afterwards, a review of blunt versus sharp suture needles for preventing needle stick injuries was published. It was obvious that a single person was not responsible for decisions in these areas. Monash Health policies and procedures had insufficient documentation to know whether current practice was consistent with the evidence. Based on the SEAchange model for evidence-based change [39], a ‘project approach’ was proposed that involved ascertaining additional information and consulting with stakeholders before determining the next stage. This process was begun but not completed due to the suspension of the EDS. The protocol is provided in Additional file 1: Section 25.

The largest barrier to delivery of an in-house EDS was insufficient resources. It is also clear that delivery of an EDS at the local healthcare level is potentially a significant waste of resources if it is being duplicated in multiple facilities. High quality synthesised information is being produced by multiple publishers with no single point of access from which to generate proactive capture to drive decision-making. The Cochrane Library has partially addressed this by bringing together their own systematic reviews with some reviews and HTAs from other sources, but there are still many reviews and HTAs omitted and evidence-based guidelines are not included [77]. John Lavis notes that our future challenges include *“examining whether and when any apparent duplication of efforts occurs in the production of review-derived products at the international level; and scaling up activities that are found to be effective in supporting the use of reviews and review-derived products in policymaking”* [29].

### Implications for research

Many publications had more than one conclusion: for example harm plus effect or effect plus lack of evidence. New methods are needed to address this in the dissemination and reporting processes.

The original aim of the EDS also included dissemination of evidence-based guidelines. While the capture and processing of guidelines would be mostly the same as systematic reviews and HTAs, the multiple recommendations made dissemination difficult; exploration of this was not undertaken due to suspension of the service. Investigation of methods to disseminate evidence in these situations is warranted.

The governance approach utilised in Model 2 could be classified as a *“quality focused initiative*” from the review by Hastings and colleagues [78]. There are six types of governance mechanisms proposed in this review which could be explored for future implementation of an EDS.

The framework for evaluation and explication of implementation of evidence products and services requires further testing and revision. The elements were chosen pragmatically to suit the circumstances of the Monash Health EDS and there are some potential overlaps in domains.

### Contribution of this study

This study provides the details of a systematic process for recently published, high quality, synthesised evidence to be *“captured from outside, circulated internally, adapted, reframed, implemented, and routinized in a service organization”* [33]. To our knowledge, this is the only report of development, implementation and evaluation of an in-house EDS implemented in a governance framework within a local healthcare setting.

Existing evidence services deliver bulletins on selected topics to individual subscribers, such as McMaster Evidence Alerts, Clinical Evidence and Evidence Updates [79–81]. Types of evidence products have also been defined, for example Lavis’s categories of *“(1) summaries of systematic reviews highlighting decision-relevant information; (2) overviews of systematic reviews providing a “map” of the policy questions addressed by systematic reviews and the insights derived from them; and (3) policy briefs drawing on many systematic reviews to characterize a problem, policy or program options to address the problem, and implementation strategies”* [29]. There are many similarities between these examples and the SHARE EDS; Model 1 is comparable to the evidence alert services and Model 2 has elements of all the evidence products. However there are several key differences between the models explored here and those trialled by others.

The main distinctions are related to the in-house systematic approach to using evidence proactively to ensure organisational practice is consistent with current evidence.

Many studies have explored the characteristics and use of publications as evidence products [25–29, 50, 55, 63–67, 82]. In addition to content and format of the products, others have noted the need to target individual decision-makers [25, 27, 29] who are authorised to implement change [9, 14, 83–87] with timely [34, 48] and locally relevant information [29, 64, 66]; actively deliver the evidence directly to decision-makers [25, 34, 82]; create an organisational culture supportive of EBDM [25, 29]; make use of existing formal infrastructure [14, 16, 34, 71] in a governance framework to provide legitimacy and engagement [88] particularly in the case of disinvestment where a governance committee is thought to “*make contentious decisions more palatable and defensible*” [19, 89–91]; and clearly identify requirements for accountability [26, 50, 83, 88] including mandated responses [30] and use of reporting tools [88].

The EDS Model 2 may be the first to integrate all of these. It builds on earlier findings by focusing on new organisation-wide systems and processes embedded in existing infrastructure, such as CCE, TCPC, authorised decision-makers, and reporting networks, in which to disseminate evidence within a governance framework.

The Evidence Bulletins had elements of each of Lavis’s categories – summaries, overviews and policy briefs – but they also had critical differences with other disseminated evidence products.

- The nature of the evidence, such as evidence of harm, clinical or cost-effectiveness, lack of effect, or lack of evidence, was defined for each publication and used to determine the next steps for knowledge brokers and decision-makers.
- Each article was critically appraised for quality and an appraisal summary including implications was provided for the reader; low quality reviews were not disseminated.
- Local implications were considered.
  —Publications were only disseminated if they were inconsistent with organisational policies and protocols or there was no relevant local guidance on this topic.
  —Applicability was assessed by senior managers prior to dissemination and PICO characteristics were extracted and summarised to enable the authorised decision-maker to confirm local applicability.
- Specific time-critical actions were required of the recipients; for example in the case of evidence of harm, decision-makers had to determine whether practice change was required, develop a plan for action, and respond with the details within one month.

The governance elements ensured transparency through clear systems and processes and accountability through reporting requirements. The EDS was given high priority by the Chief Executive who instigated the mandatory responses and implementation was integrated into the organisational Business Plan.

### Limitations

The EDS was implemented in an Australian public health service where all staff are bound by organisational policies and procedures; this may limit the generalisability to other settings.

The SHARE Program was primarily a health service improvement initiative rather than a research project, however an explicit research framework was included in its development [44]. The project team responsible for delivering the EDS at Monash Health were also the researchers investigating the processes undertaken. This has the potential to introduce subjectivity into evaluations and limit insight if assumptions are accepted without challenge. Detailed exploration and documentation of ‘learnings’ throughout the project, extensive stakeholder involvement, transparency of methods and participation of an external evaluator in the role of ‘critical friend’ [44] were included in the SHARE processes to minimise these limitations.

The level of expertise within the Centre for Clinical Effectiveness is unusual in this context and will limit generalisability of the models presented to other settings. Although hospital-based resources for knowledge brokering are becoming more common [92, 93], they are not widespread, and the additional skills in implementation and evaluation are less common.

Model 2 achieved its aims, however delivery was restricted to evidence of harm and cost-effectiveness resulting in limited impact; only two bulletins initiated practice change. This process ensured that only high quality evidence was used to drive decisions, but it excluded potentially high quality information from other sources such as journals and peak body websites. It is likely that if eligibility of sources or individual publications was not restricted there would have been a greater impact. However, the greater impact may not only effect organisational practice, but also the workloads of decision-makers and knowledge brokers and require additional resources.

The reduced funding and lack of capacity imposed some limitations in implementation and evaluation of the EDS. As these are not uncommon occurrences in health service initiatives, reflecting real as well as hypothetical limitations, they need to be considered in future planning for in-house services.

The reduction of funding, followed by suspension of the service, meant that the planned evaluation was not undertaken. Although the audit was based on small numbers and some self-reported responses were not verified, it provides useful information for future planning.

## Conclusion

An in-house EDS holds promise as a method of identifying disinvestment opportunities and/or ensuring practice in a local healthcare service is consistent with current evidence. The resource-intensive nature of delivery of the EDS is a potential barrier. The findings from this study will inform further exploration.

## Additional file

Additional file 1: Methods and Results. (PDF 2081 kb)

## Abbreviations

AGREE: Appraisal of Guidelines for Research and Evaluation
CCE: Centre for Clinical Effectiveness
EBDM: Evidence-based decision-making
EBP: Evidence-based practice
EDS: Evidence Dissemination Service
FAQ: Frequently asked questions
HTA: Health Technology Assessment
ICD-10-AM: International Statistical Classification of Diseases and Related Health Problems, Tenth Revision, Australian Modification
MeSH: Medicine Medical Subject Headings
MRC: Medical Research Council
RSS: Really Simple Syndication
SHARE: Sustainability in Health care by Allocating Resources Effectively
SR: Systematic Review
TCPC: Technology/Clinical Practice Committee
TCPs: Technologies and clinical practices
