# Supplementary material for: Sustainability in Health care by Allocating Resources Effectively (SHARE) 8: developing, implementing and evaluating an evidence dissemination service in a local healthcare setting
Source: BMC Health Serv Res. 2018 Mar 2;18:151. doi: 10.1186/s12913-018-2932-1 (PMC5833068; doi:10.1186/s12913-018-2932-1)
Supplement: Supplementary file 1 — Methods and Results. (PDF 2081 kb) [file 12913_2018_2932_MOESM1_ESM.pdf]

Sustainability in Health care by Allocating Resources Effectively (SHARE) 8: Developing, implementing and evaluating an Evidence Dissemination Service in a local healthcare setting

Additional File: Methods and Results

Contents

Section 1 Identifying the need for change: Data collection methods and sources 2

Section 2 Developing the intervention: Feedback, refinement and decision-making 4

Section 3 Evaluating the change: Evaluation Plans 5

a. Model 1 5

b. Model 2 6

Section 4 Survey of decision-makers: Preferred content and format of evidence product 7

Section 5 Factors that influenced organisational decision-making relevant to EDS 8

Section 6 Factors that influenced development of SHARE Program relevant to EDS 16

Section 7 Factors that influenced development, processes, outcomes and revision of EDS 18

a. Development 18

b. Success and sustainability 20

c. Model 1 Pilot 22

d. Model 1 Full implementation 23

e. Model 2 Pilot 24

f. Model 2 Full implementation 24

Section 8 Options considered in development of EDS 25

Section 9 Definitions of evidence products, inclusion criteria and appraisal applied to publication sources 26

Section 10 Sources of synthesised evidence 28

Section 11 Taxonomy 29

Section 12 Model 1 Examples of the EDS Website 32

Section 13 Model 1 Example of EDS Email Alert 35

Section 14 Draft tool for reporting use of evidence with completed examples 36

Section 15 Survey of staff enrolling in the EDS: Baseline data 37

Section 16 Model 1 Pilot implementation and evaluation 40

Section 17 Model 1 Evaluation of full implementation 45

Section 18 Systematic Review Appraisal 52

Section 19 Model 2 Evidence Bulletin template 54

Section 20 Model 2 Example of an Evidence Bulletin 56

Section 21 Model 2 Report to Executive Management Team 58

Section 22 Model 2 Pilot implementation and evaluation 61

Section 23 Model 2 Implementation flyer 62

Section 24 Model 2 Evaluation of full implementation 63

Section 25 Protocol to address evidence findings involving multiple decision-makers 66

Section 26 References 73

Section 1 Identifying the need for change: Data collection methods and sources

LITERATURE REVIEW

Information needs of decision-makers

Aim: To identify the information needs of decision-makers in local healthcare services to facilitate development of pilot support services.

Questions: What are the information needs of clinicians and managers to support evidence-based decision-making regarding the introduction or removal of technologies and clinical procedures?

How have assessments to determine these needs been conducted in the past?

Sources: Medline, CINAHL, EMBASE, LISA, LISTA and Google

Medline Search (adapted for other databases): (exp Needs Assessment/) AND (Information Dissemination/ or Information Services/ or Information Management/) limit to (English language and humans)
Google Search: (information OR evidence) AND (need OR assessment) AND (health OR nurs OR doctor OR med). Preferences were set to English language.

Inclusion criteria: Articles describing information needs assessments in similar health service contexts examining how clinicians and managers make evidence-based decisions regarding the introduction or removal of technologies and clinical practices; articles published in English from 1996.

Exclusion criteria: Information needs of students; continuing professional education needs; point of care decision-making needs; assessments of information needs in resource poor health settings

Data Collection and Analysis: Inclusion, exclusion and appraisal criteria were established a priori. Studies to be reviewed by one reviewer in consultation with colleagues when necessary. Critical appraisal relevant to study design to be conducted using standard CCE templates.

Results: No studies were found to meet the inclusion criteria. The limitations of the very specific question and narrow selection criteria were acknowledged. Earlier broad searches resulted in unmanageable numbers of returned articles, however limiting the search returned none. Since the purpose of the review was to inform development of the support services, and not to be a systematic review providing a definitive answer for others, a decision was made to take a pragmatic, iterative approach by accessing relevant publications already known to the project team and following up with simpler searches, pursuing articles from reference lists, etc.

SURVEYS

Staff who made decisions about resource allocation

Aim: To identify the information needs of decision-makers at Monash Health to facilitate development of support services and gather baseline data for evaluation purposes.

Participants: Staff who made decisions regarding resource allocation for technologies and clinical practices.

Design and content: An electronic questionnaire was designed and delivered using SurveyMonkey [1]. Questions were developed to identify current use of evidence; confidence in searching for, accessing and appraising evidence; difficulties in using evidence and implementing evidence-based change; preferred content and format of bulletins disseminating research evidence; and preferred formats for education and training in these areas. Some questions were adapted from Taylor et al [2].

Pretesting and piloting: The survey was pre-tested with colleagues at a co-located research institute, piloted with the SHARE Steering Committee, and refined based on feedback from these groups

Distribution: An email with an embedded link to the survey was distributed to senior staff using the Monash Health 'All Managers' and 'Senior Medical Staff' email lists. Members of these lists were asked to forward the survey to others who made decisions about resource allocation but might not be on the list.

Data collection: Data were collected over a four-week period from the time of distribution. No reminders were sent.

Analysis: Results were downloaded into Excel from the survey provider. Qualitative data from the three free text answers were copied into NVivo [3] where they were coded according to themes presented in Michie et al [4]. Data were reviewed by two investigators to ensure agreement of coding. Discrepancies were discussed with a third investigator until consensus was reached. There were insufficient categories in the Michie et al framework to address some of the organisational issues; additional themes were created as required.

Response rate: 141 staff members responded. 118 were eligible to complete the survey having answered 'yes' to the screening question asking if they made decisions about resource allocation. 103 completed the entire survey. The response rate could not be calculated in the absence of denominator information; the total number of staff on the email lists and the number of additional staff to whom the survey was forwarded were unknown.

Representativeness of sample: All programs and service sites were represented in proportions consistent with the size of the program or campus. A range of professional disciplines were represented: nursing (28%), allied health (25%), medical (24%) and other (23%) including pharmacy, diagnostic services, corporate and clinical program management, and administration.

**Staff enrolling in the Evidence Dissemination Service (Baseline survey)**<br>**Aim:** To ascertain how participants enrolling in an Evidence Dissemination Service (EDS) currently use evidence in decision-making.<br>**Participants:** Staff members enrolling to participate in EDS.<br>**Design and content:** An electronic questionnaire was designed and delivered using SurveyMonkey [1]. Questions were developed to identify current use of evidence; time spent searching for, accessing and appraising evidence; perceptions of EBP at Monash Health and features of respondent's decision-making practice.<br>**Distribution and Data collection:** The survey was part of the enrolment process.<br>**Analysis:** Results were downloaded into Excel from the survey provider.<br>**Response rate:** 46 staff members enrolled to participate in EDS during the survey period.<br>**Representativeness of sample:** Respondents represented all clinical groups and all health service programs and sites.
**INTERVIEWS**
**Members of organisation-wide committees, representatives of approved purchasing units and individuals who made decisions about resource allocation**<br>**Aim:** 1) To examine and document current processes for making, implementing and evaluating decisions and the factors that influence them (all interviewees) and 2) To identify relevant issues and pilot draft questions for needs analysis survey (clinical program managers only).<br>**Participants:** Invitations were extended to 1) representatives of 14 committees with a mandate to make organisation-wide decisions regarding allocation of resources, 2) managers of 5 approved purchasing units (APUs) and 3) 9 managers from one clinical program selected for its high use of health technologies.<br>**Interview schedule:** Questions were designed to identify how evidence and data were used in decision-making, implementation and evaluation and the associated barriers and enablers (all interviewees). Additional questions were designed to identify training and support needs for decision-making, implementation and evaluation and preferred formats for delivery (clinical program managers only). These were part of a schedule investigating organisational decision-making more broadly. The full interview schedule is available [5].<br>**Data collection:** Interviews were approximately 1 hour long. Two CCE staff members attended, one as facilitator, one as note taker. Drafts were sent to the interviewees for clarification, comment and/or amendment as required.<br>**Analysis:** Final interview notes were analysed thematically in MS Word and Excel using the elements of the theoretical framework.<br>**Response rate:** 13 of the 14 committees, all 5 APU managers and all 9 clinical managers participated<br>**Representativeness of sample:** All but one of the relevant committees and all APUs were represented, the clinical managers selected represented Program Directors, Medical Department Heads, Nurse Unit Managers and Quality and Risk Manager in medical and surgical sub-specialties, nursing and quality management across a range of campuses.
**WORKSHOP**
**Structured workshop with decision-makers from a large diagnostic service**<br>**Aim:** To capture the process of capital equipment purchasing in a large multi-campus diagnostic service and how an ideal process for this decision-making might differ from the current process.<br>**Participants:** The Director and Research Director of the diagnostic service generated the invitation list. Eighteen decision makers from all units, campuses and health professional groups within the service were invited by the Executive Director Medical Services and Quality.<br>**Design:** An experienced facilitator from CCE who had no involvement in the SHARE project developed and delivered the workshop. A presentation on the background of the project and its relevance to the workshop was made by a SHARE project team member. Two other project team members were present to assist with logistics and note taking. The session was run over 1½ hours in the departmental seminar room. Five domains were identified a priori: how do we get an idea; what is the process (application, approval, feedback, who, timing); is it a good idea; is it the best idea and monitoring and evaluation. Barriers and enablers were explored.<br>**Data collection:** Using a nominal group technique, participants were asked to describe the ideal process for purchasing large capital equipment. Responses were collected on sticky-notes. This method was repeated to identify gaps in the current process and included prioritisation of key areas for improvement.<br>**Analysis:** Responses on the sticky notes were collated under the domains identified a priori. They were analysed within these domains to identify key themes.<br>**Response rate:** 17 of the 18 invitees attended. An additional staff member from a clinical area not represented on the invitation list was included at the commencement of the workshop.<br>**Representativeness of sample:** A range of medical, nursing, technical, quality improvement staff and business management representing all units within the department and all campuses attended.

Section 2 Developing the intervention: Feedback, refinement and decision-making

INDIVIDUAL AND GROUP DISCUSSIONS

Senior decision-makers

Aims: To inform senior decision-makers of proposed plans, ascertain feedback regarding feasibility and acceptability, and seek support and endorsement.

Participants: Nursing Executive Team, all Medical Program Directors and the General Manager of Allied Health.

Format: Nursing Executive Team met as a group, Program Directors and General Manager were consulted individually. A summary of the proposed EDS was presented and participants asked for their feedback.

Data collection: Discussions were approximately 30 minutes long. The CCE Director/SHARE Program Director was the presenter and facilitator. Notes were taken.

WORKSHOPS

Aims: To review and refine draft proposals and make final decisions.

Participants: Initially held with the EDS Advisory Group, including an Executive Director (Nursing), General Manager (Allied Health) and two Department Heads (Surgery and Information Technology). Subsequently held with the SHARE Steering Committee including Executive Directors (Medical, Nursing, Support Services), Program Directors (Medical, Nursing, Allied Health, Pharmacy, Diagnostic Services), Committee chairs (Technology/Clinical Practice, Therapeutics, Human Research and Ethics, Clinical Ethics), Managers (Information Services, Clinical Information Services, Procurement, Biomedical Engineering, Research Services), Legal counsel and two Consumer representatives.

Design

- Provision of pre-reading materials and/or workshop presentation of background, issues to consider, draft proposals, etc
- Agenda including points for discussion and decisions required
- Documentation of discussion, decisions and actions in minutes

Structured decision-making workshops were held at scheduled meetings of both groups. Discussion papers and background documents were provided beforehand, formal presentations introduced the workshops, and topics for discussion and decisions required were listed on the agenda.

Deliberation

The deliberative process was informal within the structure of the agenda and decisions were based on consensus. Discussion, decisions and actions were documented in minutes which were confirmed at subsequent meetings.

FACTORS FOR SUCCESS AND SUSTAINABILITY (Reproduced from Harris et al [6] with permission)

Success

A proposal is more likely to be successfully implemented if it meets the following criteria:

- It is based on sound evidence or expert consensus
- It is presented by a credible organisation
- It can be tested and adapted
- The relative advantage is evident
- It is of low complexity
- It is compatible with the status quo
- It has an attractive and accessible format

Sustainability

A proposal is more likely to be sustainable if it has appropriate and adequate provision in each of the following categories:

- Structure
- Skills
- Resources
- Commitment
- Leadership

Section 3 Evaluating the change: Evaluation Plans

a. Model 1

Domains	Key Evaluation Questions	Data Sources	Data collection methods	Outcomes
Reach	Have Southern Health Decision-makers either personally reviewed or nominated a member to receive and report EDS alerts?	Decision-maker documentation	Document analysis	Committees, Departmental, Executive and Program heads sign up to EDS or nominate an employee to receive alerts relevant to their area of specialty.
	What are the trends in Southern Health User enrolment for EDS alerts?	EDS Web-based statistics	Audit	
	How often are Southern Health staff accessing the EDS website?	EDS Web-based statistics	Audit	
Usefulness	Which aspects of EDS presentation, content and format do decision-makers find helpful?	Decision-makers	Interviews and surveys	Decision-makers review relevant information from EDS alert and retrieve full text where necessary<br>Southern Health decision-making Committees discuss relevant information identified in EDS alert
		Users	Surveys	
	Which aspects of EDS presentation, content and format do decision-makers feel could be improved?	Decision-makers	Interviews and surveys	
		Users	Surveys	
	Do decision-makers consider information delivered by EDS as being credible, reputable, authoritative, and trustworthy?	Decision-makers	Interviews and surveys	
		Users	Surveys	
Use	Have EDS Alerts been discussed in Southern Health decision-making committee meetings?	Decision-maker Committee documentation	Document analysis	Decision-making Committees, Executive, Department heads and Program heads respond to relevant information identified in EDS alerts and Southern Health TCPs are adapted accordingly
	Have Committees, Executives, Program heads and Department heads used information received from EDS to guide decision-making?	Decision-makers	Interviews and surveys	
	Do Committees, Executives, Program heads and Department heads intend on using information received from EDS in future decision-making?	Decision-makers	Interviews and surveys	
	Is there evidence that EDS has been used to inform disinvestment activities?	Decision-makers	Interviews and surveys	
	Is there evidence that TCP related decisions have been made without input from Committees? (related to wider implementation with staff)	Users	Surveys	
Implementation	To what extent has EDS been implemented as planned?	EDS team	Group interview	EDS is fully implemented
	What do decision-makers report to be the barriers and enablers of implementing evidence received from EDS into practice?	EDS team	Group interview	
	Were there any gaps in the implementation of EDS that need addressing to meet program aims?	EDS team	Group interview	

b. Model 2

Domains	Evaluation Questions	Data Sources	Data collection methods	Outcomes
Reach	How many evidence bulletins have been disseminated through the TCPC to decision-makers?	EDS Database	Audit	The EDS appraises the quality and relevance of evidence prior to disseminating to the appropriate stakeholders at Southern Health
	How many evidence bulletins had related Southern Health policies, procedures or guidelines?	EDS Database	Audit	
	How many evidence bulletins were inconsistent with local policies and procedures?	EDS Database	Audit	
	How many pieces of evidence required action by the decision-maker and/or stakeholders?	EDS Database	Audit	
Usefulness	Was the TCPC satisfied with the format and presentation of the evidence?	TCPC	Interviews	Decision-makers find the content, presentation and delivery of the new process for disseminating evidence useful
	Were decision-makers satisfied with the new EDS/TCPC process?	Decision-makers	Survey	
	Were decision-makers satisfied with the format and presentation of the evidence?	Decision-makers	Survey	
	Do decision-makers consider the content of the evidence bulletins to be useful?	Decision-makers	Survey	
	What aspects of the format, presentation and content could be improved?	Decision-makers	Survey	
	Was the TCPC satisfied with the format and presentation of the templates for reporting?	TCPC	Interviews	
	Were decision-makers satisfied with the format and presentation of the templates for reporting?	Decision-makers	Survey	
	Do decision-makers consider information delivered by the new EDS to be credible, reputable, authoritative, and trustworthy?	Decision-makers	Survey	Decision-makers find the evidence delivered to them to be credible, reputable, authoritative, & trustworthy
Use	How many evidence bulletins identified by the EDS were shown to have evidence of harm, evidence of benefit, evidence of a more cost-effective alternative, or evidence of lack of effect?	EDS Database	Audit	Decision-makers use the evidence presented to them to inform or change current practice
	How many evidence bulletins identified by the EDS required action by the decision-maker and/or stakeholders due to inconsistency with Southern Health policies, procedures or guidelines?	EDS Database<br>EDS Reporting Database	Audit	
	How many decision-makers report that their practice would require a change based on the evidence presented?	EDS Reporting Database	Audit	
		Decision-makers	Survey	
	How many decision-makers actually changed their practice based on the evidence presented?	EDS Reporting Database	Audit	
	What types of change were involved?	Decision-makers	Survey	
	Have all instances of evidence of harm been forwarded to the Executive Management Team?	EDS Database	Audit	Procedures where there is evidence of harm are not undertaken
	How many decision-makers reported to the TCPC within the appropriate period of time?	EDS Reporting Database	Audit	Decision-makers respond to presented evidence in the appropriate timeframe
		TCPC	Survey	
Implementation	To what extent has the new EDS been implemented as planned?	EDS Team & TCPC	Discussion/reflection	Southern Health practice is consistent with current high-quality synthesised evidence
	What do decision-makers report to be the barriers and enablers of implementing evidence received from the new configuration of EDS into practice?	Decision-makers	Survey	
	Were there any gaps in the implementation of the new EDS that need addressing to meet program aims?	EDS Team & TCPC	Discussion/reflection	

Section 4 Survey of decision-makers: Preferred content and format of evidence product

From survey of Monash Health staff who made decisions about resource allocation.

Full details of all survey questions are in Paper 7 of this series [7].

Type of research publication to inform decisions about health technologies or clinical practices

Respondents were invited to choose as many as applied	**n (%)**
Critical appraisals of primary research	88 (83.0)
Full text of secondary research (eg evidence-based guidelines, systematic reviews)	83 (78.3)
Critical appraisals of secondary research	79 (74.9)
Full text of primary research (eg clinical trials)	73 (68.9)
Abstracts of primary research	50 (47.2)
Abstracts of secondary research	44 (41.5)
Other*	7 (6.6)
Total	106
*Other: consumer perspectives, case-studies of other health services, web-access to journals, professional guidelines and web-access for participation in group wide trials	

Focus of research to inform decisions about health technologies or clinical practices

Respondents were asked to rank at least three preferences with 1 being the most preferred option	**1** n (%)	**2** n (%)	**3** n (%)	**4** n (%)	**5** n (%)	**6** n (%)
Condition specific information (eg Diabetes)	25 (23.8)	26 (25.2)	18 (17.5)	7 (13.0)	8 (20.0)	3 (21.4)
Professional group information (eg Emergency Department Nursing)	23 (21.9)	25 (24.3)	17 (16.5)	8 (14.8)	6 (15.0)	0 (0.0)
Program relevant information (eg Mental Health)	21 (20.0)	20 (19.4)	26 (25.2)	16 (29.6)	2 (5.0)	0 (0.0)
Organisation wide information (eg Infection Control)	15 (14.3)	14 (13.6)	15 (14.6)	14 (25.9)	16 (40.0)	1 (7.1)
Unit relevant information (eg Monash Newborn Services)	13 (12.4)	18 (17.5)	26 (25.2)	9 (16.7)	8 (20.0)	2 (14.3)
Other*	8 (7.6)	0 (0.0)	1 (0.97)	0 (0.0)	0 (0.0)	8 (57.1)
Total	105	103	103	54	40	14
*Other: consumer initiated, focused and developed research; international relevance; focus needed depends on the task; skill or procedure specific eg bed management						

Format of research dissemination to inform decisions about health technologies or clinical practices

Respondents were asked to rank at least three preferences with 1 being the most preferred option	**1** n (%)	**2** n (%)	**3** n (%)	**4** n (%)	**5** n (%)	**6** n (%)	**7** n (%)
Short pdf attachment to an email (eg titles and hyperlinks)	33 (32.4)	19 (18.8)	26 (25.5)	5 (11.9)	0 (0.0)	0 (0.0)	0 (0.0)
Long pdf attachment to email (eg titles, abstracts, hyperlinks)	26 (25.5)	22 (21.8)	11 (10.8)	8 (19.0)	2 (6.3)	3 (10.7)	0 (0.0)
Email with titles and embedded hyperlinks	18 (17.6)	26 (25.7)	21 (20.6)	2 (4.8)	7 (21.9)	4 (14.3)	0 (0.0)
Searchable database	18 (17.6)	13 (12.9)	19 (18.6)	12 (28.6)	6 (18.8)	7 (25.0)	1 (7.7)
Short paper-based newsletter (eg titles and web addresses)	4 (3.9)	14 (13.9)	13 (12.7)	9 (21.4)	6 (18.8)	5 (17.9)	1 (7.7)
Long paper-based newsletter (eg titles, abstracts, web addresses)	2 (1.9)	6 (5.9)	9 (8.9)	6 (14.3)	11 (34.4)	12 (42.3)	4 (30.8)
Other*	1 (1.0)	1 (1.0)	3 (2.9)	0 (0.0)	0 (0.0)	0 (0.0)	7 (53.9)
Total	102	101	102	42	32	28	13
*Other: short summaries about the article and main findings and then a link to the full article; lectures and/or in-services; website; full text review articles by well-respected authors; workshops regarding methods eg statistics, database development							

Section 5 Factors that influenced organisational decision-making relevant to EDS

Reproduced from Harris et al [5] with permission.

Items related to proactive use of evidence in decision-making are highlighted. The other items are retained to provide context.

Factors identified in response to a specific question about barriers and enablers are noted in italics.

STRENGTHS/ENABLERS	WEAKNESSES/BARRIERS
**External environment**	
**General**<br>*Good relationships with external agencies such as Australian Council of Healthcare Standards, Victorian Department of Human Services (DHS)*<br>*Projects initiated by external organisations such as Australian Quality Council, NSW Therapeutics Advisory Group and Clinical Excellence Commission*	
▪ Legislation, regulations, national and international standards, and professional standards must be followed. This provides clarity and certainty for some decisions.	▪ Some decision-makers are unaware of mandatory requirements.
**International**<br>▪ International bodies and national agencies of other countries provide evidence-based recommendations for use of health technologies, clinical practices, models of care, etc.<br>▪ Systematic reviews and Health Technology Assessments are also available.	▪ Decision-makers are frequently unaware of these resources.<br>▪ Due to lack of time, knowledge and skills decision-makers do not actively seek these resources when making decisions and do not differentiate between high and low quality resources.<br>▪ Cost-effectiveness data is often based on modelling which is perceived not to reflect reality
**National**<br>▪ The Medical Services Advisory Committee and Pharmaceutical Benefits Advisory Committee provide evidence-based recommendations for use of medical and surgical procedures and drugs.	▪ Not all medical and surgical procedures and drugs are covered by these processes.<br>▪ Nursing and allied health practices, models of care and clinical consumables are not covered.
**State**<br>▪ Guidance for introduction of new health technologies and clinical practices (TCPs) is provided by DHS. This includes reporting requirements.<br>▪ Monash Health has developed tools to implement these processes. DHS has recommended these tools to other health services.<br>▪ Monash Health Decision Summaries are published on the health service website.	*DHS requirements and processes are cumbersome*<br>▪ There is no sharing of information or decisions. Individual health services duplicate the process of finding and appraising relevant evidence, developing business cases, etc.<br>▪ DHS declined to coordinate sharing of information through a central database or website.
▪ The Victorian Policy Advisory Committee on Technology (VPACT) has an annual funding round for introduction of new high cost TCPs	▪ Respondents unaware of any long-term state-wide strategic planning for equipment purchases<br>▪ Lack of coordination of equipment use and procurement at state level and no communication between health networks.
▪ Some guidance for purchasing is provided through the Victorian Government Purchasing Guidelines, Medical Equipment Asset Management Framework (MEAMF), Targeted Equipment Replacement Program (TERP) and Health Purchasing Victoria (HPV).<br>▪ HPV is responsible for bulk purchasing of pharmaceuticals, clinical equipment and consumables to streamline ordering and reduce costs. If the item required is in the HPV catalogue the specified brand must be purchased from the designated suppliers at the cost and conditions noted.<br>▪ The processes are transparent and accountability is clear.	▪ HPV catalogue only covers 30% of Monash Health consumables<br>▪ Inclusion of items in the HPV catalogue is not always based on a rigorous evidence-based process<br>▪ Safer, more effective or more cost-effective alternatives may not be included in the catalogue<br>▪ HPV does not cover large items so MEAMF and TERP have no benefits from bulk purchasing and hospitals have to negotiate their own arrangements with suppliers<br>▪ Decision-makers do not know which of these multiple systems are relevant to a particular situation<br>▪ Terminology differs between systems and they are difficult to navigate
▪ The Victorian Aids and Equipment Program is administered by Monash Health on behalf of the DHS. The application process is standardised based on tight explicit criteria for transparency and accountability.	▪ This is a 'last resort' process after other sources of funding have been exhausted. Clinicians waste valuable time writing funding applications for multiple programs which could be integrated and allocated centrally.
▪ The Department of Treasury is interested in supporting disinvestment initiatives but requires details of savings. If savings or reinvestments can be quantified the department may provide more funding.	▪ It is hard to measure the savings<br>▪ The savings are rarely realised because they are absorbed and used to treat more patients

Monash Health environment: General	
Enthusiastic and dedicated staff<br>*Staff commitment to quality improvement*<br>*Organisational support*<br>*Support from the Executive Management Team*<br>*Support from Directors of Nursing*<br>*Involvement of people who are outside of, or uninterested in, the politics of the organisation*	*High staff turnover in the organisation, particularly agency nurses and junior staff, increases difficulty in communication and implementation*<br>*High staff turnover in projects diminishes organisational knowledge and expertise and increases training requirements*<br>*Organisational culture is difficult to change*<br>*Organisational politics*<br>*Incident reporting software (Riskman) is flawed, does not cover all requirements and does not enable valid aggregation of data related to consumer information*
▪ Strategic planning provides an opportunity for integrating disinvestment decisions into organisational practices. Monash Health had transparent strategic and business planning processes.	▪ Lack of strategic planning for large equipment purchases
▪ The Board, Executive Management Team (EMT) and Senior Managers have expressed 'patient-centred care' as a priority.	▪ Considerable pressures on the health service to reduce costs.<br>▪ Perceived distinction between 'what the hospital is concerned about (finances, organisational capacity and risk management) and what the clinician is concerned about (patients)'.
**Monash Health environment: Governance**	
**Oversight**<br>▪ Overall accountability sat with the Monash Health Board. The Board and EMT determined the decision-making structures within the organisation.<br>▪ The Quality Unit maintained an organisational chart of committees related to quality and safety.<br>▪ The Board Secretary also had a list of some committees	▪ No central resource for oversight, coordination or provision of information about committee processes<br>▪ No complete list of committees operating at an organisation-wide level<br>▪ No lists of committees operating within programs or sites
**Policies and procedures**<br>*Robust policies and guidelines for purchasing*<br>*Relevant Terms of Reference for committees*	
▪ Nature and scope of decisions was stipulated in the Purchasing Policy, Purchasing Policy Guidelines and Authority Delegation Schedule to prevent gaps, overlap and ambiguity.	▪ Confusion about 'who does what'<br>▪ Duplication of some committee and project activities
▪ In addition to policies and guidelines there were supporting documents such as application forms, business case templates, requisition forms and checklists governing activities related to resource allocation such as purchasing and procurement and development of clinical guidance documents.	*Too much paperwork and existing paperwork is confusing and ambiguous*<br>▪ Some documents were not well organised, not easily accessible, multiple versions were available and some required considerable skills and resources to complete<br>▪ Emphasis on 'business' aspects and less consideration of evidence of safety, effectiveness and cost-effectiveness in many of these documents
**Transparency and accountability**<br>▪ Transparency and accountability in decision-making was highly valued by respondents<br>▪ Improved transparency and accountability at Monash Health was desired by most respondents	*Lack of transparency in all aspects*<br>*Lack of transparency and accountability in decision-making reduces confidence*<br>▪ Inadequate transparency and accountability was one of the strongest messages from respondents
▪ Clear documented lines of accountability and reporting requirements in some areas<br>▪ Individuals and members of committees at the top of their respective decision-making hierarchies reported that they had clear understanding of how the processes should work, who is accountable, who makes the decision, etc and knew the difference between recommendations, decisions and authorisation.<br>▪ Many of these respondents also reported that all decision-makers have the same understanding as they do.	▪ Many individual and group decision-makers lower down the respective hierarchies admitted they were unsure of the processes. Others who said they were sure gave answers that were inconsistent with each other. Some reported ambiguities and inconsistencies in the systems and processes.<br>▪ Confusion between the concepts of 'decision' and 'recommendation' which may lead to uncertainty in accountability. Some committees saw their role as 'recommending' a course of action with the 'decision' being made by a higher-level committee. In contrast, the higher-level committees saw their role as one of guidance and support in response to robust investigation of decision options which they expected to occur at the lower level 'decision-making' committees.<br>▪ Individual decision-makers did not always know who to report a decision to and whether formal authorisation was required.

**Conflict of interest**<br>▪ Conflict of Interest required as a standing item on the agendas of relevant committees. Ten of 13 committees interviewed had a process for conflict of interest for committee members, and two of the four committees with an application process had a similar procedure for applicants.	▪ Only one committee, the Technology/Clinical Practice Committee (TCPC), considered the effect of conflict of interest in the provision of evidence used in decision-making
**Monitoring, evaluation and improvement of systems and processes**<br>▪ Quality improvement of systems and processes was supported by respondents<br>▪ Only one committee (TCPC) had an ongoing process of monitoring, evaluation and improvement of its systems and processes, however some committees had undergone a single evaluation/review and some were developing or planning to develop quality improvement processes.	▪ No formal requirements for quality improvement of decision-making at Monash Health<br>▪ At the program level, it was noted that 'since there was no formal decision-making process there was no process of review'.
▪ Committees that authorise or support decisions made by other committees expected that a rigorous process of decision-making and prioritisation had occurred.	▪ No system to check or regulate this
**Reporting**<br>▪ Quality Unit chart of committees related to quality and safety included lines of reporting<br>▪ Most committees had reporting requirements included in their Terms of Reference	▪ The structure and process of reporting varied with site, department/unit and health professional group making the decisions across and between sites, programs, units, etc difficult<br>▪ No systematic or documented process for reporting of projects
**Monash Health environment: Administration**	
**Relationships, coordination, collaboration and communication**<br>*Knowing who to go to for information*<br>*Knowing who to go to for support*<br>*Networks within the organisation, particularly nursing*<br>*Quality and Risk Managers are good at sharing information across the organisation*<br>*Good communication at site level (nursing)*<br>*Robust and regular communication*	*Lack of knowledge and awareness about*<br>▪ *decision-making systems and processes and where to go to find out about them*<br>▪ *information sources and tools and where to go to find them*<br>*Lack of information regarding how the system works and what processes need to be followed*<br>*Lack of central resource/identified role to provide information about committees*<br>*Lack of organisational processes for knowledge transfer*<br>*Lack of coordination and collaboration between decision-making individuals and groups*<br>*Lack of communication about decisions between programs, departments and other stakeholders*<br>*Lack of communication about impending decisions and projects to enable stakeholder input*
▪ Quality Unit chart of committees included relationships (but only for reporting purposes).<br>▪ Some committees recognised the overlap in their work and the potential to work together. These were in two groups, those considering introduction of new TCPs and those involved in purchasing.<br>▪ People who were members of more than one committee often provided the links between them.<br>▪ There were many examples of cross-unit/department consultation and collaboration for policy and protocol development and implementation.<br>▪ Four projects were linked to others with similar aims.	▪ Lack of awareness of other committees within Monash Health<br>▪ Other than reporting, there were no documented relationships between committees<br>▪ Other than the committees considering new TCPs, there were no formal processes of referral for issues that might affect, or should be addressed by, other committees<br>▪ Decision-making 'in isolation' was noted to be a problem in multiple settings. 'Fragmentation' and a 'silo mentality' were used in relation to decisions made without consideration of the areas they will impact upon or consultation with relevant stakeholders.<br>▪ No systematic processes to link projects across the organisation
**Monash Health environment: Stakeholder engagement**	
Involvement of broad range of stakeholders from multiple sites and a range of health professional disciplines<br>▪ Reported benefits of broad stakeholder involvement in decision-making included improved decision-making, more effective dissemination of decisions and informing and encouraging others about the need to consult with the groups represented.	*Lack of consultation with clinicians in decisions made by managers*<br>*Lack of consideration of impact of change on others when making decisions or planning projects*<br>*Lack of consideration of downstream or lateral impacts eg 'cost saving measures in one area can result in increased costs in another area'*<br>*Limited input from the Quality and the Education Units*
▪ Many respondents supported increased consumer participation and were planning to act upon this	▪ Only one committee (TCPC) included consumer representation in decision-making.<br>▪ Several respondents thought that consumer representation on their committees would be inappropriate or that consumers had insufficient technical understanding to participate.

**Monash Health environment: Resources**	
**Funding and staff time**<br>*Provision of extra staff*<br>*Availability of extra funds enhanced implementation and evaluation, eg introduction of the National Inpatients Medication Chart had external funding specifically for implementation and evaluation*<br>*Some clinical pathways involve no additional costs*	*Staff dissatisfaction with the expectation of their superiors that they will do more work within existing resources*<br>*Insufficient allocation of staff time impairs*<br>▪ *research and preparation for decisions*<br>▪ *implementation and evaluation of decisions*<br>▪ *project delivery*<br>▪ *training*<br>*Lack of/inadequate coordination of current resources*
▪ Some committees had a Secretariat comprised of 1-2 officers from named roles within the organisation. These positions were allocated sufficient time to complete the required tasks.<br>▪ Some projects were provided with adequate resources for implementation and evaluation<br>▪ Some wards had additional staffing for education support and clinical nurse support. These were invaluable resources for practice change, protocol development and implementation.<br>▪ Some projects had external funding from DHS, universities, etc for staff or infrastructure costs.	▪ Some committees used the Personal Assistant of the committee Chair in an administrative role. If a new Chair did not have a personal assistant there would be no resources to support the committee.<br>▪ Some respondents found it difficult to separate the role of the committee from the role of their department. Committee work significantly increased their overall workload, particularly administrative matters, and it was not always clear if these duties were part of, or additional to, their normal duties and what they could cut back in order to accommodate committee obligations.<br>▪ Many projects were to be carried out 'within existing resources'. Respondents noted that they either did unpaid overtime or aspects of the project were not undertaken.
**Expertise and Training**	*Lack of/inadequate skills in*<br>▪ *use of information technology*<br>▪ *finding and appraising evidence from research and data*<br>▪ *project management*<br>▪ *change management*
▪ Staff in Centre for Clinical Effectiveness (CCE) and Clinical Information Management (CIM) were available to decision-makers to provide expertise in research evidence and local data respectively.<br>▪ CCE ran training programs in finding and using evidence, implementation and evaluation<br>▪ Six of 10 projects had training for project staff in change management, leadership or IT skills.	▪ CCE's funding for training was redirected due to budget cuts so it was unable to provide free in-house programs (however many staff attended the fee-paying courses CCE provided)<br>▪ Lack of understanding of information systems and project management in senior decision-makers was reported and training for committee members was suggested<br>▪ Most projects used a staff member from the department involved to deliver the project, most of these did not have project skills or expertise<br>▪ Education and training is not well provided for part-time and night staff.
**Information**<br>*Provision of extra computers*	*Lack of computers and/or access to computers, particularly for nurses*<br>*Difficulties using intranet to find organisational data*
▪ CCE and CIM were available to provide information to decision-makers<br>▪ Monash Health libraries provided access to health databases and electronic journals, as well as advice in searching the health literature.	*Lack of research evidence and local data to inform decisions*<br>▪ Many decision-makers chose not to use these sources of information<br>▪ Priority was given to senior decision-makers and high level decisions; sometimes decisions at lower levels could not be provided with information due to limited resources.
**Decision-makers**	
Broad committee membership<br>*Dedication of committee members*<br>*Depth and range of experience of committee members*<br>*Proactive clinicians who think about improving and moving forward*<br>*High level of skill within medical staff acting as leaders in their specialties*	*Clinical autonomy*<br>*High workload in running a committee with lack of administrative staff*<br>*Difficulty taking off 'clinician hat' and replacing it with 'manager or decision-maker hat'*
▪ Committee membership included a range of relevant stakeholders (except consumers) invited to	*Some clinicians feel that if they are experts in a particular area they should not have to justify*

participate because of their role in the organisation or their knowledge and skills in relevant areas.	*operational decisions*
**Potential adopters**	
Having the appropriate profession engaging others in change process, for example nurses should be implementing projects with nurses, not pharmacists.	*Resistance to change*<br>*Staff cynicism about the importance of changes and relevance to them*<br>*Some clinicians insist on autonomy in their areas of expertise*
**Decision-making process**	
**Identification of need/application**<br>▪ Decisions were instigated by 'top down' direction and 'bottom up' invitation.	▪ General perceptions that<br>· financial drivers were stronger than clinical drivers<br>· impetus for change was ad hoc, there was no systematic or proactive approach<br>· internal bureaucracy and red tape stifled ideas
▪ Some committees had a well-documented application process.	*Complex and time consuming nature of application processes*<br>*People by-pass the system, usually not deliberate but due to lack of awareness of the process*<br>▪ Some applications are driven by pharmaceutical or equipment manufacturers
**Decision criteria**<br>▪ Documenting explicit criteria was generally viewed positively.<br>▪ The committees with application forms had some documentation of criteria.<br>▪ Other decision-making groups and individuals had 'mental checklists' of criteria they considered.	▪ Only one committee (TCPC) and one individual used explicit, documented decision-making criteria.<br>▪ Some committees had no decision-making criteria.<br>▪ Some individual decision-makers strongly rejected documentation of explicit criteria as 'another form of paperwork that will waste clinician's time'.
▪ Most committees considered the Monash Health Strategic Plan, quality, safety, access and equity.<br>▪ All committees considered financial factors.	*Organisational priorities dominated eg*<br>· *'Sound practice is not always affordable practice'*<br>· *'The operational aspects of nursing (Key performance indicators that are reported to DHS) come first and professional aspects comes second'*<br>▪ There was a perception that there was 'too much emphasis on financial return for investment'
**Ascertainment and use of evidence**<br>*Strong knowledge of the literature*<br>*Attendance at conferences*<br>▪ Using research evidence and local data in decision-making was considered to be important.<br>▪ All respondents reported using research evidence and data in decision-making to some extent.<br>▪ Most committees sought a broad membership in order to utilise expertise in the consideration of research evidence and for decision-making with limited evidence.<br>▪ Four out of ten projects sought research evidence from the literature to inform the project.	*Amount of time needed to search the literature or collect data*<br>*Access to evidence is not easy or coordinated*<br>*Lag time between what universities teach and latest research evidence so new staff are not always aware of best practice*<br>*Drug company marketing*<br>▪ Only one committee (TCPC) required explicit inclusion of research and local data and considered the quality and applicability of this evidence. Only one of the projects appraised the evidence used.<br>▪ The other committees had no process to seek evidence from research. When evidence from research and data was used, it was not usually appraised for quality or applicability.<br>▪ Due to difficulty finding uninterrupted blocks of time, slow computers and lack of skills in finding and analysing evidence, decision-makers relied on clinical expertise and advice from colleagues.<br>▪ Appropriate local data was frequently reported to be lacking, unavailable and 'manipulated'.

**Reminders and prompts to consider disinvestment**<br>▪ One application form (TCPC) had an explicit question about what the new technology will replace and what can be disinvested.	▪ "It's all very well to ask the question but it's very hard to get a clinician to say they will stop doing something".
**Deliberative process**<br>*Robust and honest conversations*<br>*Autonomous decision-making*<br>▪ Decision-makers expressed a desire for a documented standard process.<br>▪ Many respondents noted that the main goal of discussion was to reach decisions by consensus.	*Process not seen as priority for some*<br>· *Some committee members do not attend*<br>· *Meetings too short for proper deliberation*<br>*Some decisions made reactively, 'on the run', due to lack of consultation or not following process*<br>*Long lag time between application and decision*<br>▪ Lack of standardised process<br>▪ Many of the current processes were perceived to be unclear, 'ad hoc' and lacking objectivity<br>▪ Lobbying, both covert 'behind the scenes' and overt 'squeaky wheels', was perceived to result in favourable decisions.
▪ Most committees required not only the presence of a quorum to make decisions but also attendance of members with relevant knowledge or expertise to the decision at hand.	▪ Not all committees had a defined quorum. Of those that did, some made decisions in the absence of a quorum and some made decisions even if a meeting was cancelled due to lack of a quorum.<br>▪ Some decisions were made outside committee meetings or by the Chair only.
**Documentation and dissemination**<br>▪ One committee (TCPC) published Decision Summaries which were formally distributed to the Therapeutics Committee, EMT, DHS, the Applicant, Department Head and Program Head and made publicly available on the internet.<br>▪ Most committees recorded minutes; these were considered to be confidential and were not published, but were available to appropriate requestors by contacting the committee secretariat<br>▪ All of the individual decision-makers interviewed reported disseminating decisions to people they considered appropriate and, when deemed necessary, disseminating decisions organisation-wide.<br>▪ Many respondents reported others disseminating decisions to them.	*Large size, nature and diversity of the organisation increases*<br>· *difficulty in dissemination of information*<br>· *frequency and range of communication methods required*<br>*Not everyone uses email*<br>*Using email too often dilutes the effect*<br>▪ The majority of committees did not publish minutes or anything similar.<br>▪ One committee did not keep any records.<br>▪ Although some related committees exchanged minutes there was a lack of formal communication across committees.<br>▪ Documentation and dissemination of decisions made by individuals was informal and ad hoc.<br>▪ Not all projects communicated decisions to other staff members or the wider organisation. Unless people were directly involved, some projects appeared not to make project work or associated decisions public knowledge.<br>▪ Lack of processes for knowledge transfer, especially across sites.
**Implementation**	
**Purchasing**<br>▪ Robust organisational processes that met annual audit requirements<br>▪ Electronic ordering was controlled through an approval hierarchy with delegation thresholds.<br>▪ It was assumed that the decision to purchase was made with due process before reaching the purchasing unit.	▪ Use of evidence in purchasing decisions was not outlined in the Purchasing Policy Guidelines.<br>▪ Those making the decision of 'whether to buy' were responsible for ascertaining evidence of safety, effectiveness and cost-effectiveness in the first stage; however there was no system to check that this has been done before the second stage.
▪ Health Technology Services, the Product Evaluation Committee and working parties set up to evaluate large individual capital purchases considered appropriateness of equipment to Monash Health, availability of spare parts, life expectancy, servicing requirements, related consumables, availability of technical expertise and fit with the DHS Asset Management Framework. They also had expertise in contract negotiation.	*Difficulty managing expectations eg 'once something is approved people want it immediately'*<br>▪ Some were unaware of this process and went directly to the manufacturer. If this was overseas it may be difficult or expensive to get parts, there may not be relevant skills for local maintenance and it excludes benefits that may already exist with a local manufacturer that could supply the same product under better terms and conditions. Re-negotiating contracts, or establishing new ones, creates bad feeling and wastes lots of time.
▪ Purchasing of clinical consumables within budget allocation is done electronically. Electronic authorisation is required for items above individual limits (eg Nurse Unit Manager approval up to $10,000, items above this require authorisation).	▪ There is little assessment of safety, effectiveness or cost-effectiveness of clinical consumable items.

**Policy and guidance**<br>▪ Monash Health was developing a new Policy and Procedure Framework<br>▪ Broad support for increased standardisation of practice through policies and procedures<br>▪ Development process seen as a communication tool between professional groups and across sites.	▪ Lack of structure and standardisation of processes, especially between sites.
**Implementers**<br>*Finding others who have done the same work for support, advice and information*<br>*Establishing Working Parties and Steering Committees for support, endorsement, troubleshooting*<br>*Project leader whose primary role is 'at the coal face'*<br>▪ Decisions made at program level that involve multiple wards, departments or sites are usually implemented by multidisciplinary teams.	▪ Some project staff felt isolated and would have liked support from others who had done the same or similar work<br>▪ It was not always clear who was responsible for project management<br>*Lack of/inadequate project management and communication resulted in multiple people*<br>▪ *making inconsistent changes*<br>▪ *contacting equipment vendors with requests and ideas for change*
**Practice change**<br>*At site level, there is good 'buy-in' for change and people are keen to make things work (nursing)*<br>*Allowing wards to nominate themselves for participation in projects*<br>*'Bottom up' approach to develop individual implementation plan in each ward*<br>*'Bottom up' training to gain staff 'buy in' combined with 'top down' supportive strategy*<br>*Flexible and adaptable staff*<br>*Lots of preparation including training and communication with all stakeholders*<br>*Use of pre-existing (and pre-tested) tools from other organisations*	*Unrealistic project timelines*<br>*Variability in current practice and lack of standardisation increases number of practices to change*<br>*Large size, nature and diversity of the organisation increases complexity of implementation across departments with different needs*<br>*Lack of effective implementation pathways*<br>*Things take a long time to implement, to the point that they 'fall off the agenda'*<br>*Staffing issues, including leave, mean that a lot of projects are on hold*<br>*Project-specific barriers such as logistical challenges with product being implemented*
▪ Some committees provide an approval process only and the applicant is responsible for implementing the decision. In most cases the applicant has control over the process (eg head of department implementing a new procedure) and is motivated to implement the change.	▪ Sometimes practice change is required beyond the applicant and their department. Committees do not require applicants to have or acquire knowledge and skills in implementation.
▪ Training and education activities and 'champions' were reported as the two key strategies used to effect change and encourage sustainability of the intervention.<br>▪ Most projects had a champion and/or Executive sponsor. Project champions were generally the head of the relevant department; others included the Chief Executive Officer, Executive Directors who were Steering Committee Chairs and 'Ward Champions' selected to encourage and promote change.<br>▪ Those with champions unanimously considered champions important to the success of the project.<br>▪ Training or education included passive methods using posters and memos, interactive learning on new equipment and participatory approaches involving staff in design and implementation.<br>▪ Seven projects involved training for the target group, most of which was done by external providers of new equipment.	▪ Lack of knowledge and skills in project management, change management and use of information technology were exacerbated when interventions were complex and required high levels of training<br>▪ Lack of known, standardised processes for implementation at Monash Health
▪ Most considered their project sustainable and believed the change was embedded in the system. This was reportedly achieved by involving a variety of staff and 'bottom-up' approaches to change.	▪ Only two considered sustainability in the design of the project.
▪ Half of the projects tailored the implementation plan to anticipated barriers and enablers sourced from other health services, literature searches and personal experiences of project staff.<br>▪ Half reported that implementation was conducted as planned. Some noted that it mostly went to plan but 'amendments were made continually to improve the process'.	▪ One project had no implementation plan<br>▪ Half of the projects did not consider barriers and enablers
The benefit of the proposed practice change is clear and observable	▪ Lack of baseline data meant that potential adopters were unable to see the benefit or relevance to their situation resulting in less 'buy in' and poor uptake.

Evaluation of outcomes of decisions	
**General**<br>*Use of pre-existing (and pre-tested) tools from other organisations eg audit tools*<br>▪ Evaluation and monitoring were considered important and had broad support<br>▪ Monitoring of projects after implementation was thought to increase sustainability.	*Quality and Risk Managers are not included at the beginning to help with collection of baseline data and evaluation design*<br>▪ Lack of baseline data<br>▪ A lack of data was seen to contribute to the current state of 'little or no process of evaluation'.<br>▪ Limited funds, knowledge and/or skills inhibited both the planning and conduct of evaluation.
**Evaluators**<br>▪ CCE was establishing an in-house Evaluation Service at the time of these interviews.	▪ No specified evaluators with appropriate training or expertise had been utilised by the respondents.
**Requirements for evaluation**<br>▪ Monitoring, evaluation and reporting of outcomes was required by DHS sponsored projects and TCPC. The Therapeutics Committee requested reports for some decisions.<br>▪ Routine clinical audits and monitoring of adverse events undertaken for hospital accreditation purposes provided indirect evaluation of decisions in some situations.<br>▪ Half of the completed projects had been evaluated; all but one project reported achieving its planned objectives.	▪ Monash Health had no requirements for evaluation of outcomes of decisions or projects.<br>▪ Most committees had no planned evaluation of outcomes of decisions or implementation projects.<br>▪ The purpose of reports for TCPC and Therapeutics was questioned by some respondents who noted that it may be inconsistent with the knowledge needed for program staff.<br>▪ Only 2 projects planned evaluation as a project component. Some were evaluated post hoc.
**Reinvestment**	
Reinvestment or reallocation of resources would be an incentive to disinvestment<br>▪ SHARE Steering Committee keen to establish and support methods for reinvestment/reallocation<br>▪ Flexibility and thinking laterally to include novel methods/indicators such as reducing waiting lists, getting patients out of Emergency Department faster, freeing up time in procedural/operating suites, freeing up bed days that are used to treat another patient group faster (eg X procedure saved Y$/bed days which was used by Z patients).	*Lack of planning for resource reallocation*<br>*Lack of transparency and consultation in reallocation of savings creates disillusionment*<br>*Staff dissatisfaction that savings generated are not reallocated*<br>▪ A health economist is required to do this properly, Monash Health had no resources for this<br>▪ 'We don't look far enough for downstream effects; we're too simplistic in assessment of savings'.<br>▪ It was noted that savings made in a project in one area sometimes increased costs in other areas; hence reallocation of the savings to the project department would be unfair.<br>▪ Savings of bed days or time in procedural/operating suites were used immediately to treat another patient group so were never realised<br>▪ Accounting practices did not enable measurement and/or reallocation of savings in some areas, for example changes to one TCP may affect multiple cost centres eg department, ward, ICU, pharmacy.

Section 6 Factors that influenced development of SHARE Program relevant to EDS

Reproduced from Harris et al [6] with permission.

Items related to proactive use of evidence in decision-making are highlighted. The other items are retained to provide context.

Finding	Source	Decision	Program element
Potential benefits of disinvestment identified	Literature	Establish a program exploring disinvestment at Monash Health.	SHARE program
External environment supportive of disinvestment program	Literature & DHS documents		
Internal environment supportive of disinvestment program	Monash Health Staff		
Capacity for leadership in this area demonstrated	Success of new TCP program		
The word 'disinvestment' is associated with negative connotations, high risk of engendering suspicion and distrust and getting stakeholders offside.	Literature<br>Monash Health Staff	Proceed carefully, avoid the term 'disinvestment' and use positive language.	Principles
'Top down' approach seen as negative. Needs to be balanced with 'bottom up' strategies and involvement of stakeholders.	Literature<br>Monash Health Staff	Implement 'top down' and 'bottom up' strategies, make stakeholder engagement a priority, and integrate methods for staff to drive change into the new systems and processes.	Principles<br>Preconditions
A systematic integrated approach would be better than ad hoc decisions, individuals 'championing' causes or projects undertaken in isolation.	SHARE leaders<br>International experts	Focus on organisation-wide approach to decision-making that integrates new and current systems and processes.	Principles
Perceived lack of transparency and accountability and suboptimal use of evidence in current decision-making processes. Power struggles and hidden agendas perceived to influence outcomes.	Monash Health Staff<br>Project team	Ensure the new systems and processes are transparent, accountable and evidence-based.<br>Introduce explicit criteria for disinvestment decisions.	Principles
Lack of transparency and accountability in reallocation of funding released through disinvestment would be significant barrier to effective program.			
Lack of consistent terminology, absence of decision-making criteria and no guidance to inform an organisational approach.	Literature<br>International experts	Develop our own frameworks and methods.	Principles
Disinvestment should not be considered in isolation but alongside other decisions. Investment and disinvestment decisions are often linked, disinvestment occurs when something new is introduced.	Monash Health Staff<br>SHARE leaders<br>Project team	Do not focus on 'disinvestment' or 'investment' alone. Consider 'resource allocation'. Establish processes along decision-making continuum from introduction to removal.	Principles
Health service staff perceive management priorities to be focused on saving money. The concepts around 'disinvestment' accentuate this.	Literature<br>Monash Health Staff	Focus on 'effective application of health resources' to facilitate a positive approach.	Principles
The program needs a strong positive image that reflects the new focus on 'effective application of health resources'. Being compatible with 'iCARE', the familiar acronym for Monash Health values would be beneficial.	Monash Health Staff<br>SHARE leaders<br>Project team	Change the name from 'Disinvestment Project' to 'SHARE' (Sustainability in Health care by Allocating Resources Effectively)	Name
Six potential opportunities to integrate disinvestment decisions into organisational infrastructure, systems and processes were identified.	Literature<br>SHARE leaders	Investigate methods to implement disinvestment decisions in the six settings identified.	Systems and Processes
Undertaking disinvestment projects was a key element of the original proposal. Waiting for investigation of the six settings is too long to delay pilot projects. Some 'quick wins' would be valuable.	SHARE leaders<br>Monash Health Staff	Develop methods to identify and prioritise potential target TCPs in parallel with the investigation of the six settings. Undertake pilot projects to disinvest them.	Disinvestment projects
Current decisions are made 'routinely' or 'reactively'. Introduction of TCPs is based on applications from clinicians or managers and removal of TCPs is based on emerging problems or product alerts and recalls. Research literature and local data could be used 'proactively' to drive health service practice.	Monash Health Staff<br>SHARE leaders<br>Project team	Build on current 'routine/reactive' processes that are done well.<br>Develop new processes to use evidence 'proactively' to drive decisions and/or priority setting.<br>Make these explicit elements of the new program.	Principles
Using evidence 'proactively' requires time and attention from decision-makers. The information provided must be trustworthy, applicable and sufficiently important to	Monash Health Staff<br>SHARE leaders	Develop methods to identify appropriate high-quality information, process and package it for ease of use and deliver it to the	Systems and Processes

Finding	Source	Decision	Program element
warrant adding to their workload.		relevant decision-makers.	
Decisions for resource allocation are delegated to committees and individuals. There are opportunities for improvement in the governance of these processes and to introduce routine consideration of ‘disinvestment’.	Monash Health Staff<br>SHARE leaders<br>Project team	Review processes and governance of decision-making by committees and the authority delegation schedule	Systems and Processes
There is no guidance on consumer participation in disinvestment activities.	Literature	Develop methods to capture and utilise consumer perspectives and integrate them into the new program.	Systems and Processes
With a few exceptions, committees and project teams do not routinely involve consumers in making or implementing decisions and the organisation does not have a framework for engaging consumers.	Monash Health Staff<br>Project team		
The systems and processes for evidence-based decision-making cannot be delivered without appropriate and adequate skills and support	Literature<br>Monash Health Staff	Develop support services that enable capacity-building and provide expertise and practical assistance	Support Services
With a few exceptions, staff do not routinely seek evidence for decisions, are unaware of best practice in implementation and do not evaluate outcomes.	Monash Health Staff<br>Project team	Provide expertise, training and support in accessing and utilising evidence in decisions.<br>Provide expertise, training and support in implementing and evaluating evidence-based change.	Support Services
The main barriers to use of evidence and effective implementation are lack of time, knowledge, skills and resources.	Literature<br>Monash Health Staff		
Health service projects are not usually well supported. It is common for funding to be insufficient, timelines inadequate and staff lacking in knowledge and skills in project management, data collection and analysis.	Monash Health Staff<br>Project team	Influence planning of disinvestment projects to ensure adequate resources and appropriate timelines.<br>Provide expertise, training and support in project methods and administration	Support Services
Disinvestment projects are generally based on health economic principles	Literature	Utilise in-house expertise and take an ‘evidence-driven’, rather than ‘economics-driven’, approach to investigation of disinvestment in the health service context.	Principles
Monash Health does not have expertise in health economics and does not intend to fund this in the foreseeable future	Monash Health Leaders		
Safety, effectiveness, local health service utilisation and benchmarking parameters are possible alternative considerations for disinvestment.	SHARE leaders<br>Monash Health Staff<br>Project team		
Monash Health has high-level expertise in accessing and using research evidence and health service data to inform decisions.			
Monash Health does not have the level of expertise in health program evaluation required for SHARE and has no expertise in health economics.	Project team	Engage consultants in health program evaluation and health economics to assist in development and evaluation	Preconditions
There is no guidance to inform a systematic organisational approach.	Literature	Undertake action research to investigate the process of change in addition to program and economic evaluations.<br>Run a national workshop to learn and share information.<br>Disseminate all findings.	Evaluation and Research
In addition to detailed program and economic evaluation, understanding what happened in the process of investigation, what worked, what didn’t work and why is required.	SHARE leaders<br>Project team		
This large program will need funds. It is consistent with the disinvestment agenda of the Victorian DHS who are sympathetic to a funding application.	DHS documents<br>DHS staff	Seek funding from the state health department.	Preconditions
To be successful this ambitious proposal will need endorsement, support and strategic direction from the highest level and links to those with power and influence in the organisation.	Literature<br>SHARE leaders<br>Project team reflection	Increase membership of the Steering Committee to reflect those best able to provide the appropriate influence, direction and support.	Preconditions
All projects should be aligned to the Monash Health Strategic Goals. Program activities will be facilitated if integrated into the organisation Business Plan.	SHARE leaders<br>Project team reflection	Align SHARE with the Monash Health Strategic Goals and include program activities in the annual Business Plans	Principles

Section 7 Factors that influenced development, processes, outcomes and revision of EDS

a. Development

Influencing factors are presented in the matrix below. Decisions are summarised in the table following.

Development, implementation and evaluation of the pilot Data, Capacity Building and Project Support Services are reported in Paper 7 [7]. Matrix reproduced with permission.

The findings of the initial review [8-22] are consistent with current literature on evidence-based decision-making [23-29], disinvestment and resource allocation [30-40], and information needs of health service decision-makers [16, 41-49]. Recent studies have also demonstrated that dissemination of summaries of synthesised evidence [23, 27, 50-52] and evidence products with targeted messages [23, 27, 53-55] are effective knowledge translation mechanisms. References regarding the evidence of effective strategies have been added to the matrix for completeness.

Influencing factors	EVIDENCE SERVICE			DATA SERVICE			CAPACITY BUILDING SERVICE			PROJECT SUPPORT SERVICE		
	Identify, capture and process synthesised evidence	Translate into user friendly formats	Disseminate to decision-makers	Identify high risks and variations in practice	Translate into user friendly formats	Disseminate to decision-makers	Provide training in accessing and using evidence and data	Provide training in implementation & evaluation	Mentor and support	Provide advice regarding methodologies and methods	Assist with project development & administration	Assist with data capture, data entry and analysis
**BARRIERS**												
Lack of time and opportunity [10, 23, 25, 26, 29, 41, 44, 46-48, 56-59]			✓			✓					✓	✓
Lack of skills [10, 23, 26, 29, 33, 41, 43, 44, 46-48, 59-63]	✓	✓	✓	✓	✓	✓	✓	✓	✓	✓	✓	✓
Lack of confidence [29, 42]	✓	✓	✓	✓	✓	✓	✓	✓	✓	✓	✓	✓
Lack of interest or competing priorities [27, 42, 45, 46, 59]			✓			✓	✓		✓			
Lack of awareness of research and data [10, 23, 27, 29, 60, 62]	✓		✓	✓		✓	✓					
Lack of use of available research and data [27, 34, 60-62]		✓	✓		✓	✓	✓	✓	✓	✓	✓	
Lack of relevant research and data [26, 43, 46-48, 56-58, 60, 64] particularly for disinvestment [30, 33, 39, 48, 59]	✓			✓			✓					
Poor quality of health data [28, 48, 56, 60, 61, 64, 65]				✓	✓	✓						
Unfamiliar or difficult to use formats of research and data [10, 29, 48, 59-61, 64]		✓			✓		✓					
Lack of policies and interventions for data-informed decision-making [56, 60, 66]				✓	✓	✓						
Difficulty accessing or using online resources [10, 26, 27, 29, 41, 43, 44, 46-48, 58, 60, 64]	✓			✓			✓		✓			
Lack of infrastructure and technical support [21, 25, 29, 56, 58, 59, 61, 62, 65]	✓	✓	✓	✓	✓	✓	✓	✓	✓	✓	✓	✓
Inadequate resources [21, 25, 26, 46, 56, 58, 66]	✓		✓	✓		✓					✓	✓
Negative attitudes or resistance to change [23, 25, 29, 59]		✓			✓		✓	✓				
Professional groups with different perspectives of evidence, knowledge base and skill set [30]								✓	✓			
Lack of triggers to initiate disinvestment discussions [31, 34, 36, 38]			✓			✓						

	Identify, capture and process synthesised evidence	Translate into user friendly formats	Disseminate to decision-makers	Identify high risks and variations in practice	Translate into user friendly formats	Disseminate to decision-makers	Provide training in accessing and using evidence and data	Provide training in implementation & evaluation	Mentor and support	Provide advice regarding methodologies and methods	Assist with project development & administration	Assist with data capture, data entry and analysis
Lack of standardised processes for project delivery, responsibilities and accountability [32, 33, 67]								✓	✓	✓	✓	✓
Unrealistic project timelines [32]								✓	✓	✓	✓	✓
**ENABLERS**												
Training in use of evidence and data [10, 29, 41, 61, 65, 66]							✓	✓	✓	✓	✓	✓
Dissemination of research and data [10, 26, 66, 68]			✓			✓						
Clarity, relevance, credibility and reliability of research findings [10, 16, 24, 26, 48]	✓	✓					✓					
Quality and timely data from health information systems [48, 60, 61]				✓	✓		✓					
Organisational willingness to invest in a knowledge translation culture [25, 66, 69]	✓	✓	✓	✓	✓	✓	✓	✓	✓	✓	✓	✓
Infrastructure or policy for accountability in knowledge use [25, 66]			✓			✓						
Links to researchers or knowledge brokers [25, 26, 48, 69, 70]			✓			✓	✓	✓	✓	✓	✓	✓
Initiatives to integrate data into routine decision-making processes [68]				✓	✓	✓						
**ADDITIONAL NEEDS**												
Capacity-building and provision of expertise and practical assistance [10, 28, 35, 37, 40, 60, 62]	✓	✓	✓	✓	✓	✓	✓	✓	✓	✓	✓	✓
New processes to use research and data 'proactively' to drive decisions [28, 37, 60, 65]	✓	✓	✓	✓	✓	✓						
Analysis, synthesis, interpretation and review of data in decision-making [60, 61, 65]				✓	✓	✓	✓	✓	✓	✓		✓
Incentives to change [34, 66, 67]										✓	✓	✓
Support to be tailored to units and professional needs [16, 60, 69]		✓			✓		✓	✓	✓	✓	✓	✓
Provision of a range of expertise in evaluation methods [65, 71]										✓	✓	✓
Support from others who had done the same or similar work to address feelings of isolation							✓	✓	✓	✓	✓	✓
**EVIDENCE-BASED INTERVENTIONS**												
Dissemination of summaries of systematic review evidence [27, 50, 51]		✓	✓									
Tailored targeted messages [27, 53-55]		✓	✓		✓	✓						
Training in critical appraisal [51, 54, 72]							✓		✓			
Interactive workshops [27, 72]							✓	✓	✓			
Multifaceted educational intervention [27, 72]							✓	✓	✓	✓	✓	✓

b. Success and sustainability

Model 1

**SUCCESS:** A proposal is more likely to be successful if it meets the following criteria
**Based on sound evidence or expert consensus**<br>There is evidence of desirable characteristics of evidence products, but no clear evidence of effectiveness for the overall model.
**Presented by credible organisation**<br>Sources of evidence, such as The Cochrane Library, are considered credible. CCE is considered credible as a knowledge broker.
**Able to be tested and adapted**<br>A formal pilot will be undertaken, ongoing feedback will be sought, and systems and processes will be refined based on stakeholder feedback.
**Relative advantage is evident**<br>All stakeholders consulted have responded that they would welcome up-to-date evidence being delivered directly to them.
**Low complexity**<br>Users only have to register to receive evidence, however they will have to appraise it. Reporting template is as simple as possible.
**Compatible with status quo**<br>There is no current system for receiving disseminated evidence. Reporting is integrated into the existing monthly reporting schedule.
**Attractive and accessible format**<br>The email and website formats are attractive and easy to use. The evidence is categorised and readily accessible.
**SUSTAINABILITY:** A proposal is more likely to be sustainable if it has appropriate and adequate provision in each category
**Structure**<br>CCE is an appropriate vehicle to deliver EDS within the organisation. Line management is the appropriate way to report use of evidence, change in practice, etc.
**Skills**<br>CCE team includes systematic reviewers, knowledge brokers and a health librarian. The Monash Health Medical Administration Registrar (trainee) with up-to-date clinical knowledge was seconded to ensure correct classification within clinical categories. The decision-makers may not have the skills to appraise the evidence appropriately.
**Resources**<br>Adequate funding was provided from the SHARE Program and by Monash Health allowing secondment of staff to the EDS.
**Commitment**<br>The organisation has demonstrated commitment through endorsement by the Executive Management Team and the Board and representation on the SHARE Steering Committee (3 executive directors, 10 clinical program directors, 4 committee chairs, 5 senior managers, legal counsel and 2 consumer representatives). All senior decision-makers consulted expressed their support.
**Leadership**<br>The Executive Director of Medical Services and Quality, Chair of the Technology/Clinical Practice Committee and Director of CCE are leaders of the process. All have credibility within the organisation.

Model 2

**SUCCESS:** A proposal is more likely to be successful if it meets the following criteria
**Based on sound evidence or expert consensus**<br>This model addressed the desirable characteristics of evidence products better than Model 1.<br>No evidence of effectiveness for the overall model, no evidence that it has been done before.
**Presented by credible organisation**<br>Sources of evidence, such as The Cochrane Library, are considered credible. CCE is considered credible as a knowledge broker.
**Able to be tested and adapted**<br>A formal pilot will be undertaken, ongoing feedback will be sought, and systems and processes will be refined based on stakeholder feedback.
**Relative advantage is evident**<br>Changes between Models 1 and 2 are based on stakeholder feedback and the benefits of the changes are clear.
**Low complexity**<br>Recipients of Evidence Bulletins only have to check applicability of the evidence and make changes if required. The response form is even simpler and has been reduced from seven responses to two.
**Compatible with status quo**<br>There is no current system for receiving disseminated evidence. Designated decision-makers are responsible for making sure practice in their area of authority is up-to-date.
**Attractive and accessible format**<br>The Evidence Bulletins are attractive, able to be read at a glance, with key information extracted from the publication and summarised.
**SUSTAINABILITY:** A proposal is more likely to be sustainable if it has appropriate and adequate provision in each category
**Structure**<br>Designated decision-makers for the topic under consideration are the appropriate recipients of Evidence Bulletins.<br>Program Directors are the appropriate individuals to disseminate the evidence and request a response from the decision-makers who report to them.<br>The Technology/Clinical Practice Committee (TCPC) is the appropriately authorised group to govern the EDS process.<br>CCE is an appropriate vehicle to develop the evidence products.
**Skills**<br>CCE team have the relevant skills to produce the Evidence Bulletins.<br>The TCPC and Program Directors have the relevant knowledge to assess applicability of the evidence and need for change within the organisation.
**Resources**<br>Funding has been provided by Monash Health for the piloting phase, but ongoing funding to enable continuous delivery of the EDS will be needed.<br>The current level of funding does not enable dissemination of all available evidence; limitation of selected publications to areas of priority within the organisation will be required.
**Commitment**<br>The Chief Executive has made EDS an organisational priority and requires notification of all responses related to evidence of harm.
**Leadership**<br>The Executive Director of Medical Services and Quality, Chair of the Technology/Clinical Practice Committee and Director of CCE are leaders of the process. All have credibility within the organisation.

c. Model 1 Pilot

Domain	Influencing factors	Decisions/Action
**Evidence products**	▪ The quality, currency, content, format and methods of delivery of the EDS were all viewed positively	▪ These features were retained
**Target audience**	▪ Users were not certain about the purpose of EDS and why specific publications were not being disseminated. They were also not using the website search function.	▪ The EDS explanatory pages were revised and a 'Frequently asked questions' page was introduced.
**Knowledge brokering**	▪ The EDS process was complex and only one staff member was familiar with all the requirements, creating problems when they were on leave.	▪ An administrator's manual was developed and additional staff were trained to improve sustainability of the service.
	▪ The pilot website had no branding, which did not comply with internal standards for Monash Health publications.	▪ The Public Affairs and Communications Department assisted the EDS team to include Monash Health branding
**Processes and infrastructure**	▪ Executives, Senior Managers and Program Directors required information about policy and management decisions which was not addressed in the predominantly clinical evidence provided from the sources previously identified.	▪ The category of 'Evidence based policy and management advice' was added and criteria to identify high quality sources of this information were developed (Section 9).
	▪ The need for users to identify publications that recommended ceasing or restricting a TCP for evidence of harm or lack of effect was noted.	▪ The category of 'Disinvestment' was added
	▪ The initial taxonomy used first level ICD10 headings. This did not provide enough detail and half way through the pilot period this was changed to the second level. The change to second level headings within the limitations of the free software made the process of entering data very time intensive and created messy search results for users.	▪ ICD10 classifications were replaced with MeSH.
	▪ The category of 'Professional Group' was thought to be too broad to be of real use, for example 'Medicine' was attached to almost every piece of evidence, and had considerable overlap with the 'Specialty' category.	▪ 'Professional Group' was removed and 'Specialty' was modified slightly to accommodate this change
	▪ The Medical Administration trainee was unable to undertake the classification due to other commitments which were given greater priority. This was a limitation of the Medical Administration portfolio where crises requiring immediate attention occurred frequently.	▪ The EDS paid a medical graduate for one hour per week to ensure categorisation was correct and completed on time.
	▪ Users reported a preference for shorter emails with fewer entries.	▪ Distribution was changed from fortnightly to weekly with fewer entries.
	▪ Citations in bulletins from EUROSCAN did not point to full text.	▪ EUROSCAN was removed from the list of sources of evidence.
**Evaluation plan**	▪ The free email software had significant limitations related to analysis of available statistics. (Separate email software was needed at the start of the pilot as the website software did not have an email subscription function but introduced it later so the separate email software was no longer needed)	▪ The email service with the original provider was discontinued and re-established with the website provider

d. Model 1 Full implementation

Domain	Influencing factors	Decisions/Action
**Evidence products**	▪ Although they were recent publications, they may not contain any new evidence eg update of SRs or HTAs with no changes<br>▪ Although the sources of evidence were appraised for their requirements of rigorous methods, this does not guarantee that the publication is valid or has low risk of bias<br>▪ There was a large volume of information, including a large number of publications that did not require action<br>▪ The email Alerts did not contain many of the features known to increase use and application of disseminated evidence ie no targeted message, no specific request for action	To repackage the evidence to highlight key messages, demonstrate local relevance and implications, and provide actionable recommendations.
**Target audience**	▪ Lack of time to appraise for quality and applicability, check for consistency with current documented practice or complete the proposed reporting template<br>▪ Findings were often irrelevant to recipient's areas of practice, already known to them<br>▪ Wasted their time and increased the potential for them to miss findings that mattered	To reduce the burden on busy decision-makers by filtering publications before dissemination to assess quality, applicability, lack of or inconsistency with policies and procedures, local importance and potential for change.
	▪ Evidence Alerts not always reaching the right decision-makers – self selected	To deliver the repackaged evidence to a specified authorised decision-maker responsible for practice in the areas addressed in the publication.
**Knowledge brokering**	▪ The EDS team had difficulty processing the large number of eligible publications within the available resources and proposed that the selection criteria be restricted to reduce the volume	To limit selection criteria for publications to areas of high priority within Monash Health.
**Processes and infrastructure**	▪ Lack of governance, particularly a lack of transparency and accountability. EDS broadcasts were developed and disseminated rigorously and systematically, but were not accessed or used rigorously or systematically. Those responsible for decisions within the organisation were required to self-select and take action, but there was no process to ensure that the appropriate person with authority in the area affected by the evidence had considered the information or made a decision. Recipients could choose whether to access, use, or report use of evidence; or not.	To introduce a governance framework for transparency and accountability and to ensure that the appropriate decision-makers are engaged, they address the evidence and take action as required, and the process is documented and reported.
**Local considerations**	▪ Although most publications were relevant to Monash Health because it covered such a wide range of clinical areas they may not be applicable if Monash Health does not service a particular population, have expertise in a particular procedure, etc<br>▪ Although there may be high quality strong evidence, practice change may not be important or worth the effort of change processes in preference to other needs, or action may not be required if Monash Health policies and procedures are already consistent with the evidence	To introduce steps that address these local considerations

e. Model 2 Pilot

Domain	Influencing factors	Decisions/Action
**Evidence products**	▪ The critical appraisal findings could be expressed more succinctly to increase ease of use by decision-makers	▪ The quality appraisal summary table was removed and replaced with statements regarding the findings and their implications
**Target audience**	▪ The authorised decision-makers for the areas addressed by the evidence were readily identified	▪ This was an enabler
**Knowledge brokering**	▪ The Evidence Bulletin could be improved to make completion easier for the EDS administrator	▪ Drop-down boxes were introduced into the template
	▪ It was often difficult to interpret authors' conclusions even after reading the whole article	▪ Publications were only disseminated when EDS team were confident that the findings were valid.
**Processes and infrastructure**	▪ Evidence of benefit could not always be classified as clinical or cost effectiveness; for example effective methods to develop or implement guidelines.	▪ A new category of methodological effectiveness was added.
	▪ There was not enough time to discuss the potential items for dissemination at the TCPC meeting	▪ A standing item for EDS was introduced to the TCPC agenda
	▪ EDS was promoted as an organisation-wide priority<br>▪ Responses were mandatory, would be audited and reported to Chief Executive every month<br>▪ TCPC had the authority to require action<br>▪ All senior managers were supportive	▪ These were enablers

f. Model 2 Full implementation

Domain	Potential influencing factors	Potential Decisions/Action
**Evidence products**	▪ No negative comments were received regarding the Evidence Bulletins	▪ The format could be replicated in subsequent models
**Target audience**	▪ The volume of information to each decision-maker was significantly reduced<br>▪ Most bulletins were provided for information only, on average responses were required only once every few months.<br>▪ All the bulletins decision-makers received were relevant to their clinical area<br>▪ Their workloads were reduced to confirming whether change was needed, taking action if required, and reporting the outcomes	▪ These were enablers
	▪ Many decision-makers in the target audience were researchers familiar with the literature and often contributors to systematic reviews or evidence-based guidelines. They were annoyed when receiving material they were familiar with.	▪ Difficult to know how to address this when EDS staff do not know which areas of research staff members are active in, and should not assume even if they are active that they are aware of all the evidence in that area
**Knowledge brokering**	▪ Several respondents appeared to be unclear about the purpose of the EDS, in particular it was perceived that CCE had undertaken the reviews, rather than capturing synthesised evidence as it was published by others	▪ A flowchart or text summary of the EDS process within each bulletin may address this
	▪ Evidence regarding drugs that were not available locally was disseminated	▪ Confirmation that drugs or other technologies are available would require an extra step in the process
	▪ Many publications had more than one conclusion, eg harm plus effect or effect plus lack of evidence.<br>▪ Some complex issues were relevant to multiple decision-makers	▪ New methods are needed to address these issues.
**Processes and infrastructure**	▪ The governance elements worked smoothly and enabled transparency and accountability of the processes<br>▪ The methodological issues were addressed successfully; only valid evidence was disseminated in bulletins that highlighted key messages, demonstrated potential inconsistency with local practice, and clearly stated required actions	▪ These were enablers

Section 8 Options considered in development of EDS

Resources	Capturing	Processing	Storage	Dissemination	Utilisation
**New technologies**<br>▪ Horizon Scanning databases<br>▪ HTA databases<br>▪ Clinica Journal<br>▪ Scrip Journal<br>**Evidence-lacking technologies**<br>▪ HTA databases<br>▪ Cochrane<br>▪ TRIP<br>▪ PubMed Clinical Queries<br>**Benchmarking**<br>▪ Annual Reports<br>▪ Internal datasets eg CIM<br>▪ External datasets eg AIHW, CHA, WHA, WHO<br>▪ Guidelines<br>**Alerts and recalls**<br>▪ TGA email alerts<br>▪ FDA email alerts<br>▪ MHRA email alerts<br>▪ Scrip Journal<br>**Health Policy Issues**<br>▪ Government communications<br>▪ Organisations (AIHW etc)<br>▪ Conference proceedings<br>▪ Journal articles<br>**Guidelines**<br>▪ SIGN<br>▪ NICE<br>▪ TRIP<br>▪ NGC<br>▪ Medscape WIR<br>▪ Individual hospital's guidelines	**E newsletters:** daily<br><br>**RSS feeds:** fortnightly<br><br>**Websites**<br>▪ Cochrane Library: quarterly for new and updated reviews<br>▪ Other web accessible databases (e.g. TRIP, NGC, Q&A services etc): monthly<br>▪ Annual reports (online or print) annually or twice yearly<br><br>**Human interaction**<br>▪ Note-taking<br>▪ Memory<br>▪ Communication<br>▪ Conference and workshop attendance<br><br>**Clinical Information Management**<br>▪ As needed. CIM will extract information from their database and send to requestor in a report/spreadsheet	**Format**<br>▪ How will information be presented to various groups?<br>▪ Will we develop standardised forms?<br><br>**Classification**<br>Multiple systems available<br>▪ ICD 10<br>▪ MeSH<br>▪ SNOMED<br>▪ Data dictionaries<br><br>**Holding**<br>Will information we capture be extracted into a temporary holding place (e.g. Endnote) until ready for processing?	**Options available to us now**<br>▪ Endnote (problems with record limits and slowness due to stored documents)<br>▪ Individual drive and personal hard disks<br>▪ Access database (need IT to create and training to use)<br>▪ Shared drive (public)<br>▪ SH intranet<br>**Options we could invest in**<br>▪ Blogging software on SH intranet (enable anonymous discussion)<br>▪ RSS Aggregators (newsreaders)<br>▪ BookCat (based on Access, modifiable, able to create reports)<br>▪ A document repository system on the intranet (could store finished reports here, as well as use it as an email archive)<br>**Time in storage**<br>▪ Permanent (change in practice, evidence reports, etc)<br>▪ Temporary (alerts/recalls)<br>▪ Immediate deletion (weekly email roundups eg eCAB, Medscape etc)<br>**Legalities**<br>▪ Copyright restrictions (documents obtained under interlibrary loan need to be destroyed after intended use etc.)<br>▪ Need to find out SH's legal record-keeping responsibilities	**CCE current practices**<br>▪ Emails to interested individuals<br>▪ Classes and workshops<br>▪ Conference presentations<br>▪ Journal articles<br>▪ Commissioned reports to internal and external client groups<br>▪ Reports on the old CCE website<br><br>**New practices**<br>▪ SH Intranet<br>▪ Newsletters (CE, SH News, Purple Peril, Nursing & Midwifery (including guidelines)<br>▪ Education (medical and nursing learning portals)<br>▪ Health Information Services<br>▪ Protocols and Guideline site<br>▪ CCE webpage<br>▪ Targeted emails (Heads of Depts, Committee members, senior staff) who can then impart to junior staff<br>▪ Internal newsletters<br>▪ Hospital-wide and group emails<br>▪ Print and distribute entire documents at committee meetings, pass onto interested individuals etc<br>▪ Google group discussion list (available via email and RSS, enables anonymous discussion)<br>▪ Emails to individuals asking what emerging trends are happening in their field	**CCE current practices**<br>▪ Evidence requests<br>▪ Journal clubs<br>▪ Participation on SH committees<br><br>**New practices**<br>▪ Training programs<br>▪ Support systems<br>▪ Reporting systems<br>▪ Project support processes<br><br>**External activities**<br>▪ Journal articles<br>▪ Conference presentations<br>▪ Lectures / Seminars<br>▪ Promotional activities

Abbreviations: AIHW Australian Institute of Health and Welfare; CCE Centre for Clinical Effectiveness; CE Chief Executive; CHA Children's Hospitals Australia; CIM Clinical Information Management; FDA Food and Drug Authority; HTA Health Technology Assessment; ICD 10 International Statistical Classification of Diseases and Related Health Problems Tenth Revision; IT Information Technology; MeSH Medical Subject Heading; MHRA Medicines and Healthcare products Regulatory Agency; NGC National Guideline Clearinghouse; NICE National Institute for Health and Care Excellence; RSS Really Simple Syndication, SH Southern Health; SIGN Scottish Intercollegiate Guideline Network; SNOMED Systematized Nomenclature of Medicine; TGA Therapeutic Goods Authority; TRIP Turning Research into Practice, WHA Women's Hospitals Australia; WHO World Health Organisation,

Storage decision: WordPress (wordpress.com) blogging software was chosen because it was easy to set up and maintain; had a professional appearance; included in-built categories, the choice to turn off comments, a variety of widgets such as search boxes and category drop-down lists, and the ability to store documents within the blog.

Section 9 Definitions of evidence products, inclusion criteria and appraisal of publication sources

Inclusion and appraisal criteria were applied to methods published on the websites of potential sources of high quality synthesised evidence.

Generic criteria

- Publications are in English or have English summaries of foreign language evidence
- Evidence must be freely accessible and require no cost to subscribe or register
- The evidence must be electronically accessible for a period of time (ie stable links)
- Declarations of conflicts of interest and attributions of authorship must be clear and immediately identifiable
- Funding sources must be explicit. If funded by commercial entities, editorial independence must be demonstrated

Systematic Reviews and Health Technology Assessments (HTAs)

A **systematic review** synthesises the results from all available studies in a particular area and provides a thorough analysis of the results, strengths and weaknesses of the collected studies. A systematic review addresses a focused, clearly formulated question. It uses systematic and explicit methods to identify, select and critically appraise relevant research and to collect and analyse data from the studies that are included in the review. It may or may not include a meta-analysis which summarises the statistical results of included studies.[1]

A **health technology assessment** is an evaluation of the clinical effectiveness, cost effectiveness, and broader impact of drugs, medical technologies, and health systems, both on patient health and the health care system. During the assessment, data from research studies and other scientific sources are systematically gathered, analysed and interpreted. The findings from this process are then summarised in reports that translate scientific data into information that is relevant to decision-making.[2]

Quality criteria

- Focused research question(s)
- Comprehensive search strategy
- Specified inclusion and exclusion criteria
- Quality assessment of included information/studies
- Summary of results of individual studies

Evidence-Based Guidelines

Evidence-based guidelines are systematically developed statements that aim to assist practitioner and patient decisions about appropriate health care for specific clinical circumstances. Developed after the systematic retrieval and appraisal of information from the literature, evidence-based guidelines usually include strategies for describing the strength of the evidence, and clearly separate expert opinion from the best available evidence.[3] Evidence-based guidelines have been sourced from sites or organisations that have appropriate methods of development.

Quality criteria

Sources were assessed against a subset of criteria from the AGREE II instrument.[4]

- Systematic methods were used to search for evidence – criterion 7
- The criteria for selecting the evidence are clearly described – criterion 8
- The methods used for formulating the recommendations are clearly described – criterion 10
- There is an explicit link between the recommendations and the supporting evidence – criterion 12

---

1. Centre for Clinical Effectiveness. 2009. Evidence-Based Answers to Clinical Questions for Busy Clinicians. The Centre for Clinical Effectiveness, Southern Health, Melbourne, Australia. http://www.southernhealth.org.au/icms_docs/2145_EBP_workbook.pdf
2. The Canadian Agency for Drugs and Technologies in Health (CADTH). http://www.cadth.ca/index.php/en/hta/faq
3. McKinlay E, McLeod D, Dowell T & Howden-Chapman P. 2001. Clinical Practice Guidelines: A selective literature Review, Report prepared by the Wellington School of Medicine for the New Zealand Guidelines Group Inc. http://www.nzgg.org.nz/download/files/wsm_literature_review.pdf
4. AGREE. 2009. Appraisal of Guidelines for Research and Evaluation II. The AGREE Next Steps Consortium. http://www.agreetrust.org/?o=1397

Horizon scanning documents

Horizon scanning provides short, rapidly completed, 'state of play' documents. These provide current information on technologies to alert planners and policy makers of the advent and potential impact in terms of safety and cost, before they are introduced into the health system. In addition to new and emerging technologies, horizon scanning can also provide timely information about changes in the delivery and use of existing technologies.[5]

Quality criteria

Sources were assessed against the eight principles of the HONcode (Health on the Net Foundation).[6]

- Authoritative
- Complementarity
- Privacy
- Attribution
- Justifiability
- Transparency
- Financial Disclosure
- Advertising policy

Alerts and recalls

An alert is advice regarding a specific situation in which a therapeutic good which, whilst performing to meet all specifications and therapeutic indications, might present an unreasonable risk of substantial harm if certain specified precautions in regard to its use are not observed.[7]

A recall advises the permanent removal of therapeutic goods from supply or use for reasons relating to deficiencies in the quality, safety or efficacy of the goods.[7]

Alerts and recalls were not appraised but were limited to Australian government publications.

Evidence-based policy and management advice

Evidence-based policy and management advice is represented as synthesised research evidence related to governance, financial and delivery arrangements in health systems[8] as well as policies, programs and interventions at public health decision-making levels.[9]

Quality criteria

- Aim of the source is to enable Evidence-Based Decision-Making
- Original full text article freely available online
- Classified as 'strong evidence' by source of publication

---

5. Australian and New Zealand Horizon Scanning Network (ANZHSN). http://www.horizonscanning.gov.au/internet/horizon/publishing.nsf/Content/process-2#what
6. HONcode (Health On the Net Foundation) http://www.hon.ch/cgi-bin/HONcode/Inscription/site_evaluation.pl?language=en&userCategory=individuals
7. Therapeutic Goods Administration. Uniform Recall Procedure for Therapeutic Goods. 2004 edition ©. Commonwealth of Australia. http://www.tga.gov.au/docs/pdf/urptg.pdf
8. Health Systems Evidence. 2011. Health Systems Evidence - Evidence to support decision-making – An online repository of synthesized research evidence for health system policymakers, managers and stakeholders http://www.healthsystemsevidence.org/images/stories/documents/mhf-tool_3_healthsystemsevidence_2010-04-21.pdf
9. Health Evidence Canada. 2011. http://www.health-evidence.ca/html/AboutUs

Section 10 Sources of synthesised evidence

Systematic reviews, HTAs and Evidence-based Guidelines

- Cochrane Library
- Scottish Intercollegiate Guidelines Network
- New Zealand Guidelines Group
- National Institute for Health and Clinical Excellence (NICE)
- National Institute for Health and Clinical Excellence (NICE) – "Do not Do Database"
- Australian National Health and Medical Research Council
- Washington State Health Care Authority HTA Program
- Australian Medical Services Advisory Committee
- Institute of Work and Health
- Health Information and Quality Authority
- Effective Public Health Practice Project
- Centre for Clinical Effectiveness
- Centre for Reviews and Dissemination
- California Technology Assessment Forum
- California Health Benefits Review Program

Horizon Scanning

- Australia New Zealand Horizon Scanning Network
- Canadian Agency for Drugs and Technologies in Health Horizon Scanning Service
- International Network on New and Emerging Health Technologies (EuroScan)

Alerts and recalls

- Australian Therapeutics Goods Administration
- National Prescribing Service
- Any alerts or recalls distributed through Monash Health internal systems

Evidence-based policy and management advice

- Health Systems Evidence (McMaster Health Forum) (Canada)
- Health Evidence Canada

Coding

The titles were coded so the reader could identify the type of publication

- Systematic reviews and health technology assessments (HTAs) were identified by the prefix SR.
- Evidence-based guidelines were identified by the prefix GL.
- Horizon scanning can be identified by the prefix HS.
- Alerts and recalls can be identified by the prefix AR.
- Evidence-based policy advice can be identified by the prefix PL.

Section 11 Taxonomy

Categories

Bibliographic Source, Healthcare setting, Type of technology/practice, Professional group, Specialty, Disease group, Age, Gender, Outcomes, Author's Recommendations and Links to original documents.

Definitions

Healthcare settings

'Settings' refers to the places where healthcare is undertaken. Sources of individual definitions are cited.

- **Inpatient** (Monash Health Acute Care): where the patient requires admission to the hospital; "persons admitted to health facilities which provide board and room, for the purpose of observation, care, diagnosis or treatment" (Mondofacto Medical Dictionary 2008).
- **Outpatient** (Monash Health Continuing Care): where treatment occurs without admission, often on a continuing basis; "a patient who is receiving ambulatory care at a hospital or other facility without being admitted to the facility. Usually, it does not mean people receiving services from a physician's office of other program that also does not provide inpatient care" (Academy Health 2004).
- **Emergency Department**: "a hospital room or area staffed and equipped for the reception and treatment of persons with conditions (as illness or trauma) requiring immediate medical care" (Meriam-Webster's Medical Dictionary, 2010)
- **Organisation-wide**: Information catalogued with this subject heading (e.g. hand-washing, staff wellbeing, patient information) needs to be addressed by multiple departments.
- **General Practice**: A service which provides primary care, generally privately operated; "a term for physicians who care for all types of medical problems. Has since been replaced by more extensively trained family practitioners" (Mondofacto Medical Dictionary 2008).
- **Community Health Service**: provides mixed preventive and primary care; "Community health… has [a] focus on health promotion and disease prevention and management is designed to improve the health and wellbeing of local residents, as well as take pressure off the acute care health system." Services are provided locally, to everyone, irrespective of income. http://www.betterhealth.vic.gov.au/bhcv2/bhcarticles.nsf/pages/Community_health_centres?open

Types of technology /practice

This list was determined by the Technology/Clinical Practice Committee which has the role of approving the commissioning and decommissioning of health technologies and clinical practices at Monash Health. Definitions are based on National Library of Medicine Medical Subject Headings (MeSH).

- **Pharmaceuticals:** Drugs intended for human or veterinary use, presented in their finished dosage form. Included here are materials used in the preparation and/or formulation of the finished dosage form.
- **Implantable Devices:** Devices which are inserted into an organism, typically beneath the epithelium tissue layer, for prosthetic, diagnostic, therapeutic, or experimental purposes.
- **Prostheses:** Artificial substitutes for body parts, and materials inserted into tissue for functional, cosmetic, or therapeutic purposes.
- **Surgical Procedure:** Procedure that either uses open invasive surgery, closed or local surgery, corrects deformities and defects, repairs injuries, diagnoses and cures certain diseases, is elective surgery, or is a procedure to reconstruct, restore, or improve defective, damaged, or missing structures.
- **Surgical Devices:** Nonexpendable and expendable apparatus used during surgical procedures, including surgical instruments (devices that are usually hand-held and used in the immediate operative field).
- **Diagnostic Procedures:** Methods, procedures, and tests performed to diagnose disease, disordered function, or disability.
- **Diagnostic Devices:** Instruments or tests used in medical diagnosis / Nonexpendable items used in examination.
- **Medical Procedure:** A course of action intended to achieve a result in the care of admitted patients, used by medical personnel.
- **Medical Device:** Expendable and nonexpendable equipment, supplies, apparatus, and instruments that are used in diagnostic, therapeutic, scientific, and experimental procedures.
- **Clinical Procedure:** All other procedures or clinical activities

Professional Specialties

This is a modified version of MeSH Health Occupations [H02], originally developed by the National Library of Medicine (http://www.nlm.nih.gov/mesh/2010/mesh_browser/MBrowser.html).

A<br>Acupuncture<br>Adolescent Medicine<br>Adolescent Psychiatry<br>Aerospace Medicine<br>Allergy and Immunology<br>Anaesthesiology<br>Andrology<br>Animal Nutrition Science<br>Audiology	B<br>Bariatric Medicine<br>Behavioural Medicine<br>Biological Psychiatry<br>Biomedical Engineering	C<br>Cardiology<br>Child Nutrition Sciences<br>Child Psychiatry<br>Chiropractic<br>Clinical Medicine<br>Colorectal Surgery<br>Community Dentistry<br>Community Health Nursing<br>Community Medicine<br>Community Psychiatry<br>Critical Care Medicine	D<br>Dietetics<br>Dental General Practice<br>Dental Research<br>Dental Technology<br>Dermatology<br>Disaster Medicine	E<br>Emergency Medicine<br>Emergency Nursing<br>Endocrinology<br>Endodontics<br>Epidemiology<br>Environmental Health<br>Environmental Medicine<br>Epidemiology<br>Ethnopharmacology
F<br>Family Nursing<br>Family Practice<br>Forensic Dentistry<br>Forensic Medicine<br>Forensic Nursing<br>Forensic Psychiatry	G<br>Gastroenterology<br>Geriatric Dentistry<br>Geriatric Nursing<br>Geriatric Psychiatry<br>Geriatrics<br>Gynaecology	H<br>Haematology<br>Health Physics<br>Health Promotion<br>Health Services Administration<br>Health Services Research<br>Herbal Medicine<br>Holistic Nursing<br>Hospitalists<br>Hospital Administration	I<br>Immunology<br>Infection Control<br>Infectious Disease Medicine<br>Integrative Medicine	J, K, L
M<br>Medical Genetics<br>Medical Illustration<br>Medical Oncology<br>Medical Sociology<br>Medical Technology<br>Midwifery<br>Military Dentistry<br>Military Medicine<br>Military Nursing<br>Military Psychiatry<br>Mortuary Practice	N<br>Naval Medicine<br>Nephrology<br>Neonatal Nursing<br>Neonatology<br>Neurology<br>Neuropharmacology<br>Neurosurgery<br>Nuclear Medicine<br>Nursing<br>Nursing Research<br>Nutritional Sciences	O<br>Obstetrical Nursing<br>Obstetrics<br>Occupational Dentistry<br>Occupational Health Nursing<br>Occupational Medicine<br>Occupational Therapy<br>Oncologic Nursing<br>Oral Medicine<br>Oral Pathology<br>Oral Surgery<br>Orthodontics<br>Orthopaedic Nursing<br>Operative Dentistry<br>Ophthalmology<br>Optometry<br>Oral Medicine<br>Oral pathology<br>Oral surgery<br>Organization and Administration<br>Orthodontics<br>Orthoptics<br>Orthopaedics<br>Osteopathic Medicine<br>Otolaryngology	P<br>Paediatrics<br>Paediatric Dentistry<br>Paediatric Nursing<br>Palliative Care<br>Paramedicine<br>Perinatology<br>Perioperative Nursing<br>Pathology<br>Periodontics<br>Pharmaceoepidemiology<br>Pharmaceutical Technology<br>Pharmacogenetics<br>Pharmacology<br>Pharmacy<br>Physical Therapy<br>Plastic Surgery<br>Podiatry<br>Preventive Medicine<br>Prosthodontics<br>Psychiatric Nursing<br>Psychiatry<br>Psychology<br>Psychopharmacology<br>Public Health<br>Public Health Dentistry<br>Public Health Nursing<br>Pulmonary Medicine	Q<br>Quality of Health Care
R<br>Radiation Oncology<br>Radiologic Technology<br>Radiology<br>Regenerative Medicine<br>Rehabilitation<br>Rehabilitation Nursing<br>Reproductive Medicine<br>Rheumatology	S<br>School Dentistry<br>School Nursing<br>Serology<br>Sleep Medicine<br>Speech Language Pathology<br>Sports Medicine<br>Social Medicine<br>Surgery	T<br>Telemedicine<br>Thoracic Surgery<br>Toxicology<br>Transcultural Nursing<br>Traumatology<br>Tropical Medicine	U<br>Urology	V<br>Venereology<br>Vaccination<br>Vascular Surgery

Acupuncture

Allied Health

Biomedical Engineering

Chiropractic

Dentistry

Environmental Health

Health Services Administration

Hospital Administration

Medical Illustration

Medical Sociology

Medicine

Mortuary Practice

Nursing

Nutritional Sciences

Optometry

Orthoptics

Pharmaceutical Technology

Pharmacology

Pharmacy

Podiatry

Serology

Special Interest Groups

These categories are RSS feeds that have been set up for special interest groups.

***Clinical Risk**:* Medical Procedure, Clinical Procedure, Organisation-wide, Infection Control, Nursing, Falls.

***Medication Safety**:* Pharmaceuticals, Pharmaceutical Technology, Pharmacy, Potassium, Insulin, Narcotics (opioid analgesics), Chemotherapy, Heparins, Administration errors, Prescribing errors, Dispensing errors, Electronic prescribing.

***Emergency**:* Emergency Department, Emergency Medicine, Emergency Nursing, Toxicology

***Disinvestment**:* Not recommended, evidence of harm

Section 12 Model 1 Examples of the EDS Website

PILOT VERSION

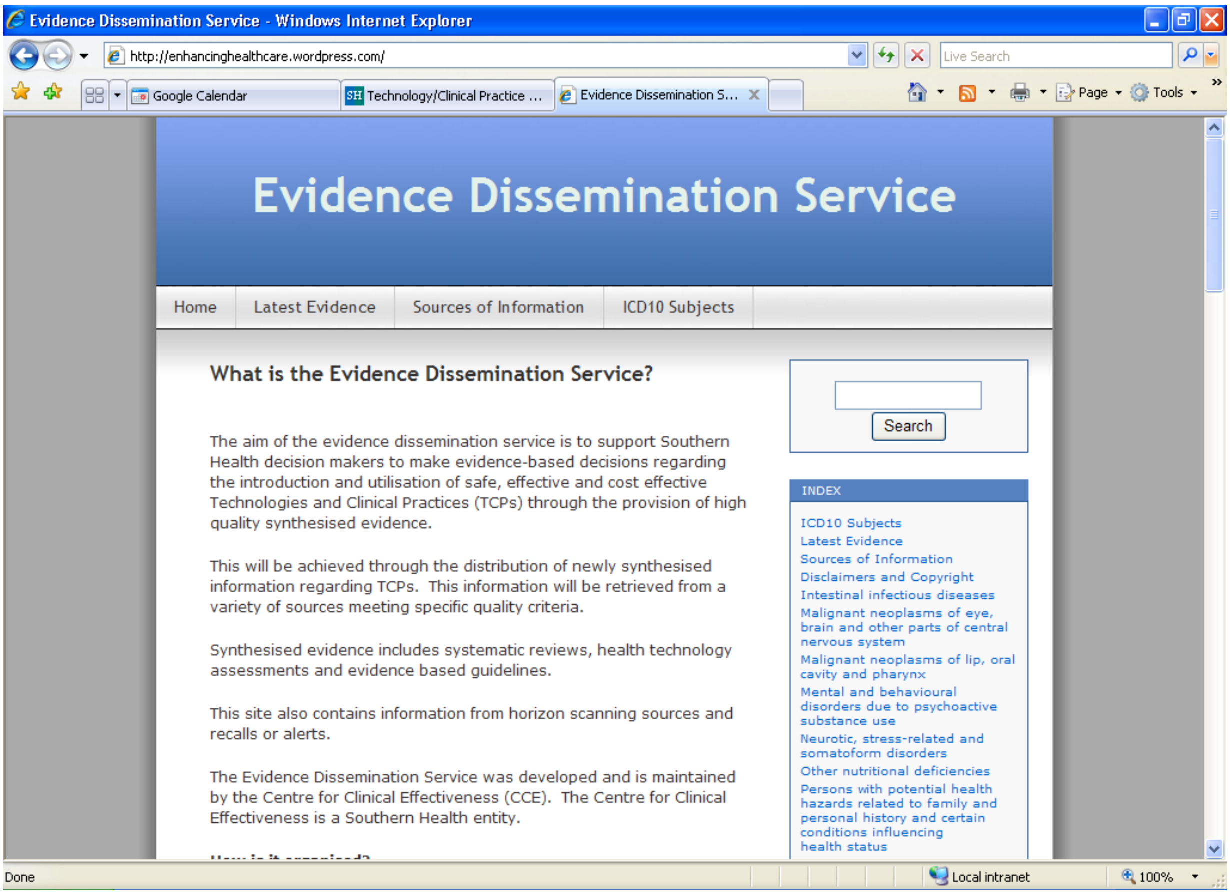

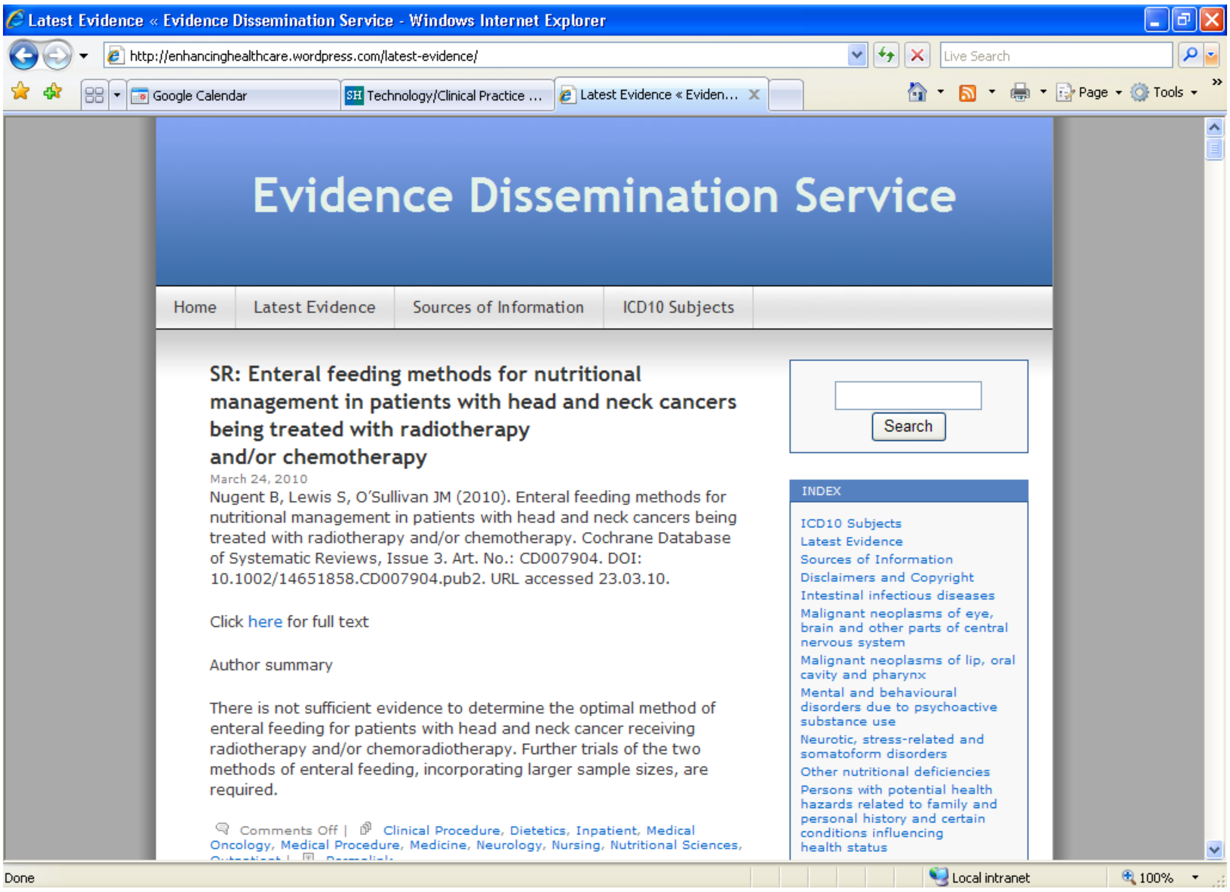

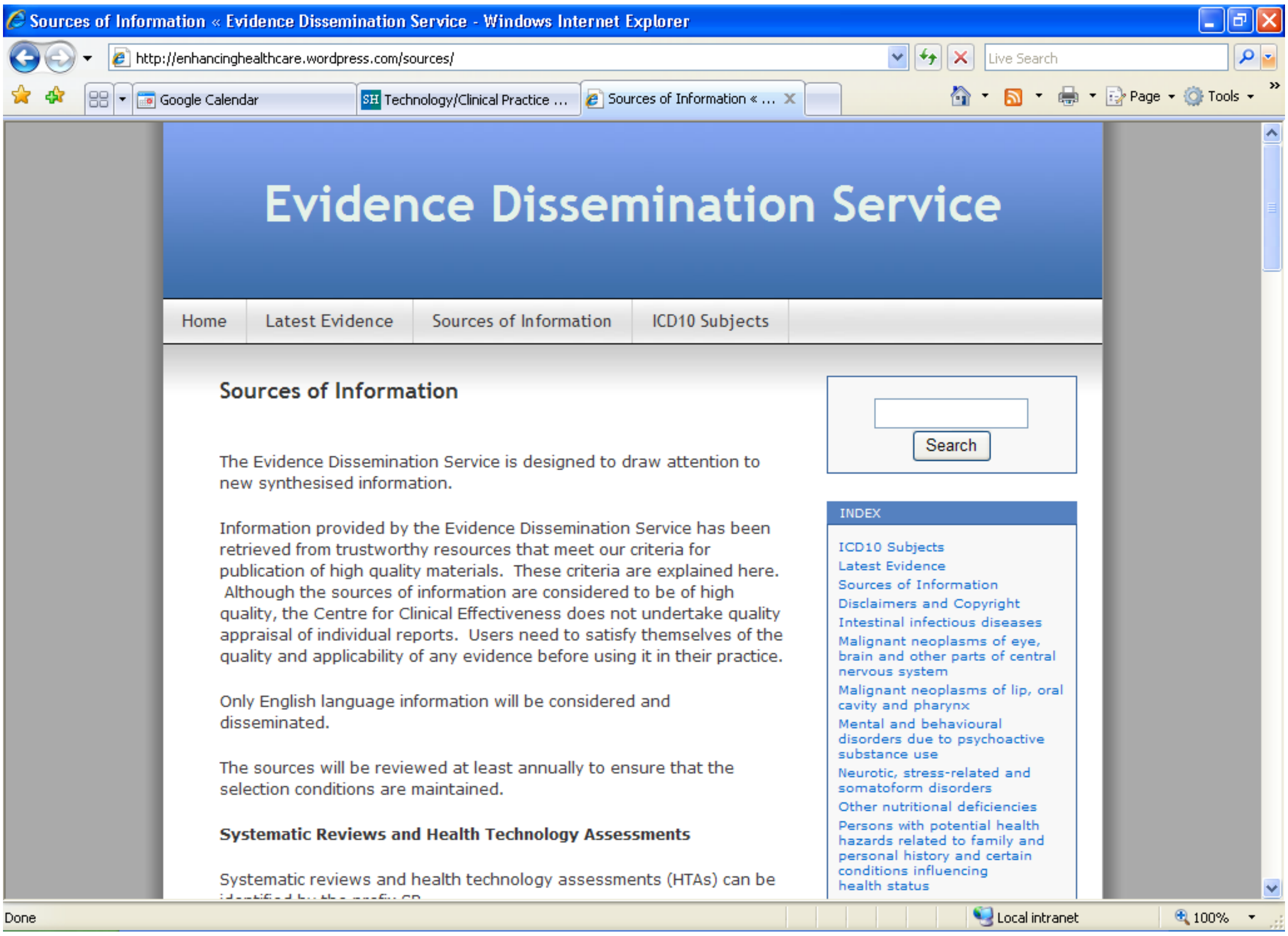
Sources of Information « Evidence Dissemination Service - Windows Internet Explorer
http://enhancinghealthcare.wordpress.com/sources/
Google Calendar
Technology/Clinical Practice ...
Sources of Information « ...
Evidence Dissemination Service
Home
Latest Evidence
Sources of Information
ICD10 Subjects
Sources of Information
The Evidence Dissemination Service is designed to draw attention to new synthesised information.
Information provided by the Evidence Dissemination Service has been retrieved from trustworthy resources that meet our criteria for publication of high quality materials. These criteria are explained here. Although the sources of information are considered to be of high quality, the Centre for Clinical Effectiveness does not undertake quality appraisal of individual reports. Users need to satisfy themselves of the quality and applicability of any evidence before using it in their practice.
Only English language information will be considered and disseminated.
The sources will be reviewed at least annually to ensure that the selection conditions are maintained.
Systematic Reviews and Health Technology Assessments
Systematic reviews and health technology assessments (HTAs) can be
Search
INDEX
ICD10 Subjects
Latest Evidence
Sources of Information
Disclaimers and Copyright
Intestinal infectious diseases
Malignant neoplasms of eye, brain and other parts of central nervous system
Malignant neoplasms of lip, oral cavity and pharynx
Mental and behavioural disorders due to psychoactive substance use
Neurotic, stress-related and somatoform disorders
Other nutritional deficiencies
Persons with potential health hazards related to family and personal history and certain conditions influencing health status
Done
Local intranet
100%

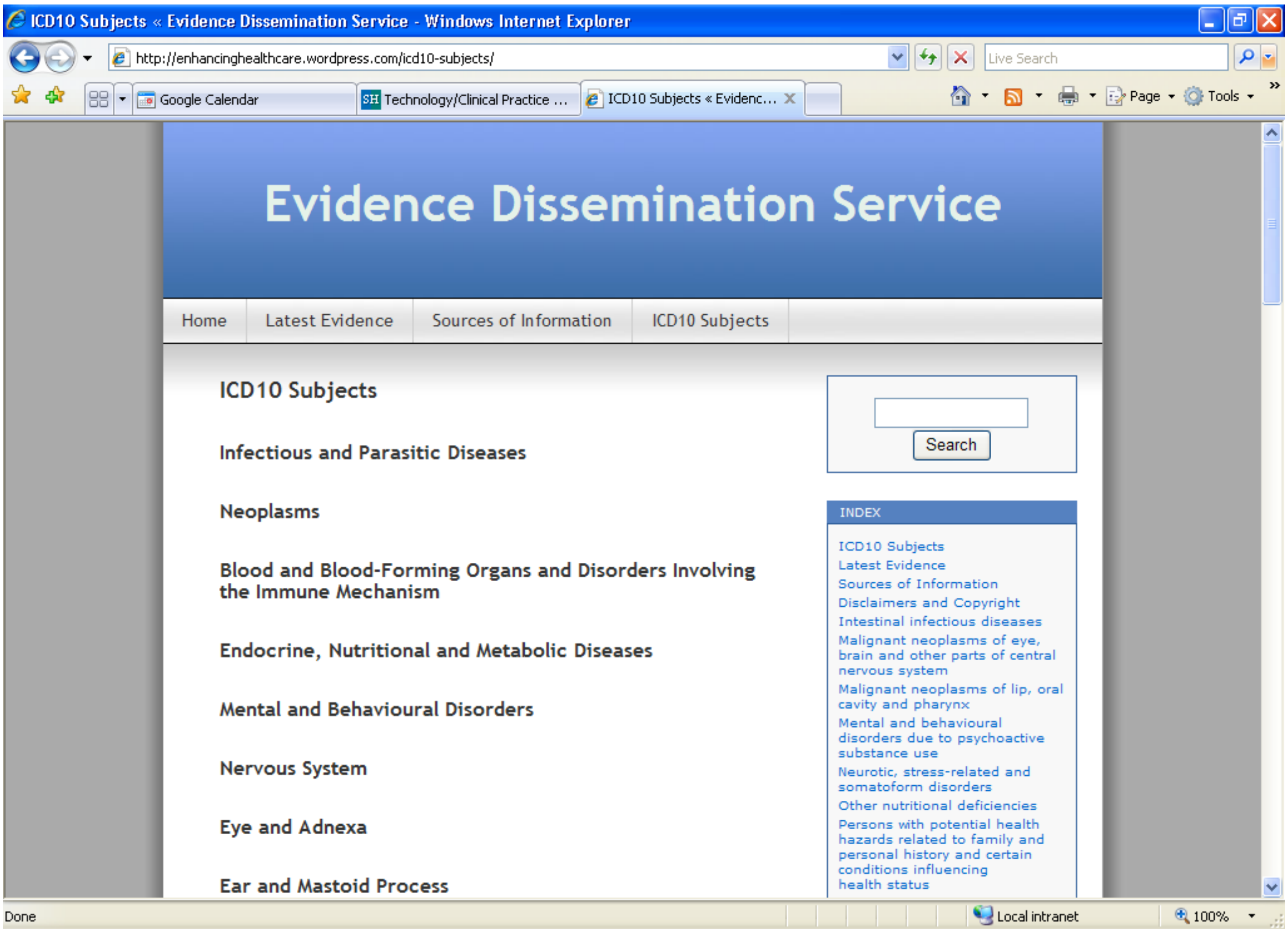
ICD10 Subjects « Evidence Dissemination Service - Windows Internet Explorer
http://enhancinghealthcare.wordpress.com/icd10-subjects/
Google Calendar
Technology/Clinical Practice ...
ICD10 Subjects « Evidenc...
Evidence Dissemination Service
Home
Latest Evidence
Sources of Information
ICD10 Subjects
ICD10 Subjects
Infectious and Parasitic Diseases
Neoplasms
Blood and Blood-Forming Organs and Disorders Involving the Immune Mechanism
Endocrine, Nutritional and Metabolic Diseases
Mental and Behavioural Disorders
Nervous System
Eye and Adnexa
Ear and Mastoid Process
Search
INDEX
ICD10 Subjects
Latest Evidence
Sources of Information
Disclaimers and Copyright
Intestinal infectious diseases
Malignant neoplasms of eye, brain and other parts of central nervous system
Malignant neoplasms of lip, oral cavity and pharynx
Mental and behavioural disorders due to psychoactive substance use
Neurotic, stress-related and somatoform disorders
Other nutritional deficiencies
Persons with potential health hazards related to family and personal history and certain conditions influencing health status
Done
Local intranet
100%

REVISED VERSION

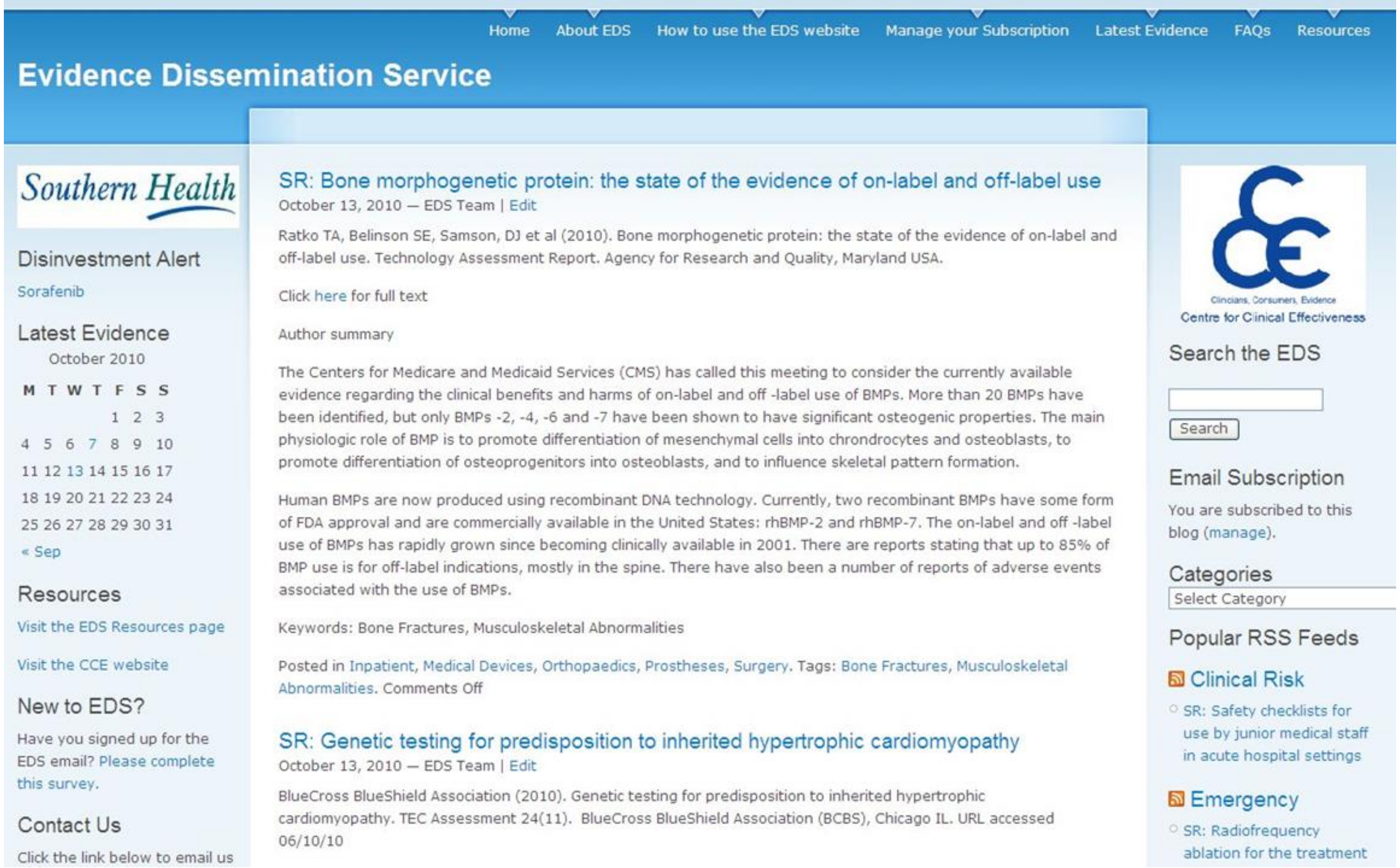

Home | About EDS | How to use the EDS website | Manage your Subscription | Latest Evidence | FAQs | Resources

Evidence Dissemination Service

Southern Health

Disinvestment Alert

Sorafenib

Latest Evidence

October 2010

M	T	W	T	F	S	S
				1	2	3
4	5	6	7	8	9	10
11	12	13	14	15	16	17
18	19	20	21	22	23	24
25	26	27	28	29	30	31

« Sep

Resources

Visit the EDS Resources page

Visit the CCE website

New to EDS?

Have you signed up for the EDS email? Please complete this survey.

Contact Us

Click the link below to email us

SR: Bone morphogenetic protein: the state of the evidence of on-label and off-label use

October 13, 2010 — EDS Team | Edit

Ratko TA, Belinson SE, Samson, DJ et al (2010). Bone morphogenetic protein: the state of the evidence of on-label and off-label use. Technology Assessment Report. Agency for Research and Quality, Maryland USA.

Click here for full text

Author summary

The Centers for Medicare and Medicaid Services (CMS) has called this meeting to consider the currently available evidence regarding the clinical benefits and harms of on-label and off -label use of BMPs. More than 20 BMPs have been identified, but only BMPs -2, -4, -6 and -7 have been shown to have significant osteogenic properties. The main physiologic role of BMP is to promote differentiation of mesenchymal cells into chrondrocytes and osteoblasts, to promote differentiation of osteoprogenitors into osteoblasts, and to influence skeletal pattern formation.

Human BMPs are now produced using recombinant DNA technology. Currently, two recombinant BMPs have some form of FDA approval and are commercially available in the United States: rhBMP-2 and rhBMP-7. The on-label and off -label use of BMPs has rapidly grown since becoming clinically available in 2001. There are reports stating that up to 85% of BMP use is for off-label indications, mostly in the spine. There have also been a number of reports of adverse events associated with the use of BMPs.

Keywords: Bone Fractures, Musculoskeletal Abnormalities

Posted in Inpatient, Medical Devices, Orthopaedics, Prostheses, Surgery. Tags: Bone Fractures, Musculoskeletal Abnormalities. Comments Off

SR: Genetic testing for predisposition to inherited hypertrophic cardiomyopathy

October 13, 2010 — EDS Team | Edit

BlueCross BlueShield Association (2010). Genetic testing for predisposition to inherited hypertrophic cardiomyopathy. TEC Assessment 24(11). BlueCross BlueShield Association (BCBS), Chicago IL. URL accessed 06/10/10

Clinicians, Consumers, Evidence

Centre for Clinical Effectiveness

Search the EDS

Search

Email Subscription

You are subscribed to this blog (manage).

Categories

Select Category

Popular RSS Feeds

Clinical Risk

- SR: Safety checklists for use by junior medical staff in acute hospital settings

Emergency

- SR: Radiofrequency ablation for the treatment

Section 13 Model 1 Example of EDS Email Alert

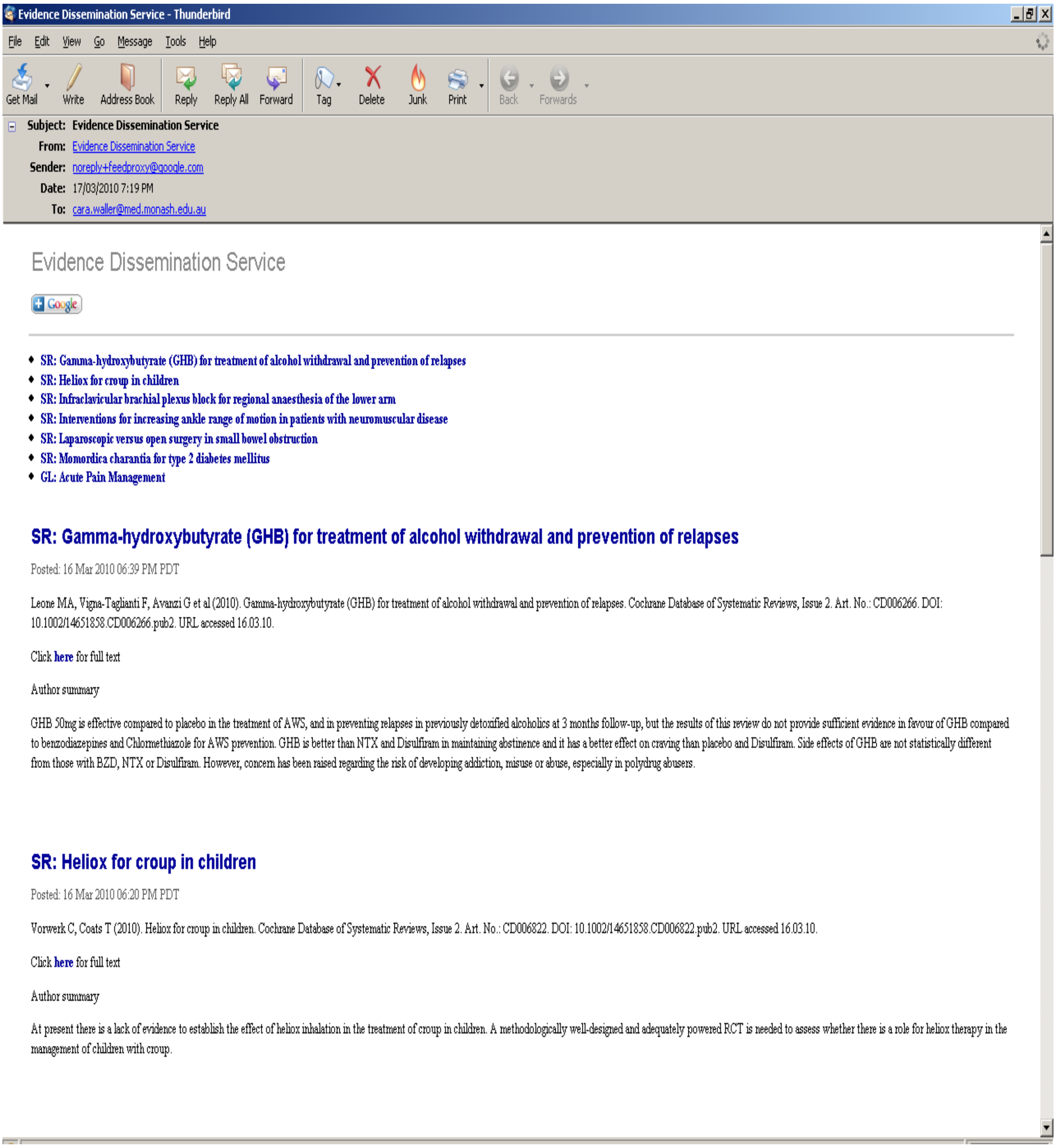

Evidence Dissemination Service - Thunderbird

File Edit View Go Message Tools Help

Get Mail Write Address Book Reply Reply All Forward Tag Delete Junk Print Back Forwards

Subject: Evidence Dissemination Service
From: Evidence Dissemination Service
Sender: noreply+feedproxy@google.com
Date: 17/03/2010 7:19 PM
To: cara.waller@med.monash.edu.au

Evidence Dissemination Service

+ Google

- SR: Gamma-hydroxybutyrate (GHB) for treatment of alcohol withdrawal and prevention of relapses
- SR: Heliox for croup in children
- SR: Infraclavicular brachial plexus block for regional anaesthesia of the lower arm
- SR: Interventions for increasing ankle range of motion in patients with neuromuscular disease
- SR: Laparoscopic versus open surgery in small bowel obstruction
- SR: Momordica charantia for type 2 diabetes mellitus
- GL: Acute Pain Management

SR: Gamma-hydroxybutyrate (GHB) for treatment of alcohol withdrawal and prevention of relapses

Posted: 16 Mar 2010 06:39 PM PDT

Leone MA, Vigna-Taglianti F, Avanzi G et al (2010). Gamma-hydroxybutyrate (GHB) for treatment of alcohol withdrawal and prevention of relapses. Cochrane Database of Systematic Reviews, Issue 2. Art. No.: CD006266. DOI: 10.1002/14651858.CD006266.pub2. URL accessed 16.03.10.

Click here for full text

Author summary

GHB 50mg is effective compared to placebo in the treatment of AWS, and in preventing relapses in previously detoxified alcoholics at 3 months follow-up, but the results of this review do not provide sufficient evidence in favour of GHB compared to benzodiazepines and Chlormethiazole for AWS prevention. GHB is better than NTX and Disulfiram in maintaining abstinence and it has a better effect on craving than placebo and Disulfiram. Side effects of GHB are not statistically different from those with BZD, NTX or Disulfiram. However, concern has been raised regarding the risk of developing addiction, misuse or abuse, especially in polydrug abusers.

SR: Heliox for croup in children

Posted: 16 Mar 2010 06:20 PM PDT

Vorwerk C, Coats T (2010). Heliox for croup in children. Cochrane Database of Systematic Reviews, Issue 2. Art. No.: CD006822. DOI: 10.1002/14651858.CD006822.pub2. URL accessed 16.03.10.

Click here for full text

Author summary

At present there is a lack of evidence to establish the effect of heliox inhalation in the treatment of croup in children. A methodologically well-designed and adequately powered RCT is needed to assess whether there is a role for heliox therapy in the management of children with croup.

Section 14 Draft tool for reporting use of evidence with completed examples

Clinical Area	Reference	Source	Evidence of benefit	Evidence of harm or no benefit	Lack of evidence	Applicability	Policy or procedure on this topic?	Policy or procedure consistent with evidence?	Quality	Change in practice needed?	If policy, procedure or local practice is not consistent with the evidence:<br>▪ What are the plans to implement change?<br>▪ What are the reasons for not implementing change?
Respiratory medicine	*Ward et al*	*Cochrane*	✓			☑ Good<br>☐ Limited<br>☐ Poor	☑ Yes<br>☐ No<br>☐ N/A	☑ Yes<br>☐ No<br>☐ N/A	☑ Good<br>☐ Limited<br>☐ Poor<br>☐ N/A	☐ Yes<br>☑ No<br>☐ N/A	*N/A*
	Kimber et al	*UK HTA*		✓		☐ Good<br>☑ Limited<br>☐ Poor	☑ Yes<br>☐ No<br>☐ N/A	☐ Yes<br>☑ No<br>☐ N/A	☑ Good<br>☐ Limited<br>☐ Poor<br>☐ N/A	☑ Yes<br>☐ No<br>☐ N/A	*The new drug will be implemented following an education program and introduction of revised local guidelines*
	Georgiou et al	*ASERNIPS*			✓	☑ Good<br>☐ Limited<br>☐ Poor	☐ Yes<br>☑ No<br>☐ N/A	☐ Yes<br>☐ No<br>☐ N/A	☑ Good<br>☐ Limited<br>☐ Poor<br>☐ N/A	☐ Yes<br>☑ No<br>☐ N/A	*In the absence of good evidence to retain or discontinue current practice, no changes will be made.*
						☐ Good<br>☐ Limited<br>☐ Poor	☐ Yes<br>☐ No<br>☐ N/A	☐ Yes<br>☐ No<br>☐ N/A	☐ Good<br>☐ Limited<br>☐ Poor<br>☐ N/A	☐ Yes<br>☐ No<br>☐ N/A	
						☐ Good<br>☐ Limited<br>☐ Poor	☐ Yes<br>☐ No<br>☐ N/A	☐ Yes<br>☐ No<br>☐ N/A	☐ Good<br>☐ Limited<br>☐ Poor<br>☐ N/A	☐ Yes<br>☐ No<br>☐ N/A	
						☐ Good<br>☐ Limited<br>☐ Poor	☐ Yes<br>☐ No<br>☐ N/A	☐ Yes<br>☐ No<br>☐ N/A	☐ Good<br>☐ Limited<br>☐ Poor<br>☐ N/A	☐ Yes<br>☐ No<br>☐ N/A	
						☐ Good<br>☐ Limited<br>☐ Poor	☐ Yes<br>☐ No<br>☐ N/A	☐ Yes<br>☐ No<br>☐ N/A	☐ Good<br>☐ Limited<br>☐ Poor<br>☐ N/A	☐ Yes<br>☐ No<br>☐ N/A	
						☐ Good<br>☐ Limited<br>☐ Poor	☐ Yes<br>☐ No<br>☐ N/A	☐ Yes<br>☐ No<br>☐ N/A	☐ Good<br>☐ Limited<br>☐ Poor	☐ Yes<br>☐ No<br>☐ N/A	

Section 15 Survey of staff enrolling in the EDS: Baseline data

All subscribers had been invited to complete a baseline survey regarding their use of evidence when they registered with the EDS. The findings were very similar to other surveys in this area [23, 24, 29, 41-43, 45-48, 57, 73-78] , including others at Monash Health [7]. Users consulted a range of sources to inform their decision-making and believed that EBDM resulted in the best clinical care.

Almost half (18/41) of the respondents found out about the EDS through the advertisement on the Monash Health Intranet, the others found out through the Chief Executive's Newsletter (8), referrals from colleagues (8), posters in the hospital (4), or other means (3). Most (33/45) reported that their role involved decision-making about introducing or changing use of TCPs.

All respondents 'sometimes', 'often' or 'always' included research evidence in their decision making. The internet, The Cochrane Library, and electronic databases were the most commonly used resources. Most respondents spent more than two hours searching for, assessing and appraising evidence for their decisions.

The majority of respondents agreed that Monash Health promoted the use of EBP (35/41) and facilitated employee's use of evidence in making decisions for TCPs (27/40); that EBP results in the best clinical care for their patients (37/40) and new medical technology requires rigorous evidence before introduction into clinical practice (37/42); that they have access to research findings in the workplace (32/41) and know where to get local Monash Health data for their decisions (23/41). Most (25/42) did not believe that EBP is difficult or that EBP takes too much time.

**Q1. How did you hear about the Evidence Dissemination Service**	
I saw it in the CEs newsletter	8
I saw it advertised in the front intranet page	18
I saw a poster in the hospital	4
I was referred by a colleague	8
I work at another health service and a Southern Health employee referred me	0
Other	3
Missing Answers	5
Total	46

Other, please specify: direct email notification, was a part of the pilot phase, electronic newsletter, Received MMC email

**Q2. What is your role at Southern Health?**	
Nursing	13
Allied Health	16
Medical	7
Other	10
Total	46

If Allied Health or Other, please specify: Physiotherapy 6, Occupational therapy 3, Strategic Planner/Manager SMICS 1, Pharmacy 2, Quality 1, Social work 1, Clinical psychologist 1, Speech pathology 1, Project Manager 2, Administrative 1, CCE 1

**Q3. In which Program do you work?**	
Continuing Care	8
Corporate Office	1
Medicine Program	7
Mental Health Program	2
Support Services	2
Specialty Program	4
Strategy, Performance and Planning	1
Surgery Program	2
Women's and Children's	5
Other	13
Missing Answers	1
Total	46

If Support Services or Other, please specify: Nursing & Midwifery Education & Strategy, Research/Theatre, SMICS, Critical Care, Imaging guided therapy, Care in Context - HARP Program, SACS, General medicine, Capital Projects, Pharmacy, Anaesthesia, Ambulatory and Community Care, CCE

**Q4. At which Southern Health site do you work?**	
Kingston	5
Moorabbin	6
Clayton	24
Dandenong	8
Casey	5
Cranbourne Integrated Care	2
Other	5
Total	46

Other, please specify: All sites, Pakenham, Yarraman, Middle South CCU, Berwick

**Q5. Does your role involve decision-making about introducing or changing use of TCPs?**	
Yes	33
No	12
Missing Answers	1
Total	46

**Q6. In your decision-making around TCPs, approximately how often do you include evidence from research?**	
Never	0
Rarely	0
Sometimes	10
Often	10
Always	12
Missing Answers	14
Total	46

Q7. How often do you use the following resources to find information about technologies?

	Never	Rarely	Sometimes	Often	Always	Total
Personal subscription to journals	6	1	9	10	4	30
Personal Subscriptions to email list services	9	4	8	3	3	27
Library hard copy journals	3	7	15	2	1	28
The Cochrane Library	2	2	13	8	3	28
Other electronic databases of research	0	5	5	10	9	29
Guideline websites	1	8	12	4	6	31
Internet	0	1	12	11	7	31
Other	3	0	4	1	1	9
Missing Answers Total						14

Other, please specify: senior clinical staff, trade displays / meetings, conferences, in-service, other hospital guidelines, conferences

Q8. During the last 6 months, what is the average time you spent including information from research in your decision-making? Please indicate how long, on average, you spent searching for, accessing and appraising this information?

	<30 minutes	30-60 minutes	60-90 minutes	90-120 minutes	>120 minutes	Total
Searching	4	6	6	1	15	32
Accessing	5	6	5	2	12	30
Appraising	3	6	8	1	12	30
Missing Answers						14

**Q9. Please rate your agreement with the following statements about evidence-based practice (EBP).**						
	**Strongly Disagree**	**Disagree**	**Agree**	**Strongly Agree**	**Don't know**	**Total**
Southern Health promotes the use of EBP	0	4	15	20	2	41
I believe EBP takes too much time	5	20	8	5	3	41
I know where to get Southern Health data for my decisions	3	14	19	4	1	41
I believe new medical technology does not require rigorous evidence to be introduced into clinical practice	19	18	1	4	0	42
I have access to research findings in my workplace	2	7	24	8	0	41
I believe EBP results in the best clinical care for patients	0	1	17	20	2	40
Southern Health facilitates employee's use of evidence in decision-making for TCP change	1	8	24	3	4	40
I believe EBP is difficult	3	22	12	2	3	42
I believe that in the absence of research evidence EBP can still be applied to decision-making about TCPs	3	14	14	3	7	41
Missing Answers						4

**Q10. Please indicate how frequently you do the following**						
	**Never**	**Rarely**	**Sometimes**	**Often**	**Always**	**Total**
I consult a range of information sources	4	0	10	18	8	40
I include the views of consumers in my decision-making	5	7	9	16	4	41
I use EBP guidelines or systematic reviews to change clinical practice where I work	4	1	15	20	2	42
I evaluate outcomes of practice change	2	7	13	15	4	41
I use evidence (research, clinical expertise, consumer preference) to change my clinical practice	2	0	15	18	7	42
Missing Answers						4

Section 16 Model 1 Pilot implementation and evaluation

Objective

To test and refine the features of Model 1 for use by individual decision-makers.

Characteristics of the pilot intervention

The scope, components and methods developed initially formed the pilot intervention.

Pilot activities were undertaken with a pragmatic sample of a range of individual decision-makers including executives, clinical program directors and senior managers from the SHARE Steering Committee and Technology/Clinical Practice Committee and clinical managers from one large multi-campus department.

Implementation strategies

EDS staff met with committee and department representatives to seek agreement in principle and then attended meetings to explain the service and obtain agreement from individuals. Personalised emails explaining the project and requirements of participants were sent to those who were not present at the meetings. The project team enrolled each of the designated staff members, but individuals were required to register to establish their account. An email invitation with information about the EDS, an embedded link for registration, and instructions on how to activate the link was sent to each participant.

Evaluation

Evaluation was conducted six months after implementation and included audit of website statistics, electronic survey of individual users, interview with EDS administrator, and reflections of the SHARE Steering Committee and project team. An additional survey was sent two months later to explore reasons for non-use of the EDS in the pilot sample. Details of the survey and interview questions, responses and project team observations are provided below and key messages are summarised.

Reach

Of the 73 individual decision-makers enrolled by the EDS team, 26 activated their email subscription and one created an RSS subscription. Due to problems determining the validity of email addresses it was difficult to define a denominator for this response. Medical staff frequently used personal email addresses and lists of committee members were not kept up-to-date; some may not have received the invitation and others may have left the organisation.

Users preferred the email to the website with email 'views' growing significantly over the pilot period while the website remained steady with relatively fewer 'views'.

While not officially in the evaluation period, in the eight months between the formal pilot and implementation of the revised EDS, subscription more than doubled to 64 participants with an average number of 100 visits to the site per month. The 'Home' page was the most frequently visited page of the site with the most recent systematic review being the most common destination for users.

Usefulness: User satisfaction

There were only eight responses to an online survey sent to individual participants. While this small number limits generalisability, the themes were very consistent and most respondents replied positively. Users were 'mostly' or 'completely' satisfied with the service. The website was viewed as 'easy' or 'very easy to use' and the amount of information on the website met user's needs. Email alerts were read and respondents reported accessing full text at least 'sometimes' and one person 'always'. One respondent questioned why there were not more publications in their area of expertise, suggesting that they misunderstood the nature of the service ie that it captured publications as they were published rather than selecting them by topic.

Usefulness: Service quality

All respondents rated the information as 'trustworthy', 'current' and 'coming from an authoritative source'. One respondent was unaware of the classification system, but the others reported that entries had been classified correctly. Two respondents suggested improvements, both related to identifying information relevant to users' specialty areas.

Use

Two individuals had used the information in making decisions about clinical practice. No one had used it for purchasing clinical consumables or capital equipment; although half thought that they would in the future.

The executives and senior managers reported that the information in the EDS alerts did not influence their decision-making because it was predominantly about clinical practice and their decisions were not. They observed that the different levels of management within the organisation required different types of information and proposed three levels:

1) Department heads and unit managers needed evidence for local policies and protocols related to clinical practice. 2) Program directors required evidence that informed their one to two year planning processes and was relevant to procedural aspects of the health service such as programs and service delivery as well as individual practitioners. 3) Executives and senior managers required information to inform three to five year forward planning that aligned with the organisation's strategic objectives. This resulted in addition of a category for 'Evidence-based policy and management advice' and development of criteria to identify high quality sources of this information; details in section on Definitions of evidence products above.

Implementation fidelity

The only modifications to the planned intervention were that some of the sources were not accessed during the pilot period. The intervention was implemented as planned. Barriers and enablers were identified and action taken. Almost all were related to technical issues in delivering the service.

Electronic survey of users

Evaluation Question	Method/Source	Results
**Reach**<br>What percentage of decision-making staff have subscribed to the EDS?	Audit of web statistics	Of the 73 SH staff signed-up by SHARE, 26 (35%) activated their email subscription.
What percentage of 'unsubscribed' users accessed the information through different means?	Audit of web statistics	Of the 47 staff who did not activate their email subscription, 1 person has an RSS subscription.
	Survey of users	Of the 8 respondents, 5 had activated email subscriptions, 1 did not use the service and 2 have subscribed to RSS updates.
Are there any patterns across sites, professions or programs, and what are the gaps?	Audit of web statistics	4/6 TCPC, 10/20 Therapeutics, 11/47 Diagnostic imaging (DI)<br>Gaps: Subscription rates in Therapeutics committee and DI low.
	Survey of users	5/8 Medical, 1 Allied Health, 1 Nursing and 1 Research<br>2 TCPC, 2 Therapeutics, 4 DI<br>All sites but Cranbourne Integrated Care were represented. All respondents spent time working at Clayton campus.<br>Gaps: No survey responses from Pharmacy
**Usefulness: Satisfaction**<br>What percentage of users is satisfied with the service?	Survey of users	6/7 reported being 'mostly' or 'completely' satisfied with the service. One person was not at all satisfied with the service.
What percentage of users read the email, accessed and read the website?	Survey of users	6/7 browsed the email, 4 'always', 2 'often'.<br>4/5 read the email in detail, 1 'always', 2 'often', 1 'sometimes'
What percentage of users followed links and read full-text articles	Survey of users	5/5 followed links from emails to full-text, 4 'sometimes', 1 'always'<br>2/4 'sometimes' followed links from the website to full text.
What percentage of users found the information received useful for decision-making?	Survey of users	2/7 had used the information in decision-making.
What percentage of users rate the amount of information as useful in decision-making.	Survey of users	Amount of information on the website: 5/7 said this met their needs, 1 commented this question was not applicable, 1 responded 'very few SRs on diagnostic imaging and no categorisation makes perusal inefficient'<br>Amount of information in the email: 6/6 reported this met their needs.
What percentage of users are satisfied with the frequency of email alerts/new information?	Survey of users	5/6 respondents wanted shorter emails more frequently (<30 updates in a weekly email)
Preference for classification of entries at the ICD 10AM level	Survey of users	5/7 respondents preferred more specific levels of ICD 10 headings
**Usefulness: Quality**<br>To what extent do users consider the information received as current or trustworthy? Information sources as authoritative?	Survey of users	7/7 rated information as 'trustworthy'<br>7/7 rated information as 'current<br>7/7 rated sources as 'coming from an authoritative source'
Trend of 'hits' to the website over time, as compared to subscription rates	Audit of web statistics	'Views' of emails grew by 600% (this could be affected by referral or by one or two people looking at the same thing).<br>The average number of 'views' to email 641 (range 21-1004)<br>Average number of 'clicks' on the website is 147/month, however, excluding July (set-up bias) , the average is 51 'clicks'/month

Number of entries classified under correct headings	Survey of users	5/6 reported that classifications were correct, 1 was unaware that entries were classified.
Ease of use	Survey of users	4/6 rated website as ‘easy’ or ‘very easy to use’, 2 did not access website.
Suggestions/comments	Survey of users	‘Little of relevance to diagnostic imaging. Most of the links did not work for me by opening the article when I clicked on it so could not read’<br>‘Needs to be tailored to the user's specialty and links need to work so that the information can actually be accessed. I was aware of SRs to do with diagnostic imaging (including one that I wrote!) which never came up in the emailed list. I am not sure how the selection process worked.’<br>‘The topics often seem esoteric’
**Implementation**<br>To what extent has the service been implemented as planned and what are the gaps?	Audit of implementation plan<br><br>Interview with administrator	There are a number of small things that have not been implemented due to the nature of the pilot (eg using the full list of original resources), however, the service is fully operational and implemented without any major changes to the implementation plan.<br>Unplanned modifications<br>▪ Applying narrower ICD 10 headings as a result of user feedback<br>▪ Excluding EUROSCAN from the list of resources<br>▪ Minor changes to taxonomy<br>▪ Changing broadcasts from fortnightly to weekly<br>Gaps in implementation<br>▪ Using the whole list of original resources<br>▪ Move the blog to a new domain name
What are the barriers and enablers to implementation?	Interview with administrator	Barriers<br>▪ Length of time to find, classify, upload and check evidence (approx. 3hrs)<br>▪ Slow computer<br>▪ Lack of clarity of Feedburner stats<br>▪ EDS staff use of website skewing data<br>Enablers<br>▪ Routine and streamlining process with templates etc<br>▪ Software is easy to learn and use<br>▪ Feedburner allows for timed email updates – means you can upload early and publish later
**Use**<br>What percentage of users have used information in decision-making?	Survey of users	3/7 had used the information in making decisions about ‘clinical practice’. No one had used the information for purchasing clinical consumables or capital equipment.
What percentage of users intend to use information in decision-making?	Survey of users	3/6 thought they would not use it in purchasing clinical consumables or capital equipment, 3/6 thought possibly<br>4/6 thought they would use it for clinical practice decisions, 2/6 thought possibly<br>4/6 thought they might possibly use it for other decision-making

Interview with EDS Administrator

To what extent has the EDS been implemented as planned?

There are a number of small things that have not been implemented due to the nature of the pilot, however, the service is fully operational and implemented without any major changes to the implementation plan.

The full list of resources to be checked has not been implemented yet as only a few of the resources were chosen for the pilot. These were those that met the quality criteria. This list needs to be revisited. New resources have emerged and will be added to the resource manual.

Have there been any unplanned modifications along the way and why or why not?

Second level ICD10 headings introduced half way into pilot. Depending on evaluation results, this will be retained and all entries under the top level headings removed.

Excluded EUROSCAN from the list of resources as it did not meet the quality criteria (sends out notifications without data – duplication of effort)

The taxonomy will always be in development. This is due to the nature of starting with existing classification systems not designed for this purpose. For example, the category for medicine from MeSH is too broad and lacks some specialisations. There is also duplication within the taxonomy which must be addressed.

The last month of the pilot was changed to a weekly email due to the amount of new evidence uploaded to the resources we are using over our holiday break

**Is there anything yet to be done that was in the plan?**
The full resource list has yet to be searched.<br>Move to a new blog with new domain name. The current EDS is on a personal account. Moving will allow any EDS team member to update it.<br>Reviewing original resources for quality. We have got it listed in the EDS as 'at least annually'. Maybe once every two/three years?<br>Did consider how long to keep the posts on the blog. It's not meant to be a repository – the email is main feature. Could remove after 6 months. Need to ask users or steering committee.
**What have been the main barriers and enablers to establishing and continuing the EDS?**
Barriers<br>Slowness of work computer necessitating work from home one day a week. This has been resolved with a new computer at work.<br>Incorporating this new task into work load has taken some time. Establishing a routine and developing a more streamlined process of gathering and updating the blog – once a barrier and now an enabler – hopefully. This has been facilitated by creating a template for broadcasts ie, keeping the headings in table format, learning that updates need to be entered in a certain order for SRs to go 1st on the blog.<br>Average load time is 3-4 hours.<br>Lack of definition from Feedburner re indicators and user statistics has meant that getting easy access to data to report back to the steering committee has been confusing and time consuming.<br>***Enablers***<br>Feedburner lets you decide what time the emails go out.<br>Can publish broadcasts without needing to do anything.<br>Setting up the blog was easy – the software is straightforward and easy to learn.
**What can be done to improve the service in the future?**
Creating a smooth workflow, eg. refining templates and routines, making the handbook.<br>Creating documents that aid others in updating the EDS when I am away. Training some staff in how to check resources, create and post broadcast. Also, training on how to use statistics on Wordpress and Feedburner. This can be done through using the practice blog.<br>Moving the existing EDS to a shared CCE Wordpress account (I've already set one up and that is where the practice blog is). This will enable any staff member to update it.
**What is needed for this to be a sustainable service in the future?**
Staff need to know how to take over if the administrator goes on leave. Because of the nature of the post, human editing is always needed. This won't be a completely automated process.<br>Instead of moving it to a new account, SHARE staff could create their own Wordpress account and I can add them to the administrator list for the EDS. We would then keep the current URL.<br>I think it might be a good idea to use the Wordpress email subscriber function instead of Feedburner. That way, all the statistics are in one place. The downside is that the Wordpress statistics for email subscribers don't show what email posts were most popular - Feedburner does. On the other hand, users subscribing through Wordpress can choose how often they want to get emails.<br>Setting aside a particular day and time to get evidence and loading it the next day really works.<br>If this was to become a state or national project there would need to be increased leadership and budget from another body. The software would need to be upgraded and the use of IT technician might be needed.<br>There might need to be some review process for quality assurance of the taxonomy with a clinical review every so often that checked a few posts to ensure categorisation was correct. This would be necessary for new people administrating the service.

Project team and Steering Committee observations

- Executives, Senior Managers and Program Directors required information about policy and management decisions which was not addressed in the predominantly clinical evidence provided from the sources previously identified.
- The need for users to identify publications that recommended ceasing or restricting a TCP for evidence of harm or lack of effect was noted.
- The Medical Admin trainee was unable to undertake the classification due to other commitments given greater priority. This was a limitation of the Medical Admin portfolio where crises requiring immediate attention occurred frequently.
- The EDS process was complex and only one staff member was familiar with all the requirements, creating problems when they were on leave.
- The pilot website had no branding which did not meet internal standards for Monash Health publications.
- Users reported a preference for shorter emails with fewer entries.
- Users were not certain about the purpose of EDS and why specific publications were not being disseminated. They were also not using the website search function.
- The free email software had significant limitations related to analysis of available statistics. The website software did not have an email subscription function at the start of the pilot but introduced it later.
- The initial taxonomy used first level ICD10 headings. This did not provide enough detail and half way through the pilot period this was changed to the second level. The change to second level headings within the limitations of the free software made the process of entering data very time intensive and created messy search results for users.
- The category of 'Professional Group' was thought to be too broad to be of real use, for example 'Medicine' was attached to almost every piece of evidence, and had considerable overlap with the 'Specialty' category.
- Citations in bulletins from EUROSCAN did not point to full text.

Follow-up electronic survey to explore non-use of EDS

1. Have you heard of the Evidence Dissemination Service (EDS) before today? Yes 5 No 4

2. If you have heard of the EDS, do you receive email updates or browse the website? Yes 5 No 0

3. If you ticked No above, please outline your reasons for not subscribing to email updates or browsing the website No responses

4. Please make suggestions for tailoring the service to better meet your needs in the future

Users

- Have no issues. Would love more renal/transplant issues but do find the other issues useful.
- When email is sent, it is very clear at a glance which units may be interested in article eg. Infectious Disease: Article A... Article B...
- Very good format. Maybe a wider range of topics; more on clinical drug trial reports
- Define source of information eg HS on email alert
- Unable to do this – I am not a staff member (Consumer representative). Some staff might like particular areas to be categorised or highlighted to enable quick access. I did not explore the possibilities here.

Non-users

- I imagine the EDS would provide links to new sources of evidence, references and summaries of noteworthy publications etc. Perhaps the EDS would set up a permanent link on the Clinicians Health Channel or directly on the intranet or send out a regular e-newsletter.

5. Although you may not have heard of the EDS or may not use it, please comment on how you imagine the EDS could be used to aid decision-making within the organisation more broadly.

Users

- Have already used information to pass on to head of unit which has been useful in decision-making for a trial we want to do
- First point of call prior to development of new clinical policy/procedure
- EDS has enormous potential. Sorry I can't be more helpful.

Non-users

- Good idea. Needs to be widely known about. Email updates are more likely to be effective than promoting web address. Specific topic updates on a regular basis may be helpful.
- Don't know what it is
- Would be very interested to receive the suggested ?monthly emails

Section 17 Model 1 Evaluation of full implementation

Evaluation was conducted ten months after implementation of Stage 1 and included audit of website statistics, survey of individual users, interviews and consultations with stakeholders, and reflections of the SHARE Steering Committee and project team.

The project team identified 46 of the 70 subscribers by their Monash Health email addresses (the others used anonymous personal emails) and surveys were sent by internal mail including an addressed return envelope and a chocolate incentive. A two week response time was stipulated.

The user survey had a 52% (24/46) response rate; all health professional groups and all campuses were represented. All three committee liaison representatives and two senior individual decision-makers participated in interviews.

Reach

Seventy subscribers enrolled during the evaluation period.

Most (20/24) survey respondents received email broadcasts and the others established personal RSS feeds. Although the EDS was set up for users to access information via email or RSS feed, it was encouraging to see the EDS accessed via the Monash Health intranet 182 times and 134 full text articles downloaded this way. It was difficult to interpret other available data as limitations with the free website software meant that 'user' and 'administrator' (EDS staff) traffic to the site could not be separated.

The Therapeutics Committee representative was a member of the SHARE team and received the full EDS email broadcasts; customised RSS feeds were developed to address the specific needs of the Medication Safety and Clinical Risk Committees.

Usefulness

Most (21/24) respondents were satisfied with the EDS and found the website, email broadcast or RSS feed met their needs 'fully' or 'partially'. The majority (17/19) of respondents found the categories useful and those that did not were not aware that this feature was available. Categories were used to quickly identify if the information was relevant to them and prevented them from looking at irrelevant information.

Committee representatives found that the format was *"...clear and relevant", "layout of the bulletins was easy to read", "summary of the findings was very good"* and *"volume of material is fine".*

The majority (22/24) of respondents found the content was 'current' and 'trustworthy', and 'useful' or 'partially useful'. Participants responded 'partially' or 'no' to any of the options because the information provided was not relevant to their area of clinical practice. The large volume of material was noted as a barrier to accessing the information contained in each broadcast. Six survey respondents provided suggestions for how the service could be improved; all related to making the categories more specific to avoid wasting time looking at irrelevant information.

Responses of committee representatives were mixed. Negative comments reflected the survey responses; *"A lot of information that wasn't particularly relevant", "too clinical"* and was *"rarely helpful or useful".* Positive findings included *"…providing the correct kind of information"* and *"hitting the mark of what you would expect from an Evidence Dissemination Service".*

Use

Less than half (9/24) of the survey respondents had used information from EDS in decision-making; examples of use included confirming current knowledge, ensuring knowledge is up-to-date, informing formulary decisions, passing information on to colleagues and using information in research. Only one respondent had used it for purchasing clinical consumables, none for purchasing clinical equipment, and nine for clinical practice change. However they were optimistic about the possibility of future use for purchasing clinical consumables or equipment, clinical practice change and other resource allocation decisions. The main reasons for not using the EDS information in decision-making were lack of time to read full articles and lack of relevance to the clinical setting.

Committee representatives reported that no information provided by the EDS was discussed at meetings held during the evaluation period. Further tailoring of customised RSS feeds was suggested by committees as a way to increase use, for example the Medication Safety Committee requested publications that demonstrated evidence of harm, evidence of reduction in risk of harm, and evidence regarding use of an effective alternative to a medication in current use. They were not interested in publications reporting lack of effect or insufficient evidence.

Two senior decision-makers responsible for organisation-wide portfolios were consulted regarding the draft reporting tool prior to implementation of stage 2. They were in agreement that the volume of work required to access the publication to identify whether it was relevant; then appraise it for quality, local applicability and consistency with existing policies and

procedures; take appropriate action and report using the proposed tool was too onerous and it was unlikely that model would be achievable.

Implementation fidelity

There was one major modification to the planned intervention. Following evaluation of stage 1, it was clear that this model would not meet the objectives and stage 2 was not undertaken.

All the proposed implementation activities for the participating committees were completed as planned and there were only minor changes to the plan for organisation-wide roll-out. Time constraints prevented the project team delivering demonstrations of the EDS in Monash Health public places and icons were not placed on all computers.

The barriers and enablers identified in the evaluation are discussed as factors influencing the processes and outcomes below and in Section 7d.

Participants

Individuals (survey): Forty-six paper based surveys were sent and 24 were returned.

Survey participant's role

	Total
Medical	4
Nursing	5
Allied Health	9
Pharmacy	2
Other	4
Total Participants	24

The four participants that selected 'Other' came from the Quality Unit, Corporate Office and Research Nursing, and one described their role as a project officer.

A large proportion of respondents were Allied Health staff. Due to the small numbers of overall respondents this may not be representative of the EDS user population.

Survey participant's site

	Total
Kingston	2
Moorabbin	4
Clayton	15
Dandenong	6
Casey	2
Cranbourne Integrated Care	1
Other	1
Total Participants	24

The majority of survey participants were located at Clayton.

Six participants listed multiple Southern Health sites

Survey participant's method of receipt of information from EDS

	Total
As an email (full bulletin)	20
As an RSS feed (selected topics delivered to inbox or browser)	4
Total Participants	24

The majority (83%) of participants received information from EDS as a full email bulletin.

Groups (interviews): The EDS engaged with three decision-making committees (Medication Safety, Clinical Risk and Therapeutics Committees). One committee representatives participated in a face to face interview, one an email interview, and one provided feedback directly as they were also a member of the EDS team.

Reach

The EDS attracted 70 active subscribers during the evaluation period.

The statistics generated by Wordpress.com suggested that users accessed EDS via the Intranet 182 times. The most clicked links included the resource page (19 clicks), the CCE internet homepage (18 clicks) and the CCE email query link (11 clicks). A total of 134 full text articles were accessed via the EDS website.

Access to the EDS website was variable over the 10 months of activity. Although the EDS was set up for users to access information via an email or RSS feed it is encouraging to see that users were still visiting the site. The reasons for the peaks and troughs in access are unclear. A potential explanation for the high peak in the first month may be due to access by the project team to sort through initial teething problems, or extra interest by new users which was not sustained. Limitations with the software meant that we could not separate 'users' from 'the administrator' (CCE staff).

All three committees participated.

Usefulness

Satisfaction

Survey participants were asked to rate their overall satisfaction with the EDS. The majority (21/24) of participants were either 'partially' or 'very satisfied' with the EDS overall

Content

Survey participants were asked whether the amount of information provided by the EDS met their needs; 8/24 found the website content useful, 12/24 found the email alert useful and 2/24 found the RSS feed content useful. The main message from the participant's feedback reflects that there was a significant amount of non-specific information being sent to users. This results in a time-consuming activity for participants who trawl through each piece of evidence.

Survey participant's responses to amount of information provided by EDS meeting their needs

	Yes	Partially	No	N/A	Missing	Total
Website	8	3	0	6	7	**24**
Email (full bulletin)	12	5	2	0	5	**24**
RSS feed (delivered to inbox or browser)	2	3	0	12	7	**24**

Participants who answered 'partial' or 'no' provided the following feedback:

- "Probably too much irrelevant stuff (I am not sure whether I selected the correct options when I subscribed)"
- "The amount of emails I receive is quite large and trawling through them is time consuming. I don't have much time to attend to articles"
- "Would be good to group into medical, nursing, allied health specific info if relevant"
- "A lot of irrelevant information - not much specific topical info"
- "Very little information provided for medication safety that was relevant, however this may be a reflection of the lack of evidence for medication safety related topics"
- "It isn't specific like the BMJ Evidence email service. I do not want to know about articles that are not relevant to my practice"
- "So much unfiltered and irrelevant"

Committee representatives were asked about usefulness of the content of EDS alerts. The responses were:

- "A lot of information that wasn't particularly relevant...I just don't need RCTs but other published articles are also helpful".
- "Too clinical" and was "rarely helpful or useful"
- "Providing the correct kind of information" and was "hitting the mark of what you would expect from an evidence dissemination service".

Format

The EDS categorises information by healthcare setting, type of technology, professional specialty and special interest groups. 17/19 respondents found the EDS categories useful. Participants found that the categories helped them to quickly realise if the information was relevant to them and prevented them from looking at irrelevant information. The reason they did not find the categorisation of evidence useful is because they did not notice the feature.

Survey participant's responses to usefulness of EDS categories

	Yes	No	Missing	Total
Usefulness of EDS categories	17	2	5	24

The following explanations were provided for participants finding the categories useful:

- "Although would prefer more specific ones"
- "It helps me quickly realise what info is useful to me"
- "Allows quick browsing"
- "Generic covered most areas"
- "Useful so you don't have to sort through irrelevant information"
- "I focus more on the topic presented, not the category"
- "But would like more around current policy environment such as food and nutrition interventions, medicare locals"

The following explanations were provided for participants not finding the categories useful:

- "Probably would be useful - wasn't aware of this feature"
- "I have never noticed the grouping before"
- "Need to be more specific"

Committee representatives were also asked to respond to the format of the EDS alerts. The responses were:

- "The layout of the alerts was easy to read and OK" but "the abbreviations were a bit confusing eg SR".
- "The summary of findings was very good" and the "volume of material...fine".
- "The format is clear and relevant".

Quality

The majority (22/24) of respondents found the information provided by EDS to be current, trustworthy and useful or partially useful. Participants who responded 'partially' or 'no' to any one of the options agreed that the information provided was not relevant to their practice.

- "Too much irrelevant information"
- "A lot of the information I receive is of little or no use to my practice. Although some items are quite interesting"
- "It isn't specific like the BMJ Evidence email service. I do not want to know about articles that are not relevant to my practice."

Survey participant's responses to consideration of current, trustworthy and useful

	Yes	Partially	No	Missing Data	Total
Current	22	1	0	1	24
Trustworthy	22	1	0	1	24
Useful	11	11	1	1	24

Recommended improvements

Six of the 24 survey respondents provided the following suggestions for how the service could be improved:

- "As discussed, further alerts about current health policy environment or health interventions"
- "More categories to be able to focus in on relevant information"
- "Provision of services related to a specific area eg can you please provide relevant research/evidence related to...would be most helpful"
- "Make it clearer with regards to allied health related content"
- "Categories more specific - although realised I should check the website which I will do"
- "It might just be me - need to refine the subscription to make things more relevant"

Program Use

Accessing EDS content

The majority of survey respondents 'always', 'often' or 'sometimes' browsed email alerts or RSS feeds for interesting items (22/23) and followed links to full-text for items of interest (17/20). A considerable proportion of survey participants did not know they could browse the EDS website for interesting items (11/24), follow links to full-text for items of interest from the website (9/24) or search the website by categories (11/24).

Survey participant's use of the email alerts and RSS feeds

	Always	Often	Sometimes	Never	Missing	Total
I browse email alerts or RSS feeds for interesting items	6	9	7	1	1	**24**
I follow links to full-text for items of interest	1	4	12	3	4	**24**

Survey participant's use of the website

	Yes	No, I didn't want to	No, I didn't know I could	Missing	Total
I browse the website for interesting items	5	5	11	3	24
I follow links to full-text for items of interest	10	2	9	3	24
I search the website by categories	2	7	11	4	24

The three committee representatives looked at the EDS alerts they received and screened them for relevance to their respective committees.

Use of EDS in decision-making

Less than half (9/24) of the participants had used EDS to guide decision-making; these included formulary decisions, to confirm ideas about certain interventions or to update clinical knowledge.

The main reasons participants had not used EDS to guide decision-making (15/24) was because they had not had time to read the full articles or there had not yet been any relevant information to their clinical setting.

Survey participant's use of information received from EDS in decision-making

	Yes	No	Missing Data	Total
Use in decision-making	9	15	0	24

The following comments were provided by participants regarding how they used EDS in decision-making:

- "Confirm ideas/interventions"
- "Formulary decisions"
- "Only by passing info to medical staff"
- "I ensure my clinical knowledge is up to date and look for further or stronger evidence in key areas"
- "Have used info to add to other research"

The following comments were provided by participants regarding why they did not use EDS in decision-making:

- "Often don't have time to explore further"
- "I often don't have time to read the full articles but if I did it would affect decision-making"
- "Not as yet, I have only been a recent subscriber"
- "I haven't been able to obtain any relevant information to assist decision-making yet"
- "Nothing has been appropriate for me in decision-making but I have seen info which would be useful to others"

Committee representatives reported that no information from the EDS had been discussed and acted upon at meetings.

The Medication Safety Committee representative noticed that the evidence in the alerts was rarely helpful or useful for their committee. They found the evidence was too clinical for their area of interest and did not match their committee's areas of concern. For this reason no information was presented to the committee.

The Clinical Risk Committee representative noticed that there was a lot of information that was not particularly relevant however they were happy to screen and choose areas that were of interest to the committee. This representative had had problems receiving customised alerts and therefore found it difficult to find any relevant information to pass on.

The Therapeutics Committee did not discuss any material at their meeting because the representative and chair of the committee decided that no information was relevant.

Use in decision-making for resource allocation

Only one respondent had used EDS to inform decision-making for purchasing clinical consumables, no one reported using it for purchasing clinical equipment, however 9 had done so for clinical practice change. One participant commented that their non-use was related to the fact that their area of practice was not represented often and that there was a lot of medical and drug information that did not apply to them.

Survey participant's use of EDS in decision-making for resource allocation

	Yes	No	Missing Data	Total
Purchasing clinical consumables	1	22	1	24
Purchasing clinical equipment	0	23	1	24
Clinical practice change	9	15	0	24
Other	0	12	12	24

Half (12/24) of respondents felt they would possibly or definitely use EDS to inform decision-making for purchasing clinical consumables and for purchasing clinical equipment. The majority (22/24) said they would use EDS to guide decision-making for clinical practice change in the future. Reasons given for future use or non-use included the following:

- “I am not involved in clinical practice”
- “Not for me but maybe for others”
- “Would always pass on relevant info to relevant medical staff”
- “Possibly clinical practice change if I have time to read and evaluate the evidence”
- “I don't have control over any budget/purchasing”
- “Depends on the information available”

Survey participant’s future use of EDS in decision-making for resource allocation in the future

	Yes	Possibly	No	Missing Data	Total
Purchasing clinical consumables	2	10	11	1	24
Purchasing clinical equipment	0	12	11	1	24
Clinical practice change	5	17	2	0	24
Other	1	5	3	15	24

It was interesting to note that, of the participants who had answered ‘no’ (22/24) to using the EDS in decision-making for ‘purchasing clinical consumables’, 11/22 said they would, or possibly would, use it in the future. Participants who answered ‘no’ (23/24) to using the EDS in decision-making for ‘purchasing clinical equipment’, 12/23 said they would possibly use it in the future. Participants who answered ‘no’ (15/24) to using the EDS in decision-making for ‘clinical practice change’, 13/15 said they would, or possibly would, use it in the future.

Implementation

Implementation activities were undertaken for two separate target audiences, all Monash Health staff and the targeted committees. The EDS Manager was responsible for coordinating implementation activities and other EDS project members were responsible for providing technical support to users.

The majority (4/6) of the activities for implementation across the organisation were undertaken as planned. Advertisements were included online and in print and were promoted on the Monash Health Intranet and specific staff portals. Due to time restraints the project team were unable to undertake demonstrations of the EDS for specific groups or in Southern Health public places.

Methods and success achieved for organisation-wide implementation

Proposal	Achieved	Outcome
Place ad in CE’s newsletter	✓	One ad was placed when the EDS disseminated its first alert.
Adverts disseminated in the form of flyers via Email, eBoards, Notice boards	✓	Flyers were placed across all campuses in public areas as well as within departments.
Demonstrations of EDS (public place or for specific groups eg registrar meetings)	x	Time restrictions meant that this activity was not undertaken.
Launch newly modified and updated website across the SH intranet site (also include message about brief survey)	✓	A logo and brief description was posted on the front page of the Southern Health intranet as well as permanently placed in the side bar.
Investigate the possibility of putting an icon on all SH computers	x	More appropriate locations were identified compared to all Southern Health computers, therefore this activity was not undertaken.
Investigate the possibility of adding ‘hotlinks to specific user sites, such as Pharmacy website, Medical Staff Portal, Allied Health Staff Portal, Library website, CCE website	✓	Links to the EDS were included on the Emergency, Pharmacy and Allied Health portals. Because the EDS was only for internal dissemination it was not included on the CCE website.

All activities for implementation with the target committees were undertaken. The EDS Manager met with the three committee representatives and discussed all elements of the EDS with them. Further work could have been undertaken to identify potential barriers and enablers to using the EDS with the committee representatives.

Methods and success achieved for committee implementation

Proposal	Achieved	Outcome
Liaise with committee contacts and identify barriers and enablers to using EDS	✓	Undertaken
Establish best communication processes for committee representatives	✓	Undertaken
Discuss the details about EDS with committee representatives	✓	Undertaken
Discuss possible strategies for using EDS in committee meetings	✓	The EDS became a standing item on all agendas of committee's engaged to use EDS.
Establish most appropriate time to introduce EDS to committees	✓	Undertaken

Project team and Steering Committee observations

Relevance of material

- Main message from participant's feedback was that a significant amount of irrelevant information was being sent to users.
- The time to develop and disseminate this service should be considered especially if the information is not relevant to recipients.
- One user has suggested they like the information delivered by BMJ Evidence Updates because the information is relevant to their specialty. Although this is a different resource to EDS, we should consider relevance of information as a priority.

Use of EDS

- Users were not always clear about use of EDS
- Almost all chose email and received everything then complained about the volume they received. They could have RSS feeds on their areas of interest
- Demonstrations for how to use the EDS should be considered in the next phase of development.
- Particular attention should be made to demonstrate to users how to receive RSS feeds.
- The EDS team should investigate other platforms to run the EDS. At the moment only one specialty area or the full alert can be selected for users to receive information. Users would like to be able to select more than one specialty to ensure emails are specific to their areas of interest and Wordpress.com does not allow this function.

Resource use – time and skills

- Participants: Too much, too busy, not all relevant, things they knew already, not new evidence or SR finding lack of evidence, not important, etc
- KB team: too many publications, can't process all available
- If we had followed our plan of getting department heads to do all the follow up re local policies and protocols etc this would have been very time consuming, particularly for evidence that was not very important, and which may already be documented practice for the organisation.
- We proposed that decision-makers appraise the information, check for policies and protocols, and report. Decision-makers don't want any additional work, we know they don't have the time and skills to appraise – we could do that for them

Not achieving aims

- Systematically disseminated but not systematically used
- Not integrated into other decision-making processes – we tried with monthly reporting but too onerous
- Not accountable or transparent
- Those who did receive it were not always the appropriate decision-makers
- Can't be sure practice is evidence-based
- Individuals may or may not have changed practice or their own practice may have been consistent so they didn't need to change. SHARE was about a systemic approach, integrating new decision-making systems and processes into existing infrastructure for organisational impact. We needed a process that addressed organisational practice not individual practice. Needed to integrate it into existing processes for determining organisational practice.
- We had determined designated groups and individuals who made decisions regarding resource allocation for TCPs in previous project, targeting to them would be better use of resources and more likely to achieve aims
- We are not following the evidence regarding desirable characteristics of evidence products, we don't have targeted messages

Section 18 Systematic Review Appraisal

Assessment criteria (for CCE use only)

Quality assessment category*	Study Validity Criteria	Outcome	Assessment criteria
**(A) Conflicts of interest**	Is it clear that there were no conflicts of interest in the writing or funding of this review?	Yes; No; Not reported	✓✓ = yes<br>✗ = no<br>? = not reported
**(B) Appropriate study design**	Does the review have a clearly focused question?	Yes; Partial; No	✓✓ = 2 'yes';<br>✓ = 1 'yes' or 2 'partials'<br>✗ = 2 'no' or 1 partial + 1 'no'
	Is a systematic review the appropriate method to answer the question?	Yes; Partial; No	
**(C) Study selection**	Does the review have specified inclusion/exclusion criteria?	Yes; Partial; No	✓✓ = 3 'yes'<br>✓ = 'yes' for search strategy + any other answer<br>✗ = 3 'no' or 'no' for search strategy + any other answer
	If there were specified inclusion/ exclusion criteria, were these appropriate?	Yes; Partial; No; N/A	
	Does the review document a comprehensive search strategy?	Yes; Partial; No; Not reported	
**(D) Allocation and blinding**	Were reviewers blind to authors, institutions and affiliation?	Yes; Partial; No; Not reported	✓✓= yes<br>✓ = partial<br>✗ = no<br>? = not reported
**(E) Data collection**	Were 2 or more independent reviewers used for: application of inclusion criteria?	Yes; Partial; No; Not reported	✓✓ = 3 'yes'<br>✓ = any 1 or 2 'yes' + any other answer<br>✗ = all 'no'<br>? = 3 'not reported'
	Were 2 or more independent reviewers used for: extraction of data?	Yes; Partial; No; Not reported	
	Were 2 or more independent reviewers used for: appraisal of study quality?	Yes; Partial; No; Not reported	
**(F) Attributable to intervention**	Were the strengths and limitations of included studies and potential impact on the results discussed?	Yes; Partial; No; Not reported	✓✓ = 2 'yes'<br>✓ = 2 'partial' or 1 'yes + any other answer<br>✗ = 2 'no'<br>? = 2 'not reported'
	Was the validity of included trials appraised using appropriate criteria?	Yes; Partial; No; Not reported	
**(G) Appropriate analysis**	Is there a summary of the results of individual studies?	Yes; Partial; No	✓✓ = 3 'yes' or 1 'yes' for summary + 2 'N/A'<br>✓ = 1 'no' for any criteria + any other answer<br>✗ = 3 'no' or 1 'no' for summary + 2 'N/A'
	If meta-analysis were conducted, was it reasonable to do so?	Yes; Partial; No; N/A	
	If meta-analysis were conducted, was it done appropriately?	Yes; Partial; No; Not reported; N/A	
	Other		
	What is the overall risk of bias?	Low; Moderate; High; Insufficient information	

Model 2 (Pilot)

Tick boxes were used in pilot. The details below are an example of information contained in an Evidence Bulletin

Quality of evidence

Study	Disease area	Quality assessment*						
		Conflicts of interest	Appropriate study design	Participant selection	Allocation and blinding	Data collection	Attributable to intervention	Appropriate analysis
Smith et al 2009	Diabetes	?	✓✓	✓✓	✓	✓	✓✓	✓✓

*Quality assessment: ✓✓ *criterion met,* ✓ *criterion partially met,* ✗ *criterion not met,? unclear from the information provided*

Application of evidence

☐ **Use with confidence: Low Risk of Bias** (All of the quality criteria have been fulfilled or where criteria have not been fulfilled it is very unlikely the conclusions of the study would be affected)

☒ **Use with consideration of limitations: Moderate Risk of Bias** (Some of the criteria have been fulfilled and those criteria that have not been fulfilled may affect the conclusions of the study)

☐ **Use with caution: High Risk of Bias** (Few or no criteria fulfilled or the conclusions of the study are likely or very likely to be affected) or Insufficient information (not enough information provided to be able to determine risk of bias)

Consistency with Southern Health documented practice

☐ Southern Health policies or procedures appear to be consistent with the evidence

☐ Southern Health policies or procedures do not appear to be consistent with the evidence

☐ No Southern Health policies or procedures on this topic were identified

Model 2 (Full implementation)

Drop-down boxes were added to the template so that only findings applicable to this publication are reported. The text incorporates the implications of bias in application of the evidence.

Quality of evidence

Quality of this Systematic Review or Health Technology Assessment

CCE staff have appraised the methods used in this publication and found the risk of bias to be **LOW**. This means that you can use the findings of the review with confidence as all of the quality criteria have been fulfilled or where criteria have not been fulfilled it is very unlikely the conclusions of the study would be affected.

Quality of the *evidence contained* in this Systematic Review or Health Technology Assessment

The review authors have appraised the available evidence and found it to be **Level I Evidence** (a systematic review of Level II studies) of **high quality**.

Consistency with Southern Health documented practice

Southern Health policies or procedures appear to be consistent with the evidence

Southern Health

Technology/Clinical Practice Committee Evidence Bulletin

This bulletin is part of a process to ensure that Southern Health practice is consistent with current evidence. Your response is required by the date below. You can find more information about this process on the TCPC website.

The publication below indicates evidence of Choose an item.[10] related to

Responses related to evidence of Choose an item.[11] are required within Choose an item. [12]

Please complete and return this bulletin to marie.garrubba@monash.edu by Click here to enter a date.

Bibliographic Source

<LINK>

Author's Conclusion

Applicability to Southern Health

**Patient / Population**	
**N**	
**Setting**	
**Intervention**	
**Comparison**	
**Outcomes**	
**Inclusion Criteria**	
**Exclusion Criteria**	

Quality of Evidence

Quality of this Systematic Review or Health Technology Assessment

CCE staff have appraised the methods used in this publication and found the risk of bias to be Choose an item.[13] This means that you can use the findings of the review with Choose an item.[14]

Quality of the *evidence contained* in this Systematic Review or Health Technology Assessment

The review authors have appraised the available evidence and found it to consist of Choose an item.[15] The available evidence included in the review is of Choose an item.[16]

---

[10] Harm, Clinical Effectiveness, Cost Effectiveness, Technical Effectiveness, Lack of Effect

[11] Harm, Clinical Effectiveness, Cost Effectiveness, Technical Effectiveness, Lack of Effect

[12] 1 month, 3 months, 6 months

[13] Low, Moderate, High

[14] …confidence as all of the quality criteria have been fulfilled or where criteria have not been fulfilled it is very unlikely the conclusions of the study would be affected.

…consideration of limitations as some of the criteria have been fulfilled and those criteria that have not been fulfilled may affect the conclusions of the study.

… caution as few or no criteria fulfilled or the conclusions of the study are likely or very likely to be affected.

[15] Level I Evidence (a systematic review of level II studies)

Level II Evidence (a randomised controlled trial)

Level III-1 Evidence (a pseudo-randomised controlled trial)

Level III-2 Evidence (a comparative study with concurrent controls; non-randomised experimental trial, cohort study, case-control study, interrupted time series with a control group)

Level III-3 Evidence (a comparative study without concurrent controls; historical control study, two or more single arm studies, interrupted time series without a parallel control group)

Level IV Evidence (a case series with either post-test or pre-test/post-test outcomes)

Consistency with Southern Health documented practice

Choose an item. [17]

Response

- Click once on the shaded box to select the appropriate response
- Click once on the shaded rectangle to provide a typed comment

Practice at Southern Health (please select one response only, tick the box and provide relevant details)

☐ Not applicable at Southern Health *eg the patient group is not treated at Southern Health* (please explain)

☐ Practice is consistent with the evidence (please add comments if relevant)

☐ Practice is not consistent with the evidence for a good reason (please explain)

☐ Practice was not consistent with the evidence, remedial action has been undertaken and completed (please explain)

☐ Practice is not consistent with the evidence and remedial action has been commenced/planned (please explain)

Communication

Should this information be disseminated more widely? If so, to whom?

Other comments

Feedback

This is a pilot of new processes being implemented by the Technology Clinical Practice Committee and the Centre for Clinical Effectiveness Evidence Dissemination Service.

We would appreciate any comments regarding what works, what doesn't work and how we can improve the process.

**Name:**			
**Position:**		**Date:**	

Thank you

---

[16] Low quality, Moderate quality, High quality, Variable quality

[17] Southern Health policies or procedures appear to be consistent with the evidence

Southern Health policies or procedures do not appear to be consistent with the evidence

No Southern Health policies or procedures on this topic were identified

Southern Health

Technology/Clinical Practice Committee Evidence Bulletin_164

This bulletin is part of a process to ensure that Southern Health practice is consistent with current evidence. Your response is required by the date below. You can find more information about this process on the TCPC website.

The publication below indicates evidence of **Potential HARM (due to significant adverse events/side effects but lack of evidence of effectiveness)** related to Tricyclic antidepressants for autism spectrum disorders (ASD) in children and adolescents.

Responses related to evidence of **Potential HARM** are required within **ONE month.**

Please complete and return this bulletin to marie.garrubba@monash.edu by **11 June 2012**

Bibliographic Source

Hurwitz R, Blackmore R, Hazell P, Williams K, Woolfenden S. Tricyclic antidepressants for autism spectrum disorders (ASD) in children and adolescents. Cochrane Database of Systematic Reviews 2012, Issue 3. Art. No.: CD008372. DOI:10.1002/14651858.CD008372.pub2. http://onlinelibrary.wiley.com/doi/10.1002/14651858.CD008372.pub2/pdf

Author's Conclusion

Clinicians considering the use of TCAs need to be aware of the limited and conflicting evidence of effect and the side effect profile when discussing this treatment option with people who have ASD and their carers. Further research is required before TCAs can be recommended for treatment of individuals with ASD.

Applicability to Southern Health

**Patient / Population**	Inclusion was limited to children and adolescents (birth to 18 years of age) with a diagnosis of an autism spectrum disorder (ASD), using a standardised diagnostic instrument (for example, ADOS, ADI-R, DISCO, CARS) or using established diagnostic criteria as defined by DSM-IV or ICD-10, that is Pervasive Developmental Disorder, excluding Rett Syndrome and Childhood Disintegrative Disorder.
**N**	3 studies – number of participants unclear
**Setting**	Outpatient setting
**Intervention**	Any oral tricyclic antidepressants, regardless of dosage used, duration of use or frequency of administration.<br>Tricyclic antidepressants include amitriptyline (amitriptyline hydrochloride), amoxapine, clomipramine (clomipramine hydrochloride), dothiepin (dosulepin hydrochloride or dothiepin hydrochloride), doxepin, imipramine (imipramine hydrochloride), iofepramine, nortriptyline, trimipramine, desipramine, florpiramine, dibenzepin, iprindole, protriptyline and modified tricyclic antidepressants such as tianeptine.
**Comparison**	Placebo
**Outcomes**	**Primary outcomes**<br>• Core symptoms of autism, for example, impairments in communication, reciprocal social interaction and behavioural problems, such as repetitive behaviours and rituals, obsessional behaviour and stereotypy.<br>• Non-core symptoms, including challenging behaviours, sleep disturbance and aggression.<br>• Comorbidities, including depression and anxiety.<br>• Adverse effects.<br>**Secondary outcomes**<br>• Parental, child or family quality of life.<br>• Parental or family stress.<br>We planned to examine short-term (up to three months), medium term (three to 12months) and long-term (greater than 12 months) outcomes if the data were available.<br>We used the primary and secondary outcomes to populate the 'Summary of findings' tables.<br>**Types of measures:**<br>1. Standardised diagnostic assessment instruments (Childhood Autism Rating Scale, Autism Diagnostic Interview- Revised, Autism Diagnostic Observation Schedule, Diagnostic Interview for Social and Communication Disorders).<br>2. Standardised communication assessments.<br>3. Quality of life questionnaires.<br>4. Rating scales of emotions and behaviour, including depression, anxiety, aggression, obsessive-compulsive behaviour and social reciprocity.<br>5. Global Clinical Impression Rating Scales.<br>6. Other Health Outcome Rating Scale.
**Inclusion Criteria**	Randomised controlled trials (RCTs).
**Exclusion Criteria**	-

Quality of Evidence

Quality of this Systematic Review or Health Technology Assessment

CCE staff appraised the methods used in this publication and found the **risk of bias** to be **LOW.** This means that you can use the findings of the review with confidence as all of the quality criteria have been fulfilled or where criteria have not been fulfilled it is very unlikely the conclusions of the study would be affected.

Quality of the *evidence contained* in this Systematic Review or Health Technology Assessment

The review authors appraised the available evidence and found it to consist of **Level II Evidence (one or more randomised controlled trials)**. The available evidence included in the review is of **variable quality.**

Consistency with Southern Health documented practice

No Southern Health policies or procedures on this topic were identified.

Response

- Click once on the shaded box to select the appropriate response
- Click once on the shaded rectangle to provide a typed comment

Practice at Southern Health (please select one response only, tick the box and provide relevant details)

☐ Not applicable at Southern Health *eg the patient group is not treated at Southern Health* (please explain)

☐ Practice is consistent with the evidence (please add comments if relevant)

☐ Practice is not consistent with the evidence for a good reason (please explain)

☐ Practice was not consistent with the evidence, remedial action has been undertaken and completed (please explain)

☐ Practice is not consistent with the evidence and remedial action has been commenced/planned (please explain)

Communication

Should this information be disseminated more widely? If so, to whom?

Other comments

Feedback

This is a pilot of new processes being implemented by the Technology Clinical Practice Committee and the Centre for Clinical Effectiveness Evidence Dissemination Service.

We would appreciate any comments regarding what works, what doesn’t work and how we can improve the process.

**Name:**			
**Position:**		**Date:**	

Thank you

Southern Health

Technology/Clinical Practice Committee
Evidence Dissemination Service Report for EMT
July 2012

Bulletins SENT from December 2011 to June 2012	Number of Bulletins	Number of Program recipients	RESPONSE REQUIRED												FOR INFORMATION ONLY				
							Medicine												
			**Allied Health**	**Med Services & Quality**	**Nursing & Midwifery**	**Other**	Critical Care	Emergency & Amb Care	Gen Med, AAU, HITH	Medicine	Mental Health	Specialty	Surgery	Women's & Children's	**Diagnostic Imaging**	**Pathology**	**Pharmacy**	**Procurement**	**Other**
Evidence of potential HARM	17	18					2			8	1	2	1	4			6		2
Evidence of CLINICAL EFFECTIVENESS	31	33						1		8	2	8	4	10			18		8
Evidence of COST EFFECTIVENESS^	1	1				1													
Evidence of LACK OF EFFECT^	3	3										1		2		1			
**Total publications requiring a response**	**52**	**55**				**1**	**2**	**1**		**16**	**3**	**11**	**5**	**16**		**1**	**24**		**10**
Evidence of CLINICAL EFFECTIVENESS – for information only*	15	19																	19
Evidence of OTHER EFFECTIVENESS – for information only*	1																		1
Lack of evidence – for information only	107	163													4	1	32		126
**Total publications**	**175**	**237**				**1**	**2**	**1**		**16**	**3**	**11**	**5**	**16**	**4**	**2**	**56**		**156**

*For the April 2012 EDS bulletins the TCPC decided only to request responses for evidence of **harm**, **cost effectiveness** and **evidence of lack of effect**. **Clinical effectiveness, other effectiveness** and **lack of evidence** were provided for information only.

^Responses for these bulletins are due by the end of August 2012.

All responses RECEIVED (December 2011 to June 2012)

	**Total number of responses**	**Allied Health**	**Med Services & Quality**	**Nursing & Midwifery**	**Other**	**Medical Programs**								**Comments**
						Critical Care	Emergency & Amb Care	Gen Med, AAU, HITH	Medicine	Mental Health	Specialty	Surgery	Women's and Children's	
Responses due **by the end of June 2012** from 48 Bulletins	52					2	1		16	4	10	5	14	
Responses received	43					2	1		14	2	10	0	14	
Responses overdue	9					0	0		2	2	0	5	0	
▪ **Consistent with the evidence**	**32**					1	1		11	2	8		9	
▪ **Not applicable at Southern Health**	**7**													
➢ Neuromodulators for pain management in rheumatoid arthritis **(Potential Harm).**									1					The options mentioned in the conclusion are not available on our PBS, so useless for our patients
➢ Botulinum toxin for the treatment of strabismus **(Potential Harm).**											1			Botulinum toxin injection is not practised at Southern Health Ophthalmology Department
➢ Eslicarbazepine acetate add-on for drug-resistant partial epilepsy **(Clinical Effectiveness).**											1			The drug is not in use in Australia and it does not appear in the TGA database. It is not helpful to examine data relating to drugs/devices not available in this country.
➢ Gonadotropin-releasing hormone agonist versus HCG for oocyte triggering in antagonist assisted reproductive technology cycles **(Potential Harm).**													1	IVF not undertaken at Southern Health.
➢ Interventions for pregnant women with hyperglycaemia not meeting gestational diabetes and type 2 diabetes diagnostic criteria **(Clinical Effectiveness).**<br>Respondent reported this as 'Not applicable', however CCE would categorise this response as 'Not consistent with the evidence, remedial action commenced'.													1	The diagnosis and management of GDM and hyperglycaemia not meeting GDM guidelines is currently under national and local review. The Pregnancy Diabetes service at Southern Health has already initiated changes to current practice to conform to (new) ADIPS recommendations. The service is also completing on-going research to guide future practice.
➢ Cabergoline for preventing ovarian hyperstimulation syndrome **(Clinical Effectiveness).**													1	Southern Health does not do IVF.
➢ Milnacipran for neuropathic pain and fibromyalgia in adults **(Potential Harm).**						1								Not applicable to Pain Medicine Unit - agent not used at all

	**Total number of responses**	**Allied Health**	**Med Services & Quality**	**Nursing & Midwifery**	**Other**	**Medical Programs**								**Comments**
						Critical Care	Emergency & Amb Care	Gen Med, AAU, HITH	Medicine	Mental Health	Specialty	Surgery	Women's and Children's	
▪ **Not consistent with the evidence for a good reason**	**3**													
➢ Naftidrofuryl for dementia **(Clinical Effectiveness).**									1					Drug not available in Australia
➢ Cognitive stimulation to improve cognitive functioning in people with dementia **(Clinical Effectiveness).**									1					To my knowledge, specific interventions for patients with dementia while ideal and what we aspire to is very limited in the subacute inpatient setting (e.g. GEM) due to lack of resources and time.
➢ Short and long term effects of tibolone in postmenopausal women **(Potential Harm).**													1	Most menopausal women use combined HRT. Select groups need tibolone due to low libido or abnormal bleeding on HRT.
▪ **Not consistent with the evidence, remedial action has been undertaken and completed**														
▪ **Not consistent with the evidence and remedial action has been commenced/planned**	**1**													
➢ Perineal techniques during the second stage of labour for reducing perineal trauma. **(Clinical Effectiveness).**													1	This Cochrane Review will be looked at by the Maternity Guideline Development Group and existing practices reviewed

Section 22 Model 2 Pilot implementation and evaluation

Pilot objective

To test and refine the features of Model 2.

Characteristics of the pilot intervention

The scope, components and methods described formed the pilot intervention. Pilot activities were undertaken with a pragmatic sample of publications containing evidence of harm. A catalogue of disinvestment opportunities had been compiled to identify pilot disinvestment projects for investigation in the SHARE Program [79]. Publications with high quality evidence indicating harm published in the previous two years were selected.

Pilot implementation

The implementation strategies focused on integrating the new processes into existing Monash Health infrastructure and communicating with stakeholders.

The procedure for the new EDS processes was documented and a routine item for discussion of EDS matters was included in the TCPC agenda.

The Director of CCE/SHARE Director made presentations to the Executive Management Team, Medical and Nursing Executive groups, and met with clinical directors of all medical programs, allied health, pharmacy, pathology, diagnostic imaging and procurement. The Chair of the TCPC delivered a presentation to the Monash Health Board. All senior managers expressed their support for the proposed governance structure. A letter outlining the new process was sent to stakeholders by the Executive Director of Medical Services and Quality and a flyer was circulated to the 'All Staff' email list by the Chair of the TCPC.

Pilot evaluation

The stakeholders listed above were asked to provide feedback regarding the new processes, and templates for feedback were included at the end of the Evidence Bulletins.

An audit of responses was undertaken two months after dissemination of the pilot bulletins.

Reach

Six evidence bulletins indicating harm were forwarded by Program Directors to the relevant decision-makers (Medicine Program 3, Women's and Children's Program 1, Specialty Program 1, Critical Care Program 1).

Four out of six responses from decision-makers were received by the due date (one month after receipt). The others were received after reminders were sent. The average time to respond was 28 days.

Bulletins were received and returned by the appropriate decision-makers.

Usefulness

No feedback was received regarding 'what worked, what didn't work and how we can improve the new process'; one person said "*Thanks*" on the feedback sheet.

Use

Five responses indicated that practice was consistent with the evidence, the sixth reported that the practice was not undertaken at Monash Health. No action was required in these cases.

One respondent indicated that the evidence should be communicated to other programs and it was forwarded accordingly.

Implementation fidelity

There were no modifications to the planned intervention and it was implemented as planned.

Southern Health

Technology/Clinical Practice Committee

Ensuring Southern Health practice is up-to-date

The Technology/Clinical Practice Committee (TCPC) is introducing a new process to ensure that practice at Southern Health is consistent with current evidence.

Capture evidence summaries as they are published

↓

Screen for quality and applicability to Southern Health

↓

Compare current practice with up-to-date evidence

↙ ↘

Confirm that current practice is consistent with evidence | **Take action to ensure practice is consistent with evidence**

The Centre for Clinical Effectiveness (CCE) had developed an Evidence Dissemination Service to capture high quality evidence as it is published. The TCPC will disseminate this to the relevant decision-makers who will be asked to consult with colleagues and report back on any action required to align current Southern Health practice with the most up-to-date evidence.

The process has been developed to minimise your time and effort.

- Only synthesised information such as systematic reviews, health technology assessments and evidence based guidelines will be provided. You will not receive trials or other primary studies, editorials or opinion pieces.
- The synthesised evidence is retrieved from high quality sources and will be appraised by CCE staff so that you can be confident the information is trustworthy.
- CCE staff will compare the evidence with current policies and procedures. If Southern Health documentation is consistent with the evidence, you will be informed but no response is required.
- A response will only be required if there are no policies and procedures on this topic or if the current policies and procedures are inconsistent with the latest evidence.
- Action will only be required if current practice is inconsistent with up-to-date high quality evidence that is relevant and applicable to Southern Health.
- Responses will be required within an appropriate time frame. These have been determined to prioritise action to areas of greatest risk to patients, staff or the organisation. Where there is
  - **evidence of harm**, a response will be required in **1 month**
  - **evidence of benefit**, a response will be required in **3 months**
  - **evidence of a more cost-effective alternative**, a response will be required in **3 months**
  - **evidence of lack of effect**, a response will be required in **6 months**
  - **lack of evidence**, the publication will be provided for information only, **no response required**

The new process will be implemented as a pilot. Your input and suggestions to improve the methods and materials is welcome and encouraged. Please direct your feedback and any questions to:

A/Prof Claire Harris, Centre for Clinical Effectiveness (9594 7576 or claire.harris@monash.edu)

Yours sincerely
A/Prof Richard King
Chair, Southern Health Technology/Clinical Practice Committee

Section 24 Model 2 Evaluation of full implementation

The EDS was discontinued prior to implementation of the planned evaluation activities, however data were collected for the first seven-month period and audited to meet reporting requirements.

Reach

During this period, 175 publications were collected and all categories were represented. The majority (n=107, 61%) found a lack of evidence or insufficient evidence to draw conclusions, followed by clinical effectiveness (n=46, 26%), harm (n=17, 1%), lack of effect (n=3), cost-effectiveness and methodological effectiveness (n=1 each).

Fifty-two bulletins required a response, however three contained information pertaining to two executive or program portfolios, making the total number of responses required 55. The remaining 123 publications were disseminated to 182 recipients for information only.

Of the 55 requiring responses, the Medicine Program and Women's and Children's Program received the most (n=16, 29% each), followed by Specialty (n=11, 20%), Surgery (n=5, 9%), Mental Health (n=3), Critical Care (n=2) and Emergency and Ambulatory Care and Other (n=1 each). A collation of 56 relevant bulletins was provided to Pharmacy, four to Diagnostic Imaging, two to Pathology and 156 to other programs and departments for their information.

Fifty-one of the 55 responses were due at the time of data collection, 4 were due in the following month. Forty-three had been received, 9 were overdue and 3 were pending.

Dissemination to the correct recipients was not formally assessed, however responses indicated that bulletins were received by the appropriate decision-makers.

Six of the 43 respondents recommended that the bulletin be forwarded to others including five internal departments, the Divisions of General Practice, health professionals across the organisation, and one did not specify the distribution.

Usefulness

Respondents reported that local practice was consistent with the evidence (n=32, 74%), the evidence was not applicable at Monash Health (n=6), local practice was not consistent with the evidence for a good reason (n=3), and changes to make practice consistent with the evidence had been commenced or was planned (n=2).

Evidence was not applicable to the Monash Health setting because the practices were not undertaken (n=4) or the specified drugs were unavailable in Australia (n=2). The three reasons for local practice being inconsistent with the evidence for a good reason also included a drug which was unavailable in Australia, plus a lack of resources and time to implement the proposed interventions, and undertaking the practice but restricting it to a specific patient group who were unable to receive the alternative treatment.

Many respondents included comments and feedback in the free text sections of the bulletins. Five offered positive comments, welcoming future bulletins. Although respondents were not specifically asked to comment on usefulness, many suggested it was not "*useful*", "*helpful*" or "*valuable*" to consider evidence that they were already aware of, that was consistent with current practice, or that addressed drugs that were not locally available.

Use

The 43 respondents had clearly read and understood the bulletins, and had used the bulletins to assess whether current practice was consistent with the evidence.

Given that the aim of the EDS was to use evidence proactively to drive decisions, 'use' in this context could be interpreted as leading to practice change. Two decision-makers noted that local practice was not consistent with the evidence. One department had already *"initiated changes to current practice to conform to the recommendations",* and the other had tasked their guideline development group to address the inconsistency.

Bulletins could also be 'used' to confirm that current practice does not need to be changed, but the 'usefulness', cost-effectiveness and impact of resource use in achieving this was questioned in respondent's feedback and project team and committee reflections.

Resources

Delivery of the EDS was undertaken by the EDS Administration Officer (approximately two days per week to capture and process publications and develop bulletins, three days per month to prepare reports and documents for TCPC meetings and attend the meetings), the CCE Director (approximately one half day per week to review processes and bulletins, one day per month to prepare for and attend the TCPC meetings), the TCPC Chair (approximately half day per month to consult with EDS staff and review publications for local applicability), and the TCPC members (approximately 30 minutes per month discussing EDS issues).

Implementation fidelity

There were two major modifications to the planned intervention, both were due to resource limitations. Three months after implementation, the scope was revised to focus only on evidence in areas of high priority to the organisation. Including evidence of harm was essential for patient safety, and adding evidence of cost-effectiveness and lack of effect would complement current Monash Health initiatives ascertaining examples of more cost-effective alternatives and identifying organisational waste in clinical and corporate practices. Only publications with evidence in these three areas would be appraised prior to dissemination and would require a response. Evidence of clinical effectiveness, methodological effectiveness and lack of evidence were provided for information only. Three months later, the EDS was suspended altogether due to limited capacity within CCE.

There were no changes to the implementation plan and barriers and enablers are discussed with factors influencing processes and outcomes below.

Project team and committee reflections

<table>
<tr><td>Pros
• Systematic
• Transparent, Accountable, Evidence-based
• Not just for 'disinvestment', applies to all practices
• Focuses on important changes
• Does not burden clinicians and managers with process
• Does not require clinicians and managers to have skills
• Does not require health economist</td><td>Cons
• Sustainability – resources a problem
• How much activity can the organisation sustain?
• 'Top down'
    • Buy in/ownership
    • When should stakeholders be involved</td></tr>
<tr><td colspan="2">▪ First couple of rounds just sending things out, minimal effort in responses
▪ Fourth round – aware of additional things
    – More than one conclusion – sometimes harm plus effect, sometimes effect plus lack of evidence, etc. Need to develop new way of capturing this and need more than one response – how to collect this?
    – More complex issues arising eg three reviews on wound management. Review of policies and procedures shows us we don't have enough information to know whether evidence is consistent. Could initiate a project rather than just asking for a response eg we look at other reviews on wound care, look at our local data re relevant patient groups/care/types of dressings/costs/etc. In these cases more than one person is responsible for decision – perhaps need a project rather than response to a single Evidence Bulletin to sort these out
    – While our policies and procedures might not be absent or inconsistent with current evidence, they may not have enough information. Eg blood transfusion in oncology patients at end of life. We have appropriate guidance re blood transfusions generically and in oncology patients, but nothing specifically about end of life. How do we use the EDS process to address this?
    – These complex considerations require high level methodological and clinical knowledge beyond the skills of EDS project officer. Need more senior evidence staff and clinical involvement.
    – The authors' conclusions are not good enough. Conclusions in systematic reviews often not straightforward, often can't work out what the outcomes were or what type of evidence eg harm etc. Only sent bulletins when we were confident that we understood the authors' conclusions or recommendations.
    – We have only included level and quality of evidence in our summaries, but now it is clear that use of the information by clinicians requires information regarding statistical and clinical significance, therefore need to add this. Should also qualify our evidence classification eg evidence of effectiveness but of uncertain clinical significance
    – This is academic hospital, respondents correctly point out that they are involved in writing national guidelines and don't want EDS to waste their time reviewing said guidelines. But how does EDS know who knows/doesn't know?</td></tr>
</table>

Decision-maker's responses

**Positive comments**<br>▪ Thanks<br>▪ Good idea (n=2)<br>▪ This department would welcome receiving any future results of related CCE literature searches.<br>▪ This Cochrane Review will be looked at by the Guideline Development Group and existing practices reviewed
**Drug not available in Australia**<br>▪ The options mentioned in the conclusion are not available on our PBS, so useless for our patients.<br>▪ Why are you reviewing a drug that is not available in Australia?<br>▪ The drug is not in use in Australia and it does not appear in the TGA database. It is not helpful to examine data relating to drugs/devices not available in this country
**Confusion over aim and/or impression that CCE undertook the review being disseminated**<br>▪ This department would welcome receiving any future results of related CCE literature searches.<br>▪ It is unclear what this process is trying to achieve. At present it is increasing my workload - but has not changed the practices of the unit.<br>▪ It would be worthwhile discussing the scope of proposed clinical effectiveness projects prior to undertaking the review so that this work can be better tailored to meet the needs of clinicians and others working in the field.<br>▪ Would be interested to see the rate of dependence related to this practice, as this is the issue seen by the time patients on this drug get to our unit.<br>▪ The conclusions are well known amongst specialists in this area. The exercise has not been helpful. There is no value in broad dissemination of results / guidelines etc.<br>▪ This type of review process needs to target populations more relevant to the hospital setting.<br>▪ What is the process to determine topics and priorities?
**Academic health service, respondents familiar with evidence and/or involved in reviews and guideline development**<br>▪ This has been the practice here for over 15 years. I'm not sure this process is an effective use of people's time. Most disciplines would be well aware of developments within their own discipline, even if the rest of the hospital is not. Especially in academically focussed units like my own, members of the team are involved in writing systematic reviews and national guidelines on topics like this.<br>▪ I was involved in Australian section of this literature review project - small part of a bigger review.<br>▪ Clinicians will usually have already seen the papers upon which the recommendations are based, or may even be undertaking primary research in the field themselves - and may be able to make valuable contributions to the planning of these projects.<br>▪ The diagnosis and management of this condition not meeting guidelines is currently under national and local review. Our department has already initiated changes to current practice to conform to (new) recommendations. The service is also completing on-going research to guide future practice.<br>▪ The conclusions are well known amongst specialists in this area. The exercise has not been helpful.<br>▪ Prophylactic antibiotic should not be used. That is why it is not in our protocol.
**Evidence not applicable or not of high quality**<br>▪ The 'evidence' was obtained from two very small trials that showed some treated patients had relatively minor adverse events. More importantly, in regard to potential adverse events that are subject to investigator interpretation, the authors state "Lack of blinding in one trial out of the two in total that reported on adverse events may result in biased results". Furthermore, in regard to biochemical results, that in theory should be less subject to bias, they state "Accordingly, the result of our meta-analysis for this outcome is not a robust result". I conclude that this review does not help to decide whether this treatment is useful or harmful in these patients. Don't send low quality reviews for comment<br>▪ Good idea – but this issue has little clinical relevance.<br>▪ The Cochrane review should not be used as a source of information. The report on this review is not quite correct. For a meta-analysis based on a small number of subjects and trials and with some trials being open labelled, the findings can be unreliable.<br>▪ This is a primary care issue and few children presenting here have this as a sole problem. For the few patients - especially young we use guidelines that recommend not using these therapies. This type of review process needs to target populations more relevant to the hospital setting.
**Need for additional information in bulletin**<br>▪ This report to the clinicians should provide details such as the number of subjects with placebo control or open label trials. Further the person writing the report should look at the setting of the trials, provide details on the type of antibiotic, the change in the frequency of antibiotic resistance and the cost to the hospitals.

This protocol was a work in progress at the time the EDS was suspended.

Southern Health

Technology/Clinical Practice Committee Evidence Bulletin_100

This bulletin is part of a process to ensure that Southern Health practice is consistent with current evidence. Your response is required by the date below. You can find more information about this process on the TCPC website.

The publication below indicates evidence of **CLINICAL EFFECTIVENESS** related to **Blunt versus sharp suture needles for preventing percutaneous exposure incidents in surgical staff.**

Responses related to evidence of **CLINICAL EFFECTIVENESS** are required within **THREE months.**

Please complete and return this bulletin to marie.garrubba@monash.edu by **1 June 2012**

Bibliographic Source

Parantainen A, Verbeek JH, Lavoie MC, Pahwa M. Blunt versus sharp suture needles for preventing percutaneous exposure incidents in surgical staff. *Cochrane Database of Systematic Reviews* 2011, Issue 11. Art. No.: CD009170. DOI: 10.1002/14651858.CD009170.pub2. http://onlinelibrary.wiley.com/doi/10.1002/14651858.CD009170.pub2/pdf

Author's Conclusion

There is high quality evidence that the use of blunt needles appreciably reduces the risk of exposure to blood and bodily fluids for surgeons and their assistants over a range of operations. It is unlikely that future research will change this conclusion.

Applicability to Southern Health

**Patient / Population**	Persons working in the operation theatre that are exposed to the risk of percutaneous injuries with suture needles.
**N**	2961 participating surgeons
**Setting**	UK, US, Germany, Italy, Ireland, Netherlands. Four studies focused on abdominal closure, two on vaginal repair and two on hip replacement.
**Intervention**	Blunted suture needles (we defined blunt needles as suture needles that have a rounded blunt point and that are circular in diameter and that can be either curved or straight)
**Comparison**	Sharp needles (sharp needles are suture needles that have a tapered point and that can be either circular in diameter or square with cutting edges and that can be either curved or straight).
**Outcomes**	**Primary**<br>Exposure of healthcare workers to contaminated blood or bodily fluids was our primary outcome measure. Exposure can be observed either as self-reported needle stick injury or glove perforations.<br>**Secondary**<br>We included satisfaction with, or ease of use of, the needles.
**Inclusion Criteria**	Randomised clinical trials (RCTs) and cluster-randomised trials (c-RCTs).<br>*"Persons working in the operation theatre"*<br>"Blunt suture needles (rounded blunt point that are circular in diameter and that can be either curved or straight) compared to sharp suture needles (tapered point, can be circular in diameter or square with cutting edges and can be curved or straight)"
**Exclusion Criteria**	Intervention was a needle handling device and not a blunt needle, study not randomised or controlled.

Quality of Evidence

Quality of this Systematic Review or Health Technology Assessment

CCE staff appraised the methods used in this publication and found the **risk of bias** to be **LOW.** This means that you can use the findings of the review with confidence as all of the quality criteria have been fulfilled or where criteria have not been fulfilled it is very unlikely the conclusions of the study would be affected.

Quality of the *evidence contained* in this Systematic Review or Health Technology Assessment

The review authors appraised the available evidence and found it to consist of **Level II Evidence (one or more randomised controlled trials)**. The available evidence included in the review is of **high quality.**

Consistency with Southern Health documented practice

No Southern Health policies or procedures on this topic were identified.

Additional analysis prior to dissemination

Priority setting

1. **Is this a priority for Southern Health?**

Scope

2. **Who needs to be consulted (patients, clinicians, others) and where are they?**

Directors (medical and nursing) of Operating Suite, Surgery, Specialty, Women's & Children's, and Procurement, and Chair of Operating Suite Product Evaluation Committee.

3. **Are there other parameters that need to be considered (setting, condition, professional groups, others)?**

Problem

4. **What is the problem?**

The systematic review recommends the use of blunt needles to reduce the rate of exposure to blood and bodily fluids for surgeons and their assistants over a range of operations. Southern Health use both blunt and sharp suture needles.

5. **Is it a real problem or perceived problem?**

6. **Is there a gap (not being done at all) or mismatch (need to change current practice)?**

Size/extent

7. **How can it be measured (routinely collected data or collect our own data)?**

8. **How big is the problem at Southern Health?**

Ethical considerations

9. **Do any ethical issues arise regarding the dissemination of this bulletin?**

Solutions

10. **What does the literature identified in EDS say?**

11. **What are the options available? Pros? Cons?**

Additional Questions

What is the rate of stick injuries at Southern Health?

Are they comparable to the Systematic Review?

Is there a cost difference between using blunt compared with sharp suture needles?

Do some procedures require a sharp versus blunt needle?

What is the proportion of Southern Health surgeons using blunt versus sharp needles?

Study: Parantainen, A., Verbeek, J.H., Lavoie, M.C., Pahwa, M. (2011). Blunt versus sharp suture needles for preventing percutaneous exposure incidents in surgical staff. Cochrane Database of Systematic Reviews. 11, Art. No.: CD009170.

Description of study: Systematic review of RCTs

**Patient/population**	Persons working in the operation theatre that are exposed to the risk of percutaneous injuries.		
**N**	10 studies, n= Surgeons 2961 (total gloves unclear)		
**Setting**	UK, US, Netherlands, Italy and Germany. Four studies focused on abdominal closure, two on vaginal repair and two on hip replacement		
**Intervention/indication & Comparison/control**	**Reference**	**Intervention**	**Comparison**
	Ablett 1998 (195 pairs of gloves)	Suturing with blunt tipped needles<br>104 surgeons-operations	Suturing with sharp needles<br>91 surgeons-operations
	Hartley 1996 (85 pairs of gloves)	Suture needles with blunted end<br>46 surgeon-operations	Conventional sharp pointed needles<br>39 surgeon-operations
	Meyer 1996 (400 gloves)	Blunt needles<br>98 surgeon-operations	Sharp needles<br>102 surgeon-operations
	Mingoli 1996 (1560 gloves)	Blunt Needles<br>300 surgeon-operations	Sharp needles<br>300 surgeon-operations
	Nordkam 2005 (406 pairs of gloves)	Blunt-tapered needles<br>200 surgeon-operations	Sharp needles<br>100 surgeon-operations
	Rice 1996	Taper pointed suture needles<br>34 surgeon-operations (# of gloves not reported)	Standard pointed needles<br>34 surgeon-operations (128 gloves)
	Sullivan 2009	Blunt needles<br>204 surgeon-operations	Sharp needles<br>204 surgeon-operations
	Thomas 1995	Blunt tipped needles<br>Assumed 40 surgeon-operations	Sharp tipped needles<br>Assumed 40 surgeon-operations
	Wilson 2008	Blunt Needles<br>217 surgeon-operations (All gloves collected)	Sharp needles<br>221 surgeon-operations (All gloves collected)
	Wright 1993	Blunt taper point suture needles<br>38 surgeon-operations	Standard cutting needles<br>31 surgeon-operations
**Outcomes**	**Primary:**<br>Exposure of healthcare workers to contaminated blood or bodily fluids. Exposure could be self reported needle stick injury or glove perforations.<br>**Secondary:**<br>Satisfaction with or ease of use of the needles		
**Inclusion Criteria**	*"RCTs and Cluster-RCTs"*<br>"Persons working in the operation theatre"<br>"Blunt suture needles (rounded blunt point that are circular in diameter and that can be either curved or straight) compared to sharp suture needles (tapered point, can be circular in diameter or square with cutting edges and can be curved or straight)"		
**Exclusion Criteria**	Intervention was a needle handling device and not a blunt needle, study not randomised or controlled.		

SR/HTA Objective		
To determine the effectiveness of blunt needles compared to sharp needles for preventing percutaneous incidents among surgical staff.		
**Study Validity**		
**Is it clear that there were no conflicts of interest in the writing or funding of this review?**	Yes	The authors report that there were no conflicts of interest associated with this review.
**Does the review have a clearly- focused question?**	Yes	
**Is a systematic review the appropriate method to answer the question?**	Yes	
**Does the review have specified inclusion/exclusion criteria?**	Yes	See above
**If there were specified inclusion/ exclusion criteria, were these appropriate?**	Yes	
**Does the review document a comprehensive search strategy?**	Yes	See appendix 1.
**Were reviewers blind to authors, institutions and affiliations?**	Not reported	
**Were 2 or more independent reviewers used for:**<br>**1. application of inclusion criteria to assess eligibility of studies?**	Yes	"two authors working independently screened the identified titles and abstracts of the references that resulted from the search strategy for potential studies."
**2. extraction of data from study reports?**	Yes	As above
**3. appraisal of study quality?**	Yes	See potential biases in the review process pg15
**Were the strengths and limitations of included studies and potential impact on the results discussed?**	Yes	See page 10 'Risk of bias in included studies'
**Was the validity of included trials appraised using appropriate criteria?**	Yes	It is unclear if more than one assessor appraised the validity of the included trials.<br>Studies were appraised based on random sequence generation, allocation concealment, blinding of participants and personnel, blinding of outcome assessment, incomplete outcome data, selective reporting and other bias.
**Is there a summary of the results of individual studies?**	Partial	Summary of main results provided but not for individual studies.
**If meta-analyses were conducted, was it reasonable to do so?**	Yes	
**If meta-analyses were conducted, was it done appropriately?**	Yes	
**Other**		
**What is the overall risk of bias?**	Low	Low - All of the criteria have been fulfilled or where criteria have not been fulfilled it is very unlikely the conclusions of the study would be affected.
**Results**		
**Cochrane Summary of Findings Table**		

Blunt needles compared to sharp needles for preventing percutaneous exposure injuries

Patient or population: surgical staff
Intervention: blunt needles
Comparison: sharp needles

Outcomes	Illustrative comparative risks* (95% CI)		Relative effect (95% CI)	No of Participants (studies)	Quality of the evidence (GRADE)	Comments
	**Assumed risk**	**Corresponding risk**				
	**sharp needles**	**blunt needles**				
**glove perforations per surgeon per operation**	**Study population**		**RR 0.46** (0.38 to 0.54)	2961 (10 studies)	⊕⊕⊕⊕ **high**	
	**293 per 1000**	**135 per 1000** (111 to 158)				
	**Low risk population**					
	**20 per 1000**	**9 per 1000** (8 to 11)				
	**High risk population**					
	**750 per 1000**	**345 per 1000** (285 to 405)				

*The basis for the **assumed risk** (e.g. the median control group risk across studies) is provided in footnotes. The **corresponding risk** (and its 95% confidence interval) is based on the assumed risk in the comparison group and the **relative effect** of the intervention (and its 95% CI).
CI: confidence interval; **RR:** risk ratio;

GRADE Working Group grades of evidence
High quality: Further research is very unlikely to change our confidence in the estimate of effect.
Moderate quality: Further research is likely to have an important impact on our confidence in the estimate of effect and may change the estimate.
Low quality: Further research is very likely to have an important impact on our confidence in the estimate of effect and is likely to change the estimate.
Very low quality: We are very uncertain about the estimate.

Figure 4. Forest plot of comparison: 1 Blunt versus sharp suture needles, outcome: 1.1 Glove perforation rate.

Study or Subgroup	log[Risk Ratio]	SE	Weight	Risk Ratio IV, Fixed, 95% CI	Year
Wright 1993	-0.7472144	0.296334	8.6%	0.47 [0.27, 0.85]	1993
Thomas 1995	-0.35667494	0.348466	6.2%	0.70 [0.35, 1.39]	1995
Meyer 1996	-0.85131877	0.138984	39.2%	0.43 [0.33, 0.56]	1996
Rice 1996	-3.11351531	1.449138	0.4%	0.04 [0.00, 0.76]	1996
Mingoli 1996	-0.73315252	0.173816	25.1%	0.48 [0.34, 0.68]	1996
Hartley 1996	-1.70552479	0.636209	1.9%	0.18 [0.05, 0.63]	1996
Ablett 1998	-0.64435702	0.421637	4.3%	0.52 [0.23, 1.20]	1998
Nordkam 2005	-0.8303483	0.290628	9.0%	0.44 [0.25, 0.77]	2005
Wilson 2008	-0.2048782	0.67082	1.7%	0.81 [0.22, 3.03]	2008
Sullivan 2009	-0.8873032	0.449089	3.8%	0.41 [0.17, 0.99]	2009
**Total (95% CI)**			**100.0%**	**0.46 [0.38, 0.54]**	

Heterogeneity: $Chi^2 = 7.45$, df = 9 (P = 0.59); $I^2 = 0\%$
Test for overall effect: Z = 9.03 (P < 0.00001)

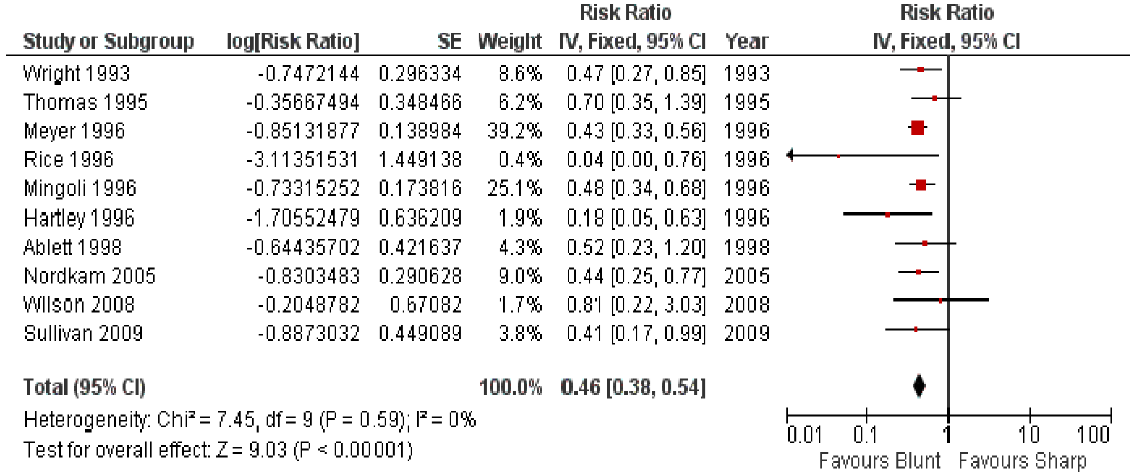

Outcome: number of glove perforations

"Ten trials including 2961 surgeon-operations compared the effect of blunt versus sharp needles on glove perforations and found a significant reduction of glove perforations, with a relative risk of glove perforations of 0.46 (95% confidence interval 0.38 to 0.54)."

Outcome: percutaneous injuries

"Five studies reported the number of percutaneous injuries but in one study there were no injuries in the intervention and control groups. We could combine four studies in a meta-analysis. The use of blunt needles decreased the risk of a needle stick injury by 69% (RR 0.31, 95% CI 0.14 to 0.68)."

Outcome: surgeon satisfaction and needle performance

"Data on needle performance could only be extracted from Nordkam (2005) and Meyer (1996). Nordkam (2005) showed that surgeons found the sharp needles 20% easier to use, on a VAS scale from 0 to 100, but Meyer (1996) reported that the blunt needles were easier to use even though, clearly more force was needed."

"Rice (1996) reported that surgeons had no difficulties with the use of the blunt needles. In Sullivan (2009), 92% of the surgeons reported that the blunt needles were acceptable but they were less satisfied with their use. Wilson (2008) reported that surgeons found the blunt needles significantly more difficult to use. In Wright (1993) the surgeons found the blunt needles slightly more difficult to use but they had minimal effect on their technique."

Author's Conclusions

"**Implications for practice**: There is high quality evidence that the use of blunt suture needles appreciably reduces the risk of exposure to blood and bodily fluids for surgeons and their assistants over a range of operations."

"**Implications for research**: There is high quality evidence that the use of blunt needles is beneficial and it is unlikely that future research will change this conclusion."

Out Comments/Summary

The overall risk of bias in this systematic review is low.

The authors suggest that the use of blunt suture needles appreciably reduces the risk of exposure to blood and bodily fluids for surgeons and their assistants over a range of operations. This is a justified conclusion based on the statistical significance of the reduction of 54% of the risk of glove perforations and 69% reduction in the risk of needle stick injuries when using blunt needles.

The Systematic review was well carried out with no conflicts of interest reported.

Response

- Click once on the shaded box to select the appropriate response
- Click once on the shaded rectangle to provide a typed comment

Practice at Southern Health (please select one response only, tick the box and provide relevant details)

☐ Not applicable at Southern Health *eg the patient group is not treated at Southern Health* (please explain)

☐ Practice is consistent with the evidence (please add comments if relevant)

☐ Practice is not consistent with the evidence for a good reason (please explain)

☐ Practice was not consistent with the evidence, remedial action has been undertaken and completed (please explain)

☐ Practice is not consistent with the evidence and remedial action has been commenced/planned (please explain)

Communication

Should this information be disseminated more widely? If so, to whom?

Other comments

Feedback

This is a pilot of new processes being implemented by the Technology Clinical Practice Committee and the Centre for Clinical Effectiveness Evidence Dissemination Service.

We would appreciate any comments regarding what works, what doesn't work and how we can improve the process.

**Name:**			
**Position:**		**Date:**	

Thank you

Section 26 References

1. SurveyMonkey, . SurveyMonkey Inc., Palo Alto, California, USA. www.surveymonkey.com. Accessed March 2016.
2. Taylor R, Reeves B, Mears R, Keast J, Binns S, Ewings P et al. Development and validation of a questionnaire to evaluate the effectiveness of evidence-based practice teaching. Medical education. 2001;35(6):544-7.
3. NVivo qualitative data analysis software Version 8. QSR International Pty Ltd. 2008. http://www.qsrinternational.com/. Accessed 24/10/2017.
4. Michie S, Johnston M, Abraham C, Lawton R, Parker D, Walker A. Making psychological theory useful for implementing evidence based practice: a consensus approach. Quality & safety in health care. 2005;14(1):26-33. doi:10.1136/qshc.2004.011155.
5. Harris C, Allen K, Waller C, Brooke V. Sustainability in Health care by Allocating Resources Effectively (SHARE) 3: Examining how resource allocation decisions are made, implemented and evaluated in a local healthcare setting BMC health services research. 2017. doi:10.1186/s12913-017-2207-2.
6. Harris C, Allen K, Waller C, Green S, King R, Ramsey W et al. Sustainability in Health care by Allocating Resources Effectively (SHARE) 5: Developing a model for evidence-driven resource allocation in the local healthcare setting BMC health services research. 2017. doi:10.1186/s12913-017-2208-1.
7. Harris C, Allen K, Waller C, Dyer T, Brooke V, Garrubba M et al. Sustainability in Health care by Allocating Resources Effectively (SHARE) 7: Supporting staff in evidence-based decision-making, implementation and evaluation in a local healthcare setting BMC health services research. 2017. doi:10.1186/s12913-017-2388-8.
8. Dobbins M, Cockerill R, Barnsley J. Factors affecting the utilization of systematic reviews. A study of public health decision makers. International journal of technology assessment in health care. 2001;17(2):203-14.
9. Dobbins M, Ciliska D, Cockerill R, Barnsley J, DiCenso A. A framework for the dissemination and utilization of research for health-care policy and practice. Online J Knowl Synth Nurs. 2002;9:7.
10. Dobbins M, DeCorby K, Twiddy T. A knowledge transfer strategy for public health decision makers. Worldviews on evidence-based nursing / Sigma Theta Tau International, Honor Society of Nursing. 2004;1(2):120-8. doi:10.1111/j.1741-6787.2004.t01-1-04009.x.
11. Dobbins M, Jack S, Thomas H, Kothari A. Public health decision-makers' informational needs and preferences for receiving research evidence. Worldviews on evidence-based nursing / Sigma Theta Tau International, Honor Society of Nursing. 2007;4(3):156-63. doi:10.1111/j.1741-6787.2007.00089.x.
12. Armstrong R, Waters E, Crockett B, Keleher H. The nature of evidence resources and knowledge translation for health promotion practitioners. Health promotion international. 2007;22(3):254-60. doi:10.1093/heapro/dam017.
13. Dobbins M, Hanna SE, Ciliska D, Manske S, Cameron R, Mercer SL et al. A randomized controlled trial evaluating the impact of knowledge translation and exchange strategies. Implementation science : IS. 2009;4:61. doi:10.1186/1748-5908-4-61.
14. Lavis JN, Robertson D, Woodside JM, McLeod CB, Abelson J. How can research organizations more effectively transfer research knowledge to decision makers? The Milbank quarterly. 2003;81(2):221-48, 171-2.
15. Haynes RB, Holland J, Cotoi C, McKinlay RJ, Wilczynski NL, Walters LA et al. McMaster PLUS: a cluster randomized clinical trial of an intervention to accelerate clinical use of evidence-based information from digital libraries. Journal of the American Medical Informatics Association : JAMIA. 2006;13(6):593-600. doi:10.1197/jamia.M2158.
16. Cilenti D, Brownson RC, Umble K, Erwin PC, Summers R. Information-seeking behaviors and other factors contributing to successful implementation of evidence-based practices in local health departments. Journal of public health management and practice : JPHMP. 2012;18(6):571-6. doi:10.1097/PHH.0b013e31825ce8e2.
17. Dobbins M, Rosenbaum P, Plews N, Law M, Fysh A. Information transfer: what do decision makers want and need from researchers? Implementation science : IS. 2007;2:20. doi:10.1186/1748-5908-2-20.
18. Lavis JN. How can we support the use of systematic reviews in policymaking? PLoS medicine. 2009;6(11):e1000141. doi:10.1371/journal.pmed.1000141.
19. Bero LA, Grilli R, Grimshaw JM, Harvey E, Oxman AD, Thomson MA. Closing the gap between research and practice: an overview of systematic reviews of interventions to promote the implementation of research findings. The Cochrane Effective Practice and Organization of Care Review Group. BMJ. 1998;317(7156):465-8.
20. Fronsdal KB, Facey K, Klemp M, Norderhaug IN, Morland B, Rottingen JA. Health technology assessment to optimize health technology utilization: using implementation initiatives and monitoring processes. International journal of technology assessment in health care. 2010;26(3):309-16. doi:10.1017/s0266462310000309.
21. Gifford W, Davies B, Edwards N, Griffin P, Lybanon V. Managerial leadership for nurses' use of research evidence: an integrative review of the literature. Worldviews on evidence-based nursing / Sigma Theta Tau International, Honor Society of Nursing. 2007;4(3):126-45. doi:10.1111/j.1741-6787.2007.00095.x.
22. Healy J, Braithwaite J. Designing safer health care through responsive regulation. The Medical journal of Australia. 2006;184(10 Suppl):S56-9.
23. Tricco AC, Cardoso R, Thomas SM, Motiwala S, Sullivan S, Kealey MR et al. Barriers and facilitators to uptake of systematic reviews by policy makers and health care managers: a scoping review. Implementation science : IS. 2016;11(1):4. doi:10.1186/s13012-016-0370-1.
24. Kosteniuk JG, Morgan DG, D'Arcy CK. Use and perceptions of information among family physicians: sources considered accessible, relevant, and reliable. Journal of the Medical Library Association : JMLA. 2013;101(1):32-7. doi:10.3163/1536-5050.101.1.006.

25. Ellen ME, Leon G, Bouchard G, Ouimet M, Grimshaw JM, Lavis JN. Barriers, facilitators and views about next steps to implementing supports for evidence-informed decision-making in health systems: a qualitative study. Implementation science : IS. 2014;9(1):179. doi:10.1186/s13012-014-0179-8.
26. Oliver K, Innvar S, Lorenc T, Woodman J, Thomas J. A systematic review of barriers to and facilitators of the use of evidence by policymakers. BMC health services research. 2014;14(1):2. doi:10.1186/1472-6963-14-2.
27. Wallace J, Byrne C, Clarke M. Improving the uptake of systematic reviews: a systematic review of intervention effectiveness and relevance. BMJ open. 2014;4(10):e005834. doi:10.1136/bmjopen-2014-005834.
28. Evans BA, Snooks H, Howson H, Davies M. How hard can it be to include research evidence and evaluation in local health policy implementation? Results from a mixed methods study. Implementation science : IS. 2013;8:17. doi:10.1186/1748-5908-8-17.
29. Solomons NM, Spross JA. Evidence-based practice barriers and facilitators from a continuous quality improvement perspective: an integrative review. Journal of nursing management. 2011;19(1):109-20. doi:10.1111/j.1365-2834.2010.01144.x.
30. Robert G, Harlock J, Williams I. Disentangling rhetoric and reality: an international Delphi study of factors and processes that facilitate the successful implementation of decisions to decommission healthcare services. Implementation science : IS. 2014;9(1):123. doi:10.1186/s13012-014-0123-y.
31. HealthPACT. Disinvestment in Australia and New Zealand. Health Policy Advisory Committee on Technology 2013. Available from: http://www.health.qld.gov.au/healthpact/docs/papers/workshop/disinvestment-report.pdf. Accessed: 29/09/2017
32. Hughes E, McKenny K. Decommissioning and Disinvestment Toolkit 2013-2014 Rayleigh, Essex, UK: Castle Point and Rochford Clinical Commissioning Group, 2013.
33. Marks L, Weatherly H, Mason A. Prioritizing investment in public health and health equity: what can commissioners do? Public health. 2013;127(5):410-8. doi:10.1016/j.puhe.2013.01.027.
34. Polisena J, Clifford T, Elshaug AG, Mitton C, Russell E, Skidmore B. Case studies that illustrate disinvestment and resource allocation decision-making processes in health care: A systematic review. International journal of technology assessment in health care. 2013;29(2):174-84. doi:10.1017/s0266462313000068.
35. Henshall C, Schuller T, Mardhani-Bayne L. Using health technology assessment to support optimal use of technologies in current practice: the challenge of "disinvestment". International journal of technology assessment in health care. 2012;28(3):203-10. doi:10.1017/s0266462312000372.
36. Watt AM, Hiller JE, Braunack-Mayer AJ, Moss JR, Buchan H, Wale J et al. The ASTUTE Health study protocol: deliberative stakeholder engagements to inform implementation approaches to healthcare disinvestment. Implementation science : IS. 2012;7:101. doi:10.1186/1748-5908-7-101.
37. Schmidt DE. The development of a disinvestment framework to guide resource allocation decisions in health service delivery organizations. The University of British Columbia 2010. Available from: https://open.library.ubc.ca/cIRcle/collections/ubctheses/24/items/1.0073252. Accessed: 24/10/2017
38. Elshaug AG, Hiller JE, Tunis SR, Moss JR. Challenges in Australian policy processes for disinvestment from existing, ineffective health care practices. Australia and New Zealand health policy. 2007;4:23. doi:10.1186/1743-8462-4-23.
39. Rubinstein A, Belizan M, Discacciati V. Are economic evaluations and health technology assessments increasingly demanded in times of rationing health services? The case of the Argentine financial crisis. International journal of technology assessment in health care. 2007;23(2):169-76. doi:10.1017/s0266462307070274.
40. Williams I, McIver S, Moore D, Bryan S. The use of economic evaluations in NHS decision making: A review and empirical investigation. Health Technology Assessment 2008;12(7).
41. Clarke MA, Belden JL, Koopman RJ, Steege LM, Moore JL, Canfield SM et al. Information needs and information-seeking behaviour analysis of primary care physicians and nurses: a literature review. Health information and libraries journal. 2013;30(3):178-90. doi:10.1111/hir.12036.
42. Finch E, Cornwell P, Ward EC, McPhail SM. Factors influencing research engagement: research interest, confidence and experience in an Australian speech-language pathology workforce. BMC health services research. 2013;13:144.
43. Gilman IP. Evidence-based information-seeking behaviors of occupational therapists: a survey of recent graduates. Journal of the Medical Library Association : JMLA. 2011;99(4):307-10.
44. Younger P. Internet-based information-seeking behaviour amongst doctors and nurses: a short review of the literature. Health information and libraries journal. 2010;27(1):2-10. doi:10.1111/j.1471-1842.2010.00883.x.
45. Prendiville TW, Saunders J, Fitzsimons J. The information-seeking behaviour of paediatricians accessing web-based resources. Archives of disease in childhood. 2009;94(8):633-5. doi:10.1136/adc.2008.149278.
46. Davies K, Harrison J. The information-seeking behaviour of doctors: a review of the evidence. Health information and libraries journal. 2007;24(2):78-94. doi:10.1111/j.1471-1842.2007.00713.x.
47. Nail-Chiwetalu B, Bernstein Ratner N. An assessment of the information-seeking abilities and needs of practicing speech-language pathologists. Journal of the Medical Library Association : JMLA. 2007;95(2):182-8, e56-7. doi:10.3163/1536-5050.95.2.182.
48. Revere D, Turner AM, Madhavan A, Rambo N, Bugni PF, Kimball A et al. Understanding the information needs of public health practitioners: a literature review to inform design of an interactive digital knowledge management system. Journal of biomedical informatics. 2007;40(4):410-21. doi:10.1016/j.jbi.2006.12.008.
49. Chambers D, Wilson P. A framework for production of systematic review based briefings to support evidence-informed decision-making. Systematic reviews. 2012;1(1):32. doi:10.1186/2046-4053-1-32.

50. Murthy L, Shepperd S, Clarke MJ, Garner SE, Lavis JN, Perrier L et al. Interventions to improve the use of systematic reviews in decision-making by health system managers, policy makers and clinicians. Cochrane database of systematic reviews (Online). 2012;9:CD009401. doi:10.1002/14651858.CD009401.pub2.
51. Wallace J, Byrne C, Clarke M. Making evidence more wanted: a systematic review of facilitators to enhance the uptake of evidence from systematic reviews and meta-analyses. International journal of evidence-based healthcare. 2012;10(4):338-46. doi:10.1111/j.1744-1609.2012.00288.x.
52. Chambers D, Wilson PM, Thompson CA, Hanbury A, Farley K, Light K. Maximizing the impact of systematic reviews in health care decision making: a systematic scoping review of knowledge-translation resources. The Milbank quarterly. 2011;89(1):131-56. doi:10.1111/j.1468-0009.2011.00622.x.
53. LaRocca R, Yost J, Dobbins M, Ciliska D, Butt M. The effectiveness of knowledge translation strategies used in public health: a systematic review. BMC public health. 2012;12:751. doi:10.1186/1471-2458-12-751.
54. Moore G, Redman S, Haines M, Todd A. What works to increase the use of research in population health policy and programmes: a review. Evidence and Policy: A Journal of Research, Debate and Practice. 2011;7(3):277-305. doi:10.1332/174426411X579199.
55. Perrier L, Mrklas K, Lavis JN, Straus SE. Interventions encouraging the use of systematic reviews by health policymakers and managers: a systematic review. Implementation science : IS. 2011;6:43. doi:10.1186/1748-5908-6-43.
56. van Panhuis WG, Paul P, Emerson C, Grefenstette J, Wilder R, Herbst AJ et al. A systematic review of barriers to data sharing in public health. BMC public health. 2014;14:1144. doi:10.1186/1471-2458-14-1144.
57. Dawes M, Sampson U. Knowledge management in clinical practice: a systematic review of information seeking behavior in physicians. International journal of medical informatics. 2003;71(1):9-15.
58. Bowen S, Erickson T, Martens PJ, Crockett S. More than "using research": the real challenges in promoting evidence-informed decision-making. Healthcare Policy. 2009;4(3):87-102.
59. Niedzwiedzka BM. Barriers to evidence-based decision making among Polish healthcare managers. Health services management research : an official journal of the Association of University Programs in Health Administration / HSMC, AUPHA. 2003;16(2):106-15. doi:10.1258/095148403321591429.
60. Nutley T, Reynolds HW. Improving the use of health data for health system strengthening. Global health action. 2013;6:20001. doi:10.3402/gha.v6i0.20001.
61. Braa J, Heywood A, Sahay S. Improving quality and use of data through data-use workshops: Zanzibar, United Republic of Tanzania. Bulletin of the World Health Organization. 2012;90(5):379-84. doi:10.2471/blt.11.099580.
62. Robinson S, Williams I, Dickinson H, Freeman T, Rumbold B. Priority-setting and rationing in healthcare: evidence from the English experience. Social science & medicine. 2012;75(12):2386-93. doi:10.1016/j.socscimed.2012.09.014.
63. Cornelissen E, Mitton C, Davidson A, Reid RC, Hole R, Visockas AM et al. Changing priority setting practice: The role of implementation in practice change. Health policy (Amsterdam, Netherlands). 2014. doi:10.1016/j.healthpol.2014.04.010.
64. Vest JR, Kirk HM, Issel LM. Quality and integration of public health information systems: A systematic review focused on immunization and vital records systems. Online journal of public health informatics. 2012;4(2). doi:10.5210/ojphi.v4i2.4198.
65. Jorm L. Routinely collected data as a strategic resource for research: priorities for methods and workforce. Public health research & practice. 2015;25(4):e2541540. doi:10.17061/phrp2541540.
66. Stansfield SK, Walsh J, Prata N, Evans T. Information to Improve Decision Making for Health. In: Jamison DT, Breman JG, Measham AR, Alleyne G, Claeson M, Evans DB et al., editors. Disease Control Priorities in Developing Countries. Washington DC: The International Bank for Reconstruction and Development/The World Bank Group; 2006.
67. Mitton C, Dionne F, Donaldson C. Managing healthcare budgets in times of austerity: the role of program budgeting and marginal analysis. Applied health economics and health policy. 2014;12(2):95-102. doi:10.1007/s40258-013-0074-5.
68. Morrato EH, Elias M, Gericke CA. Using population-based routine data for evidence-based health policy decisions: lessons from three examples of setting and evaluating national health policy in Australia, the UK and the USA. Journal of public health (Oxford, England). 2007;29(4):463-71. doi:10.1093/pubmed/fdm065.
69. Riley BL, Robinson KL, Gamble J, Finegood DT, Sheppard D, Penney TL et al. Knowledge to action for solving complex problems: insights from a review of nine international cases. Health promotion and chronic disease prevention in Canada. 2015;35(3):47-53.
70. Giles-Corti B, Sallis JF, Sugiyama T, Frank LD, Lowe M, Owen N. Translating active living research into policy and practice: One important pathway to chronic disease prevention. Journal of public health policy. 2015;36(2):231-43. doi:10.1057/jphp.2014.53.
71. Bowers B, Cohen LW, Elliot AE, Grabowski DC, Fishman NW, Sharkey SS et al. Creating and supporting a mixed methods health services research team. Health services research. 2013;48(6 Pt 2):2157-80. doi:10.1111/1475-6773.12118.
72. Young T, Rohwer A, Volmink J, Clarke M. What Are the Effects of Teaching Evidence-Based Health Care (EBHC)? Overview of Systematic Reviews. PLoS One. 2014;9(1):e86706. doi:10.1371/journal.pone.0086706.
73. O'Leary D F, Mhaolrunaigh SN. Information-seeking behaviour of nurses: where is information sought and what processes are followed? Journal of advanced nursing. 2012;68(2):379-90. doi:10.1111/j.1365-2648.2011.05750.x.
74. Hider PN, Griffin G, Walker M, Coughlan E. The information-seeking behavior of clinical staff in a large health care organization. Journal of the Medical Library Association : JMLA. 2009;97(1):47-50. doi:10.3163/1536-5050.97.1.009.
75. Kloda LA, Bartlett JC. Clinical information behavior of rehabilitation therapists: a review of the research on occupational therapists, physical therapists, and speech-language pathologists. Journal of the Medical Library Association : JMLA. 2009;97(3):194-202.

76. Coumou HC, Meijman FJ. How do primary care physicians seek answers to clinical questions? A literature review. Journal of the Medical Library Association : JMLA. 2006;94(1):55-60.
77. Kostagiolas PA, Aggelopoulou VA, Niakas D. A study of the information seeking behaviour of hospital pharmacists: empirical evidence from Greece. Health information and libraries journal. 2011;28(4):302-12. doi:10.1111/j.1471-1842.2011.00963.x.
78. Hadley JA, Wall D, Khan KS. Learning needs analysis to guide teaching evidence-based medicine: knowledge and beliefs amongst trainees from various specialities. BMC medical education. 2007;7:11. doi:10.1186/1472-6920-7-11.
79. Harris C, Allen K, Brooke V, Dyer T, Waller C, King R et al. Sustainability in Health care by Allocating Resources Effectively (SHARE) 6: Investigating methods to identify, prioritise, implement and evaluate disinvestment projects in a local healthcare setting. BMC health services research. 2017. doi:10.1186/s12913-017-2269-1.
